# Supplementary material for: Primary care system-level training and support programme for the secondary prevention of domestic violence and abuse: a cost-effectiveness feasibility model
Source: BMJ Open. 2024 Jan 6;14(1):e071300. doi: 10.1136/bmjopen-2022-071300 (PMC10773355; doi:10.1136/bmjopen-2022-071300)
Supplement: Supplementary data [file bmjopen-2022-071300supp001.pdf]

Primary care system-level training and support programme for the secondary prevention of domestic violence and abuse: a cost-effectiveness feasibility model

Madeleine Cochrane, Eszter Szilassy, Caroline Coope, Elizabeth Emsley, Medina Johnson, Gene Feder and Estela Capelas Barbosa

Table S1. Adult's model input parameters: probabilities; utilities; and, costs.

Parameter	Base case value	Lower limit	Upper limit	Distribution	Source
Probabilities					
Prevalence of DVA in adults (males and females) – aged 16 to 65	0.055	0.036	0.073	Beta	ONS, 2020b
Starting distribution for patients who are abused					
Abused and identified, seeing advocate	0.003	0	0.0066	Uniform	*
Abused and identified, not seeing advocate	0.033	0	0.0660	Uniform	*
Abused but not identified	0.964	-	-	Uniform	Complement
Transition probabilities					
Not abused to Abused but not identified	0.0037	0.0004	0.0106	Dirichlet	*
Not abused to Dead	0.0052	0.0027	0.0087	Dirichlet	ONS, 2020b
Stay in Not abused	0.9911	-	-	Dirichlet	Complement
Abused but not identified to Not abused (control)	0.0500	0.0412	0.0596	Dirichlet	*
Abused but not identified to Abused and identified, not seeing advocate (control)	0.0091	0.0055	0.0135	Dirichlet	IRIS data

Abused but not identified to Abused and identified, seeing advocate (control)	0.0226	0.0168	0.0293	Dirichlet	IRIS data
Abused but not identified to Dead (control)	0.0055	0.0029	0.0091	Dirichlet	ONS, 2020b
Stay in Abused but not identified (control)	0.9131	-	-	Dirichlet	Complement
Abused but not identified to Not abused (intervention)	0.0500	0.0412	0.0596	Dirichlet	*
Abused but not identified to Abused and identified, not seeing advocate (intervention)	0.0374	0.0298	0.0458	Dirichlet	IRIS+ data
Abused but not identified to Abused and identified, seeing advocate (intervention)	0.0312	0.0243	0.0390	Dirichlet	IRIS+ data
Abused but not identified to Dead (intervention)	0.0055	0.0029	0.0091	Dirichlet	ONS, 2020B
Stay in Abused but not identified (intervention)	0.8762	-	-	Dirichlet	Complement
Abused and identified, seeing advocate to Not abused	0.1408	0.0707	0.2301	Dirichlet	(Taft et al., 2011)
Abused and identified, seeing advocate to Dead	0.0052	0.0000	0.0299	Dirichlet	ONS, 2020b
Stay in Abused and identified, seeing advocate	0.854	-	-	Dirichlet	Complement
Abused and identified, not seeing advocate to Not abused	0.0781	0.0136	0.1912	Dirichlet	(Taft et al., 2011)
Abused and identified, not seeing advocate to Dead	0.0052	0.0000	0.0424	Dirichlet	ONS, 2020b

Stay in Abused and identified, not seeing advocate	0.9167	-	-	Dirichlet	Complement
Utilities					
Not abused (adults)	0.850	0.840	0.860	Beta	(Kind et al., 1999)
Abused but not identified (women)	0.656	0.522	0.749	Beta	Assumption
Abused but not identified (men)	0.626	0.500	0.744	Beta	Assumption
Abused and identified, seeing advocate (women)	0.659	0.518	0.782	Beta	IRIS+ data
Abused and identified, seeing advocate (men)	0.701	0.555	0.828	Beta	IRIS+ data
Abused and identified, not seeing advocate (women)	0.656	0.522	0.749	Beta	IRIS+ data
Abused and identified, not seeing advocate (men)	0.626	0.500	0.744	Beta	IRIS+ data
Costs (2019/20£)					
Costs of the intervention, per patient exposed to DV, per 6 months	£0.75	£0.02	£2.73	Gamma	IRIS+ budget
Cost of onward referral, once	£658	£11	£1908	Gamma	IRIS+ data; IRIS data
Cost of Abused but not identified (adults)	£4858	£123	£17919	Gamma	(Oliver et al., 2019)
Weighted costs Abused and identified, seeing advocate	1	0.75	1.25	Gamma	Assumption
Weighted costs Abused and identified, not seeing advocate	1	0.9	1.1	Gamma	Assumption

Costs are in 2019/20 UK£.
* Internal calculation based on model calibration.

± Excludes the cost of harms, which in this modelled are measured as benefit

Table S2. Children's model input parameters: probabilities; utilities; and, costs.

Parameter	Base case value	Lower limit	Upper limit	Distribution	Source
Probabilities					
Prevalence of children exposed to DVA	0.080	0.040	0.140	Beta	(Gilbert et al., 2009)
Starting distribution for patients who are abused					
Abused and identified, seeing advocate	0.003	0	0.0066	Uniform	*
Abused and identified, not seeing advocate	0.033	0	0.0660	Uniform	*
Abused but not identified	0.964	-	-	Uniform	Complement
Transition probabilities					
Not abused to Abused but not identified	0.0037	0.0004	0.0106	Dirichlet	*
Not abused to Dead	0.0052	0.0027	0.0087	Dirichlet	ONS, 2020b
Stay in Not abused	0.9911	-	-	Dirichlet	Complement
Abused but not identified to Not abused (control)	0.0500	0.0412	0.0596	Dirichlet	*

Abused but not identified to Abused and identified, not seeing advocate (control)	0.0091	0.0055	0.0135	Dirichlet	IRIS data
Abused but not identified to Abused and identified, seeing advocate (control)	0.0226	0.0168	0.0293	Dirichlet	IRIS data
Abused but not identified to Dead (control)	0.0055	0.0029	0.0091	Dirichlet	ONS, 2020b
Stay in Abused but not identified (control)	0.9131	-	-	Dirichlet	Complement
Abused but not identified to Not abused (intervention)	0.0500	0.0412	0.0596	Dirichlet	*
Abused but not identified to Abused and identified, not seeing advocate (intervention)	0.0374	0.0298	0.0458	Dirichlet	IRIS+ data
Abused but not identified to Abused and identified, seeing advocate (intervention)	0.0312	0.0243	0.0390	Dirichlet	IRIS+ data
Abused but not identified to Dead (intervention)	0.0055	0.0029	0.0091	Dirichlet	ONS, 2020b
Stay in Abused but not identified (intervention)	0.8762	-	-	Dirichlet	Complement
Abused and identified, seeing advocate to Not abused	0.1408	0.0707	0.2301	Dirichlet	(Taft et al., 2011)
Abused and identified, seeing advocate to Dead	0.0052	0.0000	0.0299	Dirichlet	ONS, 2020b
Stay in Abused and identified, seeing advocate	0.854	-	-	Dirichlet	Complement

Abused and identified, not seeing advocate to Not abused	0.0781	0.0136	0.1912	Dirichlet	(Taft et al., 2011)
Abused and identified, not seeing advocate to Dead	0.0052	0.0000	0.0424	Dirichlet	ONS, 2020b
Stay in Abused and identified, not seeing advocate	0.9167	-	-	Dirichlet	Complement
Utilities					
Not abused (children)	0.950	0.940	0.959	Beta	(Kind et al., 1999)
Abused but not identified (children)	0.801	0.623	0.932	Beta	Assumption
Abused and identified, seeing advocate (children)	0.804	0.625	0.935	Beta	IRIS+ data
Abused and identified, not seeing advocate (children)	0.801	0.623	0.932	Beta	IRIS+ data
Costs (2019/20£)					
Costs of the intervention, per patient exposed to DV, per 6 months	£0.75	£0.02	£2.73	Gamma	IRIS+ budget
Cost of onward referral, once	£658	£11	£1908	Gamma	IRIS+ data; IRIS data
Cost of Abused but not identified (children)	£1950	£1000	£2500	Gamma	(Pro Bono Economics, 2018)
Weighted costs Abused and identified, seeing advocate	1	0.75	1.25	Gamma	Assumption
Weighted costs Abused and identified, not seeing advocate	1	0.9	1.1	Gamma	Assumption

Costs are in 2019/20 UK£. * Internal calculation based on model calibration. ± Excludes the cost of harms, which in this modelled are measured as benefit
