# Supplementary material for: Primary care system-level training and support programme for the secondary prevention of domestic violence and abuse: a cost-effectiveness feasibility model
Source: BMJ Open. 2024 Jan 6;14(1):e071300. doi: 10.1136/bmjopen-2022-071300 (PMC10773355; doi:10.1136/bmjopen-2022-071300)
Supplement: Author's manuscript [file bmjopen-2022-071300.draft_revisions.pdf]

BMJ Open is committed to open peer review. As part of this commitment we make the peer review history of every article we publish publicly available.

When an article is published we post the peer reviewers' comments and the authors' responses online. We also post the versions of the paper that were used during peer review. These are the versions that the peer review comments apply to.

The versions of the paper that follow are the versions that were submitted during the peer review process. They are not the versions of record or the final published versions. They should not be cited or distributed as the published version of this manuscript.

BMJ Open is an open access journal and the full, final, typeset and author-corrected version of record of the manuscript is available on our site with no access controls, subscription charges or pay-per-view fees (http://bmjopen.bmj.com).

If you have any questions on BMJ Open's open peer review process please email info.bmjopen@bmj.com

BMJ Open

# Primary care system-level training and support programme for the secondary prevention of domestic violence and abuse: a cost-effectiveness feasibility model

| | |
|---|---|
| Journal: | *BMJ Open* |
| Manuscript ID | bmjopen-2022-071300 |
| Article Type: | Original research |
| Date Submitted by the Author: | 06-Jan-2023 |
| Complete List of Authors: | Cochrane, Madeleine; University of Bristol,<br>Szilassy, Eszter; University of Bristol<br>Coope, Caroline; University of Bristol<br>Emsley, Elizabeth; University of Bristol<br>Johnson, Medina; IRISi<br>Feder, Gene; University of Bristol<br>Barbosa, Estela; City University of London |
| Keywords: | PRIMARY CARE, HEALTH ECONOMICS, PUBLIC HEALTH |
| | |

SCHOLARONE™
Manuscripts

*I, the Submitting Author has the right to grant and does grant on behalf of all authors of the Work (as defined in the below author licence), an exclusive licence and/or a non-exclusive licence for contributions from authors who are: i) UK Crown employees; ii) where BMJ has agreed a CC-BY licence shall apply, and/or iii) in accordance with the terms applicable for US Federal Government officers or employees acting as part of their official duties; on a worldwide, perpetual, irrevocable, royalty-free basis to BMJ Publishing Group Ltd ("BMJ") its licensees and where the relevant Journal is co-owned by BMJ to the co-owners of the Journal, to publish the Work in this journal and any other BMJ products and to exploit all rights, as set out in our licence.*

*The Submitting Author accepts and understands that any supply made under these terms is made by BMJ to the Submitting Author unless you are acting as an employee on behalf of your employer or a postgraduate student of an affiliated institution which is paying any applicable article publishing charge ("APC") for Open Access articles. Where the Submitting Author wishes to make the Work available on an Open Access basis (and intends to pay the relevant APC), the terms of reuse of such Open Access shall be governed by a Creative Commons licence – details of these licences and which Creative Commons licence will apply to this Work are set out in our licence referred to above.*

*Other than as permitted in any relevant BMJ Author's Self Archiving Policies, I confirm this Work has not been accepted for publication elsewhere, is not being considered for publication elsewhere and does not duplicate material already published. I confirm all authors consent to publication of this Work and authorise the granting of this licence.*

For peer review only

**Title Page**

# Primary care system-level training and support programme for the secondary prevention of domestic violence and abuse: a cost-effectiveness feasibility model

Madeleine Cochrane, Eszter Szilassy, Caroline Coope, Elizabeth Emsley, Medina Johnson, Gene Feder and Estela Capelas Barbosa

**Correspondence**

*Corresponding author:*

Madeleine Cochrane: madeleine.cochrane@bristol.ac.uk

Population Health Sciences, Bristol Medical School, University of Bristol, 1-5 Whiteladies Road, Bristol, BS8 1NU, UK. Tel: 0117 428 3116

*Co-authors:*

Eszter Szilassy: eszter.szilassy@bristol.ac.uk

Caroline Coope: caroline.coope@bristol.ac.uk

Elizabeth Emsley: elizabeth.emsley@bristol.ac.uk

Medina Johnson: medina.johnson@irisi.org

Gene Feder: gene.feder@bristol.ac.uk

Estela Capelas Barbosa: estela.barbosa@city.ac.uk

Population Health Sciences, Bristol Medical School, University of Bristol, 1-5 Whiteladies Road, Bristol, BS8 1NU, UK. Eszter Szilassy, Caroline Coope, Elizabeth Emsley, Gene Feder.

City University of London, Northampton Square, London, EC1V 0HB. UK. Estela Capelas Barbosa

IRISi, London, UK. Medina Johnson.

For peer review only

**Contribution statement**

Cochrane attests that all listed authors meet authorship criteria and that no others meeting the criteria have been omitted from the opportunity to be listed as an author. All authors assisted with the study design. MC and ECB conducted the analysis for the economic evaluation. MC and ECB developed the manuscript for the economic evaluation. All authors read and commented on manuscript drafts and approved the final manuscript.

**Competing interests**

Medina Johnson is the CEO of IRISi and was a named partner in REPROVIDE. She did not influence the economic modelling or its results.

**Funding**

IRIS+ is part of the REPROVIDE programme (Reaching Everyone Programme of Research On Violence in diverse Domestic Environments), an independent research programme funded by the National Institute for Health Research (Programme Grants for Applied Research), (RP-PG-0614-20012). The views expressed in this publication are those of the authors and not necessarily those of the National Institute for Health Research or the Department of Health and Social Care. The funder had no role in the design and conduct of the study; collection, management, analysis, and interpretation of the data; preparation, review, or approval of the manuscript; and decision to submit the manuscript for publication.

**Data sharing and data availability statement**

All data requests should be submitted to the corresponding author for consideration. Access to anonymised data may be granted following review.

For peer review only

**Ethics approval statement**

The study was given favourable ethical approval by London - Hampstead Research Ethics Committee (REC reference: 19/LO/1132) and the Health Research Authority (HRA) and Health and Care Research Wales (HCRW).

**Keywords**

Domestic violence, training programme, general practice, primary care, cost-effectiveness.

**Word count:** 3,358

For peer review only



For peer review only - http://bmjopen.bmj.com/site/about/guidelines.xhtml

## Abstract

**Objectives:** To evaluate the prospective cost-effectiveness of the Identification and Referral to Improve Safety Plus (IRIS+) intervention compared to usual care using feasibility data derived from seven UK GP practice sites.

**Method:** A cost-utility analysis was conducted to assess the potential cost-effectiveness of IRIS+, an enhanced model of the UK's usual care. IRIS+ assisted primary care staff in identifying, documenting and referring not only women, but also men and children who may have experienced domestic violence/ abuse as victims, perpetrators, or both. A Markov model was constructed from a societal perspective to estimate mean incremental costs and quality-adjusted life-years (QALYs) of IRIS+ compared to IRIS over a 10 year time horizon.

**Results:** The IRIS+ programme saved £93 per patient and produced QALY gains of 0.003. The incremental net monetary benefit was positive (£145) and the IRIS+ programme was cost-effective in 55% of simulations when the cost-effectiveness threshold was £20,000.

**Conclusion:** The IRIS+ programme is likely to be cost-effective or even cost-saving from a societal perspective in the UK, though there are large uncertainties, reflected in the confidence intervals.


For peer review only



For peer review only - http://bmjopen.bmj.com/site/about/guidelines.xhtml

**Strengths and Limitations**

- To the authors knowledge this is the first study to assess the potential cost-effectiveness of a primary care intervention providing support to all women, men and children experiencing domestic violence/ abuse
- The study draws on the structure of a previous domestic violence/ abuse model which has been published in peer-reviewed journals
- The study relies on newly collected data, reducing the need for using out-of-date previously published estimates
- The small number of newly collected data, means our results may not be representative of the wider UK population



For peer review only - http://bmjopen.bmj.com/site/about/guidelines.xhtml

## Background

Domestic violence/ abuse (DVA) is a public health challenge, affecting approximately 9 million adults and 2 million children in the UK (1-4). The societal cost of DVA was estimated by the UK Home Office to be £66bn in 2017, not including costs to children. Safe Lives, a UK-wide DVA charity, highlighted the need for an initial £2.2bn of public investment per annum to cover domestic abuse services for the whole family – adult, teen and child victims, and perpetrators (5, 6). Public Health England identified primary care as a key location for interventions to prevent DVA and improve health outcomes for adults and children. Early intervention in DVA, for example, in the primary care setting, reduces the overall public service burden of abuse and can reduce escalation of violence (7).

DVA interventions to date have prioritised women, who are disproportionately affected in prevalence and severity of DVA, compared with other groups (8, 9). Identifying female survivors in primary care and referring to specialist support is effective and cost-effective through provision of DVA training linked with a direct pathway to local DVA support (10). The leading service model in the UK's NHS primary care setting is IRIS (Identification and Referral to Improve Safety), a widely commissioned evidence-based DVA training and advocacy support programme for female survivors.

While there is growing success in identifying women affected by DVA, male survivors and children/ young people (CYP) are rarely identified in primary care and referred for specialist support. This neglects the mental and physical health impact across the life-course for CYP who experience or witness DVA (11, 12) and the significant mental health impact on men exposed to DVA (13-16). IRIS plus (IRIS+) was an enhanced model of the existing IRIS programme and was piloted in NHS primary care GP sites, three sites in England and four sites in Wales. IRIS+ assisted GP practice staff in identifying, documenting and referring not only



For peer review only - http://bmjopen.bmj.com/site/about/guidelines.xhtml

women, but also men and children who may have experienced DVA as victims, perpetrators, or both. The IRIS+ pilot study showed feasibility and acceptability of the intervention to clinicians and those affected by DVA (17, 18). The aim of this study was to evaluate the prospective cost-effectiveness of the IRIS+ programme when compared to the IRIS programme. This study addresses a gap in the literature around the possible cost-effectiveness of interventions targeting men and children as well as women experiencing DVA.

## Methods

*Overview of economic evaluation*

This study was a model-based cost–utility analysis, comparing the IRIS+ intervention to the IRIS intervention. An unpublished health economic analysis plan (HEAP) was developed prospectively to guide the economic evaluation. The outcome measure was quality-adjusted life years (QALYs), which are the recommended outcomes for economic evaluations in the UK (19). As many of the costs of DVA are borne outside the health system, the analysis was undertaken from a UK societal perspective which in this study we define as the costs associated with implementing the intervention, downstream multi-sector costs associated with DVA, as well as productivity costs. Costs were calculated in 2019/20 UK£, as most of the IRIS+ intervention took place in those years. Costs and benefits were calculated over a 10 year time horizon. This was considered appropriate because the occurrence of new cases and transition probabilities were assumed to remain constant over time and therefore the impact of a longer time horizon would be small. While this is likely to be the case for adults, we acknowledge that the time horizon for children may be longer (20). This means we opted for a conservative estimate of the cost-effectiveness of the intervention as far as children are concerned. Future costs and outcomes were discounted at an annual rate of 3.5% as recommended in the UK guidelines for conducting economic evaluations (19) .

For peer review only



For peer review only - http://bmjopen.bmj.com/site/about/guidelines.xhtml

*Model structure*

We developed a Markov model based on the previous analysis of the cost-effectiveness of IRIS (21, 22). The model has five health states (see Figure 1 for details) and the cycle length was six months, which reflects the average length of support received from advocacy services following referral. Other than death, which is an absorbing state, men, women and children can transition between states in half-yearly cycles. The states were 'Not abused', 'Abused but not identified', 'Abused and identified, seeing advocate', 'Abuse and identified, not seeing advocate' and 'Dead' (Figure 1). A hypothetical cohort of 10,000 people was simulated moving between the states (Figure 1). We used the Census figures to estimate proportion of adult men, women and children within this hypothetical cohort (14).

*Interventions*

*The IRIS Programme (control)*

The IRIS programme is a multi-component intervention which has been described elsewhere (21, 23). In short, it is delivered in UK NHS primary care GP sites and consists of multidisciplinary training sessions, targeted at the clinical team and some general practice reception staff. The training sessions were designed to address barriers to improving the response of clinicians to women experiencing abuse through improved identification, support and referral to specialist agencies. Clinicians are trained to have a low threshold for asking about DVA. Training incorporates case studies and practice in asking about violence and responding appropriately. They are delivered by an advocate educator from collaborating specialist support services. The advocate educator is central to the IRIS intervention, combining a training and support role to the practices with provision of advocacy to women referred. Ongoing support to clinicians and reception staff in the practices is provided by the advocate educator.



For peer review only - http://bmjopen.bmj.com/site/about/guidelines.xhtml

*The IRIS+ intervention (intervention)*

The IRIS+ intervention builds on the IRIS model, but in addition provides a service for men and children. Similar to IRIS, it consists of a multi-component intervention, including multidisciplinary training for clinicians and general practice staff. IRIS+ provides a simple pathway of referrals to specialist support services for women, men and children who experience (survivors and perpetrators) DVA. In IRIS+, as well as the advocate educator, there is a dedicated children's worker. Jointly they support any referral made by clinicians, regardless of gender or age.

*Probabilities*

Whenever possible, we used data collected in the pilot to estimate transition probabilities required for the Markov model. Where this was not possible probabilities were obtained from published sources. Table 1 shows the source of data for each relevant parameter.

For peer review only



For peer review only - http://bmjopen.bmj.com/site/about/guidelines.xhtml

**Table 1. Model input parameters: probabilities**

| Parameter | Base case value | Lower limit | Upper limit | Distribution | Source |
|---|---|---|---|---|---|
| ***Probabilities*** | | | | | |
| Proportion of patients experiencing abuse | 0.055 | 0.038 | 0.106 | Beta | **adjusted estimate |
| Prevalence of DVA in adults (males and females) – aged 16 to 65 | 0.055 | 0.036 | 0.073 | Beta | (14) |
| Prevalence of children exposed to DVA | 0.080 | 0.040 | 0.140 | Beta | (24) |
| *Starting distribution for those experiencing abuse* | | | | | |
| Abused and identified, seeing advocate | 0.003 | 0 | 0.0066 | Uniform | * |
| Abused and identified, not seeing advocate | 0.033 | 0 | 0.0660 | Uniform | * |
| Abused but not identified | 0.964 | - | - | Uniform | Complement |
| *Transition probabilities* | | | | | |
| Not abused to Abused but not identified | 0.0037 | 0.0004 | 0.0106 | Dirichlet | * |
| Not abused to Dead | 0.0052 | 0.0027 | 0.0087 | Dirichlet | (14) |
| Stay in Not abused | 0.9911 | - | - | Dirichlet | Complement |
| Abused but not identified to Not abused (control) | 0.0500 | 0.0412 | 0.0596 | Dirichlet | * |
| Abused but not identified to Abused and identified, not seeing advocate (control) | 0.0091 | 0.0055 | 0.0135 | Dirichlet | IRIS data |



For peer review only - http://bmjopen.bmj.com/site/about/guidelines.xhtml

| | | | | | |
|---|---|---|---|---|---|
| Abused but not identified to Abused and identified, seeing advocate (control) | 0.0226 | 0.0168 | 0.0293 | Dirichlet | IRIS data |
| Abused but not identified to Dead (control) | 0.0055 | 0.0029 | 0.0091 | Dirichlet | (14) |
| Stay in Abused but not identified (control) | 0.9131 | - | - | Dirichlet | Complement |
| Abused but not identified to Not abused (intervention) | 0.0500 | 0.0412 | 0.0596 | Dirichlet | * |
| Abused but not identified to Abused and identified, not seeing advocate (intervention) | 0.0374 | 0.0298 | 0.0458 | Dirichlet | IRIS+ data |
| Abused but not identified to Abused and identified, seeing advocate (intervention) | 0.0312 | 0.0243 | 0.0390 | Dirichlet | IRIS+ data |
| Abused but not identified to Dead (intervention) | 0.0055 | 0.0029 | 0.0091 | Dirichlet | (14) |
| Stay in Abused but not identified (intervention) | 0.8762 | - | - | Dirichlet | Complement |
| Abused and identified, seeing advocate to Not abused | 0.1408 | 0.0707 | 0.2301 | Dirichlet | (25) |
| Abused and identified, seeing advocate to Dead | 0.0052 | 0.0000 | 0.0299 | Dirichlet | (14) |
| Stay in Abused and identified, seeing advocate | 0.854 | - | - | Dirichlet | Complement |
| Abused and identified, not seeing advocate to Not abused | 0.0781 | 0.0136 | 0.1912 | Dirichlet | (25) |
| Abused and identified, not seeing advocate to Dead | 0.0052 | 0.0000 | 0.0424 | Dirichlet | (14) |
| Stay in Abused and identified, not seeing advocate | 0.9167 | - | - | Dirichlet | Complement |

* Internal calculation based on model calibration



For peer review only - http://bmjopen.bmj.com/site/about/guidelines.xhtml

*Prevalence of domestic abuse*

The proportion of adults aged 16 years or older experiencing abuse was estimated from the Crime Survey for England and Wales (CSEW) (14). There was a subsequent published estimate, but due to anti-contagion measures relating to the COVID-19 pandemic, the survey had to be moved to telephone survey, preventing some of the collection of relevant data on domestic violence. In 2018/19, 5.5% of adults experienced some form of domestic violence according to the CSEW (14). Since IRIS+ also provides support services for children, we relied on the published estimate of 8% of children experiencing some form of DVA in the past 12 months (24). Children represent 20% of the UK population (26). To extrapolate beyond age 65 we used data from the USA showing that the prevalence of intimate partner violence was 2.2% among people aged 65 or older (27). We therefore estimate that 5.5% of the UK population would be in any of the three states in the Markov model associated with abuse in the first model cycle.

*Transition probabilities*

Table 1 reports all transition probabilities. There are eight transitions between states in the model, measured as follows:

1. Not abused to Abused but not identified

No data were available to reliably estimate this probability. We thus estimated it using the model calibration method described below.

2. Abused but not identified to Abused and identified, seeing advocate

For those receiving the IRIS+ intervention, we estimated this transition probability based on the number of patients seen by the advocate in IRIS+ pilot. Dividing this number by the total number of eligible patients in the seven GP practices, gives a six-month transition probability.



For peer review only - http://bmjopen.bmj.com/site/about/guidelines.xhtml

For the control practices, this probability was estimated based on the number of women only referred to IRIS advocacy.

3. Abused but not identified to Abused and identified, not seeing advocate

We used the ratio of the number of patients abused and identified vs referred in the IRIS+ pilot to estimate the number of patients abused and identified. These were effectively patients referred who decline support or who could not be contacted following the referral. The transition probability for the control group (IRIS Programme) was calculated as above, but only considered women identified vs referred.

4. Abused but not identified to Not abused

No data was available to reliably estimate this probability. We therefore estimated this using the model calibration method described below.

5. Abused and identified, seeing advocate to Not abused

This was taken from the MOSAIC (mothers' advocates in the community) trial (25), identified in a Cochrane review (28), evaluating the reduction of any type of domestic abuse with any type of advocacy.

6. Abused and identified, not seeing advocate to Not abused

This was taken from the control arm of the MOSAIC trial (25).

7. Not abused to Dead

We relied on the death rate per 1000 as estimated by the Office for National Statistics. For 2019, it was estimated at 10.4 per 1000. This implies the probability of dying per 6 months at 5.2 per 1000 people, excluding domestic homicides.

8. Abused to Dead

For patients experiencing abuse this probability was 5.54 per 1000 (figure including domestic homicides).



For peer review only - http://bmjopen.bmj.com/site/about/guidelines.xhtml

*Model calibration*

We used the prevalence of abuse (5.5%) to calibrate the model, since there was uncertainty surrounding transition probabilities 'Not abused to Abused but not identified' and vice versa. The calibration was run for 3000 cycles, assuming that after this, the number of patients in each state would remain constant. The transition probabilities 'Not abused to Abused but not identified' and vice versa were changed until the proportion of patients in the 'Not abused' state exactly reflected the observed prevalence (100-5.5=94.5%). The initial distribution of patients in the three 'Abused' states was also determined by this process.

*Utilities*

Each state in the Markov model was associated with a utility score (Table 2), allowing us to measure QALYs associated with IRIS+ and the comparator (IRIS) based on the proportion of patients in each health state in each of the 20 cycles in the model. Utility scores were separately collected and calculated for men, women, and children. For the health state 'No abuse' the utility was assumed to be 0.85 for adults and 0.95 for children, following published population norms (29). A subset of adults and children identified from the IRIS+ pilot filled in a SF-12 and CHU-9D form, respectively. If support/ advocacy was accepted, questionnaire data were requested at: (1) baseline, defined as when support/ advocacy started; and (2) between 6-10 months follow up, defined as the period when support/ advocacy ended. A validated mapping algorithm was used to transform SF-12 scores to SF-6D utilities (30). The published SF-6D utilities were derived from a representative sample (n=611) of the UK adult population using the standard gamble valuation method. Similarly, a published value set was used to transform CHU-9D scores into utilities (31). The published CHU-9D value set was derived from members of the UK adult population (n=300) using both standard gamble and ranking valuation methods. Estimated scores at baseline were attributed to 'Abuse identified, not seeing



For peer review only - http://bmjopen.bmj.com/site/about/guidelines.xhtml

advocate'. Follow up scores were attributed to 'Abuse identified, seeing advocate'. Due to the small number of forms collected (n=30 at baseline; n=16 at follow up), this data was compared with previous literature for women for sense checking (32). For 'Abuse unidentified', we assumed the utility score was the same as 'Abuse identified, not seeing advocate', based on the assumption that identification alone (with advocacy support) does not improve quality of life.

*Costs*

We included: intervention costs; costs of onward referral; and costs associated with abuse (including costs to the UK National Health Service (NHS), costs of lost economic output, costs to the criminal/civil justice system, personal costs) (Table 2). Intervention costs were taken from the budget of the programme. The cost of onward referral considered the time an advocate educator or a children worker may spend working with external agencies, where their support alone would not suffice.

Costs associated with domestic violence in the UK for people aged 16+ is described in Oliver et al (33). In this report, costs of lost economic output, health services, criminal justice system, civil justice system, social welfare, personal costs, specialised services and physical/emotional harm were included, and unit cost per victim per year is estimated at £34,015 (in 2019 prices). We excluded costs of physical/ emotional harm (£24,300). Thus, for adults, the cost of abuse per 6 months was £4,858. For children, we relied on a report produced by Pro Bono Economics (20), which estimated the cost of domestic violence per child to be £1,950 in 2018£. We inflated this estimate, considered children to account for 20% of the UK population and estimated an overall cost of abuse of £4,276 per patient per 6 months.



For peer review only - http://bmjopen.bmj.com/site/about/guidelines.xhtml

**Table 2. Model input parameters: utilities and costs**

| Parameter | Base case value | Lower limit | Upper limit | Distribution | Source |
|---|---|---|---|---|---|
| ***Utilities*** | | | | | |
| Not abused (adults) | 0.850 | 0.840 | 0.860 | Beta | (29) |
| Not abused (children) | 0.950 | 0.940 | 0.959 | Beta | (29) |
| Abused but not identified (women) | 0.656 | 0.522 | 0.749 | Beta | Assumption |
| Abused but not identified (men) | 0.626 | 0.500 | 0.744 | Beta | Assumption |
| Abused but not identified (children) | 0.801 | 0.623 | 0.932 | Beta | Assumption |
| Abused and identified, seeing advocate (women) | 0.659 | 0.518 | 0.782 | Beta | IRIS+ data |
| Abused and identified, seeing advocate (men) | 0.701 | 0.555 | 0.828 | Beta | IRIS+ data |
| Abused and identified, seeing advocate (children) | 0.804 | 0.625 | 0.935 | Beta | IRIS+ data |
| Abused and identified, not seeing advocate (women) | 0.656 | 0.522 | 0.749 | Beta | IRIS+ data |
| Abused and identified, not seeing advocate (men) | 0.626 | 0.500 | 0.744 | Beta | IRIS+ data |
| Abused and identified, not seeing advocate (children) | 0.801 | 0.623 | 0.932 | Beta | IRIS+ data |
| ***Costs (2019/20£)*** | | | | | |
| Costs of the intervention, per patient exposed to DV, per 6 months | £0.74 | £0.02 | £2.73 | Gamma | IRIS+ budget |



For peer review only - http://bmjopen.bmj.com/site/about/guidelines.xhtml

| | | | | | |
|---|---|---|---|---|---|
| Cost of onward referral, once | £658 | £11 | £1908 | Gamma | IRIS+ data and IRIS data |
| Cost of Abused but not identified (weighted average – exposed population) | £4276 | £108 | £15774 | Gamma | **weighted average |
| Cost of Abused but not identified (adults) | £4858 | £123 | £17919 | Gamma | (33) |
| Cost of Abused but not identified (children) | £1950 | £1000 | £2500 | Gamma | (20) |
| Weighted costs Abused and identified, seeing advocate | 1 | 0.75 | 1.25 | Gamma | Assumption |
| Weighted costs Abused and identified, not seeing advocate | 1 | 0.9 | 1.1 | Gamma | Assumption |

Costs are in 2019/20 UK£. **Excludes the cost of harms, which in this modelled are measured as benefits

For peer review only



For peer review only - http://bmjopen.bmj.com/site/about/guidelines.xhtml

*Cost-utility analysis*

A cost-utility analysis was conducted comparing costs and QALYs for IRIS+ versus IRIS. QALYs were calculated from utilities by using the area under the curve approach. The main outcome was the Net Monetary benefit, that estimates both costs and QALYs in monetary terms, using an acceptability threshold of £20,000 per QALY. A positive incremental net monetary benefit (NMB) result indicates that IRIS+ programme would be preferred on cost-effectiveness grounds. While a negative incremental NMB result indicates that the IRIS Programme (control) would be preferred. Results were also shown in terms of the incremental costs per QALY gained of IRIS+ vs. the IRIS. This was measured as the difference in costs between intervention and control groups divided by the difference in QALYs. We followed the usual decision making rule for cost-effectiveness in the UK, in which an intervention considered cost-effective when the incremental costs per QALY gained are less than £20,000 (19).

*Subgroups and distributional effects*

The intervention and control group represented two key groups which could be targeted in primary care (women, men and children vs women only). Consequently, we did not estimate cost-effectiveness for any alternative subgroups. DVA is experienced across all social groups including all different socioeconomic, ethnic and geographical groups. The IRIS and IRIS+ programmes are designed for all social groups, therefore we did not consider distributional effects.

*Sensitivity analysis*



For peer review only - http://bmjopen.bmj.com/site/about/guidelines.xhtml

We undertook a probabilistic sensitivity analysis, based on 1000 simulations drawn from random samples from the probability distributions of all parameters. These 1000 simulations were plotted in a cost-effectiveness plane. The proportion of simulations with an incremental cost per QALY gained below the cost-effectiveness threshold was calculated for different threshold values, ranging from £0 to £50,000. The results were presented in a cost-effectiveness acceptability curve.

*Patient and public involvement*

Three patient and public involvement (PPI) groups (female survivors, male survivors and male perpetrators) were created and consulted throughout the lifetime of the research programme. PPI representatives were involved in the development of the IRIS+ intervention and the design of the research study.

## Results

*Base case*

The results of the cost-utility in the base case analysis are in Table 3. Over the ten-year time horizon, mean total costs per patient registered at general practices eligible to the IRIS+ pilot were £3,867. For the IRIS Programme, the mean cost per patient was £3,959. IRIS+ therefore could potentially save £92 per patient over a 10 year time horizon. Total QALYs per patient were also 0.003 higher in the intervention group (7.000) than in the control group (6.997). Because the intervention (IRIS+) was associated with lower costs and higher effectiveness the incremental cost per QALY gained was negative (dominating current practice, IRIS) and the incremental NMB was positive (£145).



For peer review only - http://bmjopen.bmj.com/site/about/guidelines.xhtml

**Table 3. Discounted base case and probabilistic results**

| Discounted base case results | | | |
|---|---|---|---|
| | **Costs** | **QALYs** | **Cost-effectiveness** |
| Intervention (IRIS+ programme) | £3,867 | 7.000 | |
| Control (IRIS programme) | £3,959 | 6.997 | |
| Difference (intervention vs. control) | -£92 | 0.003 | -ve (intervention dominates control) |
| Incremental NMB* | | | £145 |
| Probabilistic results | | | |
| | **Costs (95% CI)** | **QALYs (95% CI)** | **Cost-effectiveness (95% CI)** |
| Intervention (IRIS+ programme) | £107 to £16616 | 6.377 to 7.192 | |
| Control (IRIS programme) | £104 to £17343 | 6.377 to 7.197 | |
| Increment | £-1123 to £171 | -0.030 to 0.019 | |
| ICER | | | £-206828 to £277989 |

QALY = quality-adjusted life year. NMB = net monetary benefit. ICER= incremental cost effectiveness ratio. Costs are in 2019/20 UK£. Numbers may not sum due to rounding. *Measured at a willingness to pay for a QALY of £20 000.



For peer review only - http://bmjopen.bmj.com/site/about/guidelines.xhtml

*Sensitivity analysis*

Incremental costs and QALYs varied widely in probabilistic sensitivity analyses. The 95% confidence interval for incremental costs was -£1,123 to £171, for incremental QALYs it was -0.030 to 0.019 and for the ICER it was -£206,828 to £277,989 per QALY gained. Figure 2(a) shows a scatter plot of the incremental costs and incremental QALYs from the 1000 simulations. It shows how much uncertainty there is around these results. The IRIS+ programme was cost-effective in 55% of simulations when the cost-effectiveness threshold was £20, 000 (Figure 2(b)).

## Discussion

We found that the IRIS+ pilot is likely to be cost-effective or even cost-saving from a societal perspective in the UK with a willingness-to-pay threshold of £20,000 per gain in QALY, when compared to the IRIS Programme. There is considerable uncertainty surrounding these results, but the probability that IRIS+ was cost-effective was more than 50% at the cost-effectiveness threshold commonly used in the UK.

There are a number of strengths and limitations to this study. The main strength relates to this study drawing on newly collected data, reducing the need for using out-of-date previously published estimates. It, however, relies on a small number of observations (n=30 at baseline; n=16 at follow up), which could potentially be unreliable. The large uncertainty in our results reflects the small sample size. Nevertheless, as far as the authors are aware, this is the first study to assess the potential cost-effectiveness of a primary care intervention providing support to not just women, but also men and children experiencing DVA.



For peer review only - http://bmjopen.bmj.com/site/about/guidelines.xhtml

Comparing this prospective study to similar studies in the literature is challening. Most training and advocacy programmes evaluated to date, have focused on a subset of the population, such as women only, children only or men only. Including all groups is a key strength of the IRIS+ programme, as reported in the qualitative findings (34). Future research should attempt to replicate the intervention in a greater number of general practices across all parts of the UK to enable more robust data collection and larger sample sizes. The possibility of a randomised controlled trial comparing IRIS+ to IRIS could potentially address some of the uncertainties observed in the cost-effectiveness result of this study.

For peer review only

## References

1. Alliance VP. The public health approach. World Health Organisation (https://www who int/violenceprevention/approach/public_health/en/) Accessed. 2020;9.
2. Chandan JS, Taylor J, Bradbury-Jones C, Nirantharakumar K, Kane E, Bandyopadhyay S. COVID-19: a public health approach to manage domestic violence is needed. The Lancet Public Health. 2020;5(6):e309.
3. WHO. Violence against women: A global health problem of epidemic proportions Geneva: World Health Organization; 2013 [Available from: http://www.who.int/mediacentre/news/releases/2013/violence_against_women_20130620/en/.
4. Rakovec-Felser Z. Domestic violence and abuse in intimate relationship from public health perspective. Health psychology research. 2014;2(3).
5. Oliver R, Alexander, B., Roe, S., Wlasny, M. The economic and social costs of domestic abuse. London; 2019.
6. SafeLives. A Safe Fund: costing domestic abuse provision for the whole family: SafeLives; 2020 [Available from: https://safelives.org.uk/sites/default/files/A%20Safe%20Fund%20costing%20domestic%20abuse%20provision%20for%20the%20whole%20family%20in%20England%20and%20Wales_0.pdf.
7. Cleaver K, Maras P, Oram C, McCallum K. A review of UK based multi-agency approaches to early intervention in domestic abuse: Lessons to be learnt from existing evaluation studies. Aggression and violent behavior. 2019;46:140-55.
8. Hamberger LK, Larsen SE. Men's and women's experience of intimate partner violence: A review of ten years of comparative studies in clinical samples; Part I. Journal of Family Violence. 2015;30(6):699-717.
9. Walby S, Towers J, Francis B. Is violent crime increasing or decreasing? A new methodology to measure repeat attacks making visible the significance of gender and domestic relations. British Journal of Criminology. 2016;56(6):1203-34.
10. Feder G, Davies RA, Baird K, Dunne D, Eldridge S, Griffiths C, et al. Identification and Referral to Improve Safety (IRIS) of women experiencing domestic violence with a primary care training and support programme: a cluster randomised controlled trial. The Lancet. 2011;378(9805):1788-95.
11. Holt S, Buckley H, Whelan S. The impact of exposure to domestic violence on children and young people: A review of the literature. Child abuse & neglect. 2008;32(8):797-810.
12. McTavish JR, MacGregor JC, Wathen CN, MacMillan HL. Children's exposure to intimate partner violence: An overview. International review of psychiatry. 2016;28(5):504-18.
13. Coker AL, Davis KE, Arias I, Desai S, Sanderson M, Brandt HM, et al. Physical and mental health effects of intimate partner violence for men and women. American journal of preventive medicine. 2002;23(4):260-8.
14. ONS. Domestic abuse victim characteristics, England and Wales: year ending March 2020. Characteristics of victims of domestic abuse based on findings from the Crime Survey for England and Wales and police recorded crime. 2020 12th May 2022.
15. Hester M, Ferrari G, Jones SK, Williamson E, Bacchus LJ, Peters TJ, et al. Occurrence and impact of negative behaviour, including domestic violence and abuse, in men attending UK primary care health clinics: a cross-sectional survey. 2015;5(5).



For peer review only - http://bmjopen.bmj.com/site/about/guidelines.xhtml

16. Hester M, Williamson, E., Regan, L., Coulter, M., Chantler, K., Gangoli, G., Davenport, R., Green, L. . Exploring the service and support needs of male, lesbian, gay, bi-sexual and transgendered and black and other minority ethnic victims of domestic and sexual violence. Report prepared for Home Office SRG/06/017. Bristol; 2012.
17. Szilassy E, Caroline, C., Roy, J. Elizabeth, E., Gene, F. Feasibility of extending the IRIS domestic violence model 4th European Conference on Domestic Violence; Ljubljana: ECDV; 2021.
18. Roy J, Williamson E, Pitt K, Stanley N, Man MS, Feder G, et al. 'It felt like there was always someone there for us': Supporting children affected by domestic violence and abuse who are identified by general practice. Health & Social Care in the Community. 2022;30(1):165-74.
19. National Institute for Health and Care Excellence. Developing NICE guidelines: the manual. 2022 15th December 2022.
20. Pro Bono Economics. The economic cost from childhood exposure to severe domestic violence. 2018 15th December 2022.
21. Devine A, Spencer A, Eldridge S, Norman R, Feder G. Cost-effectiveness of Identification and Referral to Improve Safety (IRIS), a domestic violence training and support programme for primary care: a modelling study based on a randomised controlled trial. BMJ open. 2012;2(3).
22. Barbosa EC, Verhoef TI, Morris S, Solmi F, Johnson M, Sohal A, et al. Cost-effectiveness of a domestic violence and abuse training and support programme in primary care in the real world: updated modelling based on an MRC phase IV observational pragmatic implementation study. BMJ open. 2018;8(8):e021256.
23. Feder G, Davies RA, Baird K, Dunne D, Eldridge S, Griffiths C, et al. Identification and Referral to Improve Safety (IRIS) of women experiencing domestic violence with a primary care training and support programme: a cluster randomised controlled trial. Lancet (London, England). 2011;378(9805):1788-95.
24. Gilbert R, Widom CS, Browne K, Fergusson D, Webb E, Janson S. Burden and consequences of child maltreatment in high-income countries. The lancet. 2009;373(9657):68-81.
25. Taft AJ, Small R, Hegarty KL, Watson LF, Gold L, Lumley JA. Mothers' AdvocateS In the Community (MOSAIC)--non-professional mentor support to reduce intimate partner violence and depression in mothers: a cluster randomised trial in primary care. BMC public health. 2011;11:178.
26. ONS. Analysis of population estimates tool 2020 [Available from: https://www.ons.gov.uk/peoplepopulationandcommunity/populationandmigration/populationestimates/datasets/analysisofpopulationestimatestool.
27. Bonomi AE, Anderson ML, Reid RJ, Carrell D, Fishman PA, Rivara FP, et al. Intimate partner violence in older women. Gerontologist. 2007;47(1):34-41.
28. Rivas C, Ramsay J, Sadowski L, Davidson LL, Dunne D, Eldridge S, et al. Advocacy interventions to reduce or eliminate violence and promote the physical and psychosocial well-being of women who experience intimate partner abuse. status and date: New search for studies and content updated (no change to conclusions), published in. 2015(12).
29. Kind K, Hardman G, Macran S. UK Population norms for EQ-5D. University of York; 1999.
30. Brazier JE, Roberts J. The estimation of a preference-based measure of health from the SF-12. Medical care. 2004;42(9):851-9.



For peer review only - http://bmjopen.bmj.com/site/about/guidelines.xhtml

31. Stevens K. Valuation of the Child Health Utility 9D Index. Pharmacoeconomics. 2012;30(8):729-47.

32. Wittenberg E, Lichter EL, Ganz ML, McCloskey LA. Community preferences for health states associated with intimate partner violence. Medical care. 2006;44(8):738-44.

33. Oliver R, Alexander B, Roe S, Wlasny M. The economic and social costs of domestic abuse. Home Office (UK). 2019.

34. Szilassy E, Roy J, Williamson E, Pitt K, Man M-S, Feder G. Reaching everyone in general practice? Feasibility of an integrated domestic violence training and support intervention in primary care. BMC family practice. 2021;22(1):1-17.

For peer review only



For peer review only - http://bmjopen.bmj.com/site/about/guidelines.xhtml

**Figure 1. Health states and movement between health states in Markov model.**

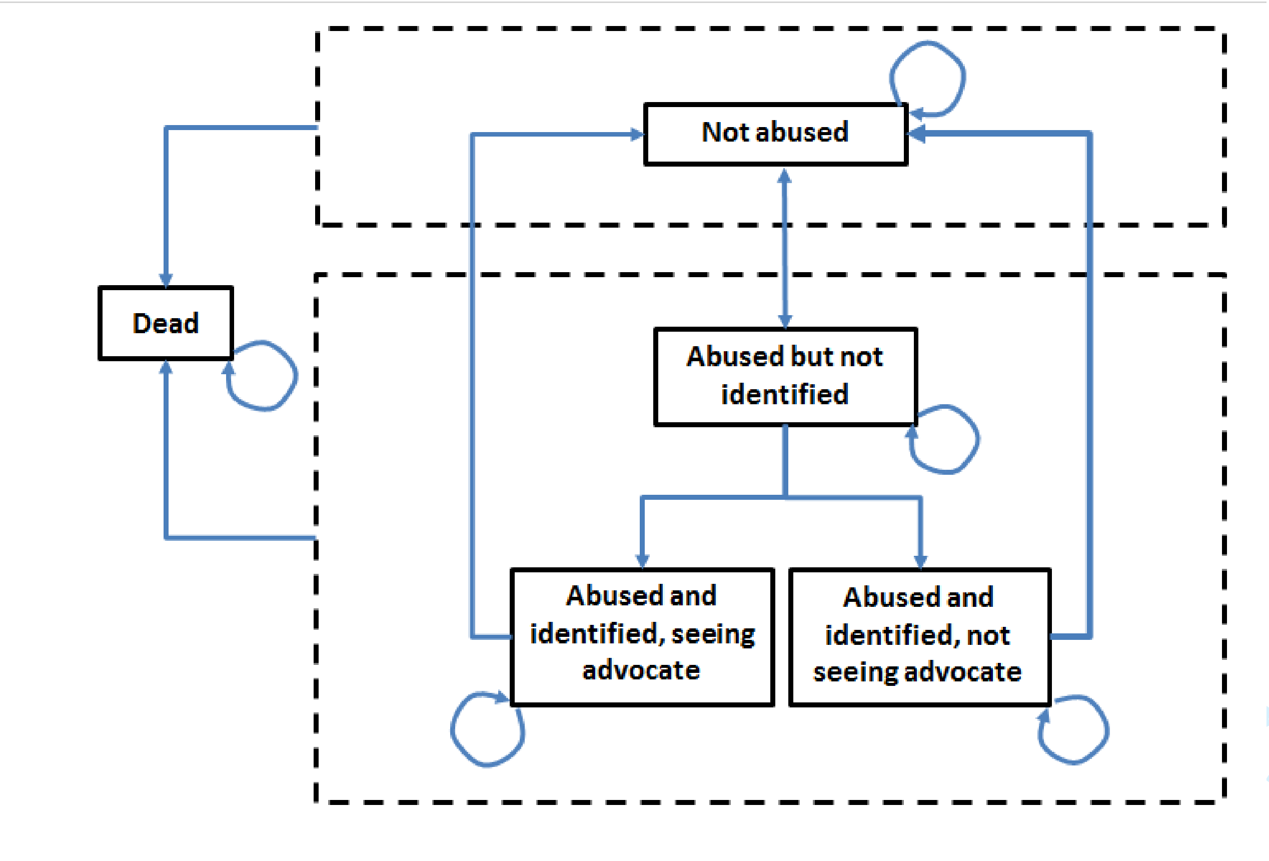


The model starts with all patients in either the ‘Not abused’ state or one of the states associated with abuse, based on the prevalence of DV (see text). Patients in the ‘Not abused’ state could stay in this state, move to ‘Abused but not identified’ or die from any cause. Once a patient is in the ‘Abused but not unidentified’ state, they could stay in that state, move back to ‘Not abused’, move to ‘Abused and identified, seeing advocate’ or ‘Abused and identified, not seeing advocate’ or die. Patients in the ‘Abused and identified’ states could stay in these states, move back to ‘Not abused’ or die.

Figure 2. Probabilistic sensitivity analysis.

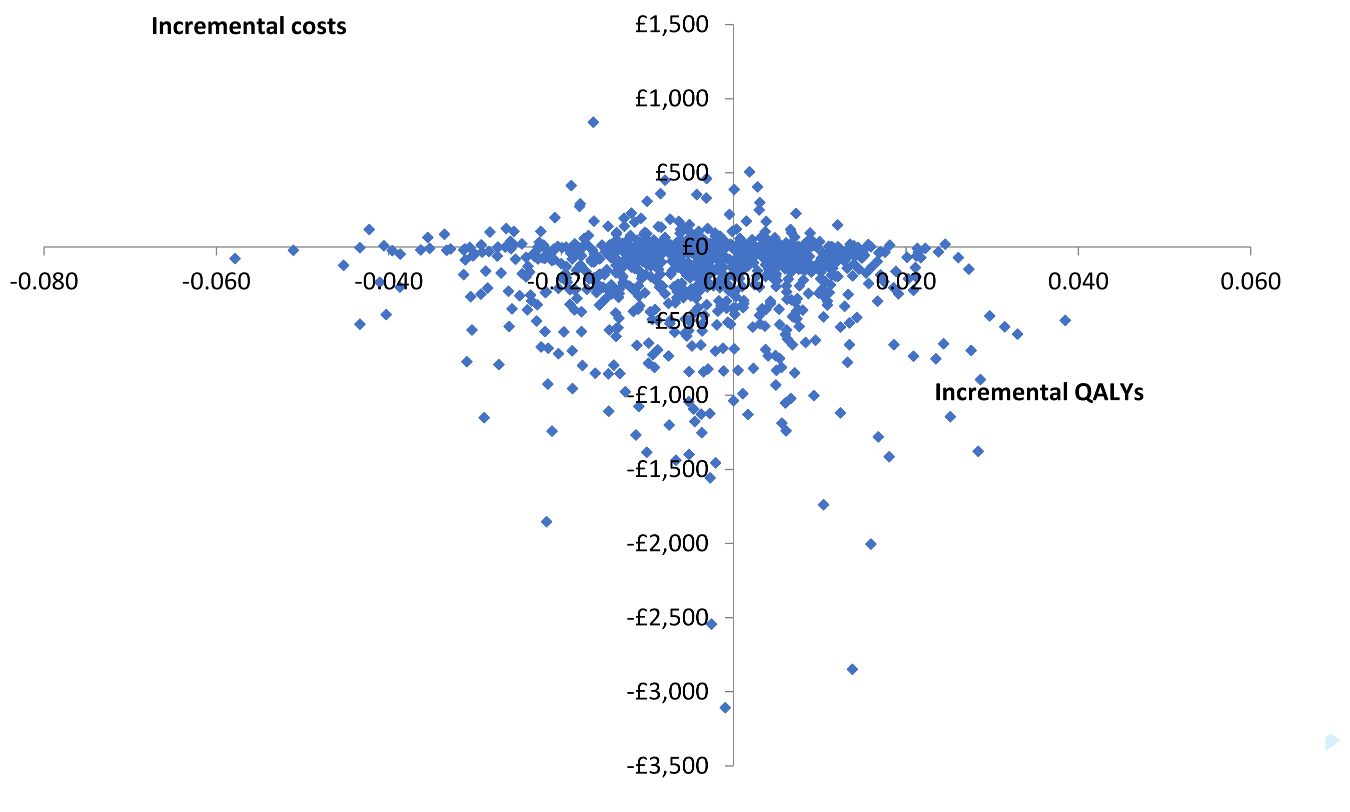


(a) Scatter plot of incremental costs and incremental QALYs from 1000 simulations

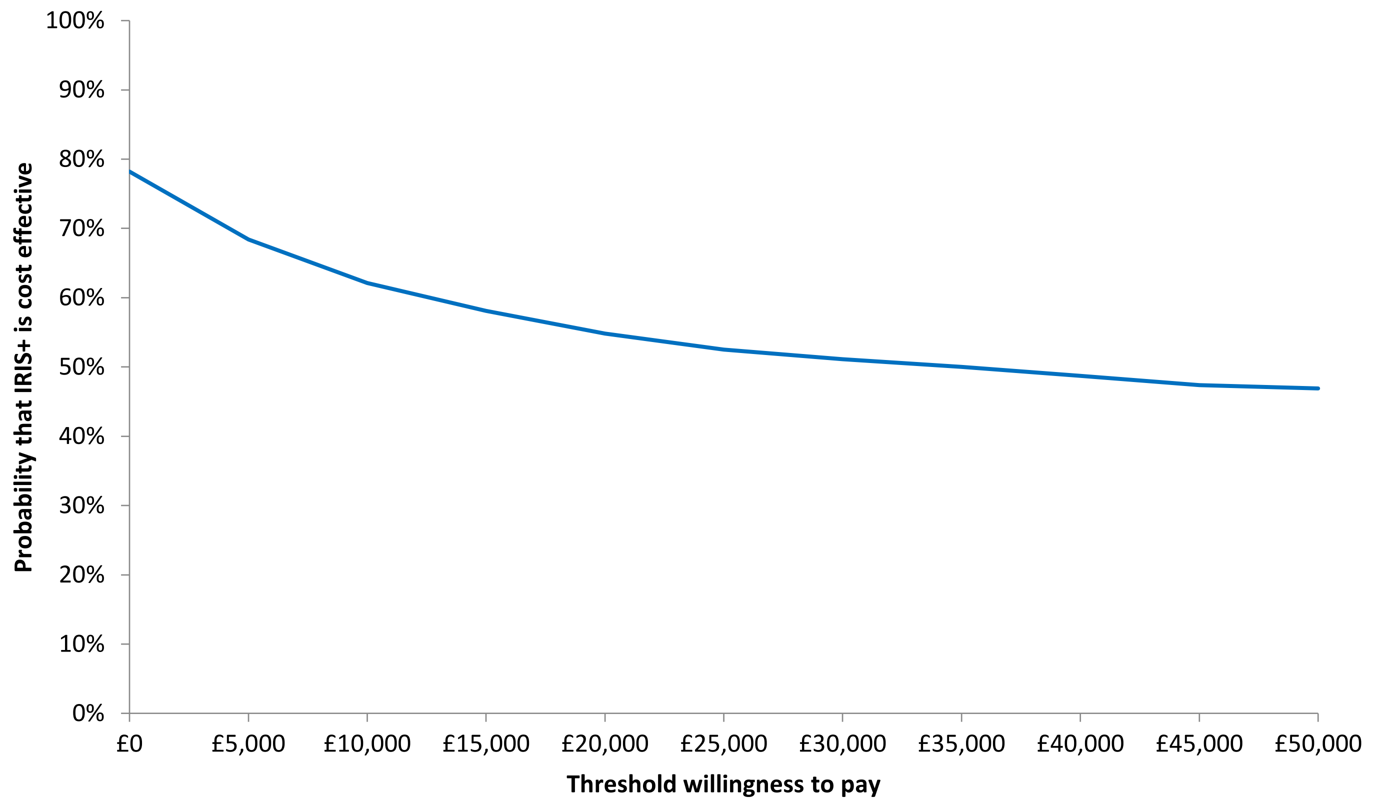


**(b) Cost-effectiveness acceptability curve showing the probability that the intervention (IRIS+) is cost-effective vs. control (IRIS) at different values of the maximum willingness to pay for a QALY**

QALY = quality-adjusted life year. Costs are in 2019/20 UK£

**Primary care system-level training and support programme for the secondary prevention of domestic violence and abuse: a cost-effectiveness feasibility model**

Madeleine Cochrane, Eszter Szilassy, Caroline Coope, Elizabeth Emsley, Medina Johnson, Gene Feder and Estela Capelas Barbosa

For peer review only

**Table S1. Adult's model input parameters: probabilities; utilities; and, costs.**

| Parameter | Base case value | Lower limit | Upper limit | Distribution | Source |
|---|---|---|---|---|---|
| ***Probabilities*** | | | | | |
| Prevalence of DVA in adults (males and females) – aged 16 to 65 | 0.055 | 0.036 | 0.073 | Beta | ONS, 2020b |
| *Starting distribution for patients who are abused* | | | | | |
| Abused and identified, seeing advocate | 0.003 | 0 | 0.0066 | Uniform | * |
| Abused and identified, not seeing advocate | 0.033 | 0 | 0.0660 | Uniform | * |
| Abused but not identified | 0.964 | - | - | Uniform | Complement |
| *Transition probabilities* | | | | | |
| Not abused to Abused but not identified | 0.0037 | 0.0004 | 0.0106 | Dirichlet | * |
| Not abused to Dead | 0.0052 | 0.0027 | 0.0087 | Dirichlet | ONS, 2020b |
| Stay in Not abused | 0.9911 | - | - | Dirichlet | Complement |
| Abused but not identified to Not abused (control) | 0.0500 | 0.0412 | 0.0596 | Dirichlet | * |
| Abused but not identified to Abused and identified, not seeing advocate (control) | 0.0091 | 0.0055 | 0.0135 | Dirichlet | IRIS data |

For peer review only

| | | | | | |
|---|---|---|---|---|---|
| Abused but not identified to Abused and identified, seeing advocate (control) | 0.0226 | 0.0168 | 0.0293 | Dirichlet | IRIS data |
| Abused but not identified to Dead (control) | 0.0055 | 0.0029 | 0.0091 | Dirichlet | ONS, 2020b |
| Stay in Abused but not identified (control) | 0.9131 | - | - | Dirichlet | Complement |
| Abused but not identified to Not abused (intervention) | 0.0500 | 0.0412 | 0.0596 | Dirichlet | * |
| Abused but not identified to Abused and identified, not seeing advocate (intervention) | 0.0374 | 0.0298 | 0.0458 | Dirichlet | IRIS+ data |
| Abused but not identified to Abused and identified, seeing advocate (intervention) | 0.0312 | 0.0243 | 0.0390 | Dirichlet | IRIS+ data |
| Abused but not identified to Dead (intervention) | 0.0055 | 0.0029 | 0.0091 | Dirichlet | ONS, 2020B |
| Stay in Abused but not identified (intervention) | 0.8762 | - | - | Dirichlet | Complement |
| Abused and identified, seeing advocate to Not abused | 0.1408 | 0.0707 | 0.2301 | Dirichlet | (Taft et al., 2011) |
| Abused and identified, seeing advocate to Dead | 0.0052 | 0.0000 | 0.0299 | Dirichlet | ONS, 2020b |
| Stay in Abused and identified, seeing advocate | 0.854 | - | - | Dirichlet | Complement |
| Abused and identified, not seeing advocate to Not abused | 0.0781 | 0.0136 | 0.1912 | Dirichlet | (Taft et al., 2011) |
| Abused and identified, not seeing advocate to Dead | 0.0052 | 0.0000 | 0.0424 | Dirichlet | ONS, 2020b |

For peer review only

| | | | | | |
|---|---|---|---|---|---|
| Stay in Abused and identified, not seeing advocate | 0.9167 | - | - | Dirichlet | Complement |
| ***Utilities*** | | | | | |
| Not abused (adults) | 0.850 | 0.840 | 0.860 | Beta | (Kind et al., 1999) |
| Abused but not identified (women) | 0.656 | 0.522 | 0.749 | Beta | Assumption |
| Abused but not identified (men) | 0.626 | 0.500 | 0.744 | Beta | Assumption |
| Abused and identified, seeing advocate (women) | 0.659 | 0.518 | 0.782 | Beta | IRIS+ data |
| Abused and identified, seeing advocate (men) | 0.701 | 0.555 | 0.828 | Beta | IRIS+ data |
| Abused and identified, not seeing advocate (women) | 0.656 | 0.522 | 0.749 | Beta | IRIS+ data |
| Abused and identified, not seeing advocate (men) | 0.626 | 0.500 | 0.744 | Beta | IRIS+ data |
| ***Costs (2019/20£)*** | | | | | |
| Costs of the intervention, per patient exposed to DV, per 6 months | £0.74 | £0.02 | £2.73 | Gamma | IRIS+ budget |
| Cost of onward referral, once | £658 | £11 | £1908 | Gamma | IRIS+ data; IRIS data |
| Cost of Abused but not identified (adults) | £4858 | £123 | £17919 | Gamma | (Oliver et al., 2019) |
| Weighted costs Abused and identified, seeing advocate | 1 | 0.75 | 1.25 | Gamma | Assumption |
| Weighted costs Abused and identified, not seeing advocate | 1 | 0.9 | 1.1 | Gamma | Assumption |

Costs are in 2019/20 UK£.
* Internal calculation based on model calibration.

For peer review only

± Excludes the cost of harms, which in this modelled are measured as benefit

**Table S2. Children's model input parameters: probabilities; utilities; and, costs.**

| Parameter | Base case value | Lower limit | Upper limit | Distribution | Source |
|---|---|---|---|---|---|
| ***Probabilities*** | | | | | |
| Prevalence of children exposed to DVA | 0.080 | 0.040 | 0.140 | Beta | (Gilbert et al., 2009) |
| *Starting distribution for patients who are abused* | | | | | |
| Abused and identified, seeing advocate | 0.003 | 0 | 0.0066 | Uniform | * |
| Abused and identified, not seeing advocate | 0.033 | 0 | 0.0660 | Uniform | * |
| Abused but not identified | 0.964 | - | - | Uniform | Complement |
| *Transition probabilities* | | | | | |
| Not abused to Abused but not identified | 0.0037 | 0.0004 | 0.0106 | Dirichlet | * |
| Not abused to Dead | 0.0052 | 0.0027 | 0.0087 | Dirichlet | ONS, 2020b |
| Stay in Not abused | 0.9911 | - | - | Dirichlet | Complement |
| Abused but not identified to Not abused (control) | 0.0500 | 0.0412 | 0.0596 | Dirichlet | * |

| | | | | | |
|---|---|---|---|---|---|
| Abused but not identified to Abused and identified, not seeing advocate (control) | 0.0091 | 0.0055 | 0.0135 | Dirichlet | IRIS data |
| Abused but not identified to Abused and identified, seeing advocate (control) | 0.0226 | 0.0168 | 0.0293 | Dirichlet | IRIS data |
| Abused but not identified to Dead (control) | 0.0055 | 0.0029 | 0.0091 | Dirichlet | ONS, 2020b |
| Stay in Abused but not identified (control) | 0.9131 | - | - | Dirichlet | Complement |
| Abused but not identified to Not abused (intervention) | 0.0500 | 0.0412 | 0.0596 | Dirichlet | * |
| Abused but not identified to Abused and identified, not seeing advocate (intervention) | 0.0374 | 0.0298 | 0.0458 | Dirichlet | IRIS+ data |
| Abused but not identified to Abused and identified, seeing advocate (intervention) | 0.0312 | 0.0243 | 0.0390 | Dirichlet | IRIS+ data |
| Abused but not identified to Dead (intervention) | 0.0055 | 0.0029 | 0.0091 | Dirichlet | ONS, 2020b |
| Stay in Abused but not identified (intervention) | 0.8762 | - | - | Dirichlet | Complement |
| Abused and identified, seeing advocate to Not abused | 0.1408 | 0.0707 | 0.2301 | Dirichlet | (Taft et al., 2011) |
| Abused and identified, seeing advocate to Dead | 0.0052 | 0.0000 | 0.0299 | Dirichlet | ONS, 2020b |
| Stay in Abused and identified, seeing advocate | 0.854 | - | - | Dirichlet | Complement |

| | | | | | |
|---|---|---|---|---|---|
| Abused and identified, not seeing advocate to Not abused | 0.0781 | 0.0136 | 0.1912 | Dirichlet | (Taft et al., 2011) |
| Abused and identified, not seeing advocate to Dead | 0.0052 | 0.0000 | 0.0424 | Dirichlet | ONS, 2020b |
| Stay in Abused and identified, not seeing advocate | 0.9167 | - | - | Dirichlet | Complement |
| ***Utilities*** | | | | | |
| Not abused (children) | 0.950 | 0.940 | 0.959 | Beta | (Kind et al., 1999) |
| Abused but not identified (children) | 0.801 | 0.623 | 0.932 | Beta | Assumption |
| Abused and identified, seeing advocate (children) | 0.804 | 0.625 | 0.935 | Beta | IRIS+ data |
| Abused and identified, not seeing advocate (children) | 0.801 | 0.623 | 0.932 | Beta | IRIS+ data |
| ***Costs (2019/20£)*** | | | | | |
| Costs of the intervention, per patient exposed to DV, per 6 months | £0.74 | £0.02 | £2.73 | Gamma | IRIS+ budget |
| Cost of onward referral, once | £658 | £11 | £1908 | Gamma | IRIS+ data; IRIS data |
| Cost of Abused but not identified (children) | £1950 | £1000 | £2500 | Gamma | (Pro Bono Economics, 2018) |
| Weighted costs Abused and identified, seeing advocate | 1 | 0.75 | 1.25 | Gamma | Assumption |
| Weighted costs Abused and identified, not seeing advocate | 1 | 0.9 | 1.1 | Gamma | Assumption |

Costs are in 2019/20 UK£.
* Internal calculation based on model calibration.

For peer review only

± Excludes the cost of harms, which in this modelled are measured as benefit

For peer review only

# CHEERS 2022 Checklist

| Topic | No. | Item | Location where item is reported |
|---|---|---|---|
| **Title** | | | |
| **Title** | 1 | Identify the study as an economic evaluation and specify the interventions being compared. | Title, Page 1 |
| **Abstract** | | | |
| **Abstract** | 2 | Provide a structured summary that highlights context, key methods, results, and alternative analyses. | Abstract, Page 1 |
| **Introduction** | | | |
| **Background and objectives** | 3 | Give the context for the study, the study question, and its practical relevance for decision making in policy or practice. | Background, Page 4 |
| **Methods** | | | |
| **Health economic analysis plan** | 4 | Indicate whether a health economic analysis plan was developed and where available. | Methods, Page 5 |
| **Study population** | 5 | Describe characteristics of the study population (such as age range, demographics, socioeconomic, or clinical characteristics). | Methods, Page 7 |
| **Setting and location** | 6 | Provide relevant contextual information that may influence findings. | Methods, Page 6 |
| **Comparators** | 7 | Describe the interventions or strategies being compared and why chosen. | Methods, Page 6-7 |
| **Perspective** | 8 | State the perspective(s) adopted by the study and why chosen. | Methods, Page 5 |
| **Time horizon** | 9 | State the time horizon for the study and why appropriate. | Methods, Page 5 |
| **Discount rate** | 10 | Report the discount rate(s) and reason chosen. | Methods, Page 6 |
| **Selection of outcomes** | 11 | Describe what outcomes were used as the measure(s) of benefit(s) and harm(s). | Methods, Page 5 |
| **Measurement of outcomes** | 12 | Describe how outcomes used to capture benefit(s) and harm(s) were measured. | Methods, Page 10 |
| **Valuation of outcomes** | 13 | Describe the population and methods used to measure and value outcomes. | Methods, Page 10 |



For peer review only - http://bmjopen.bmj.com/site/about/guidelines.xhtml

| Topic | No. | Item | Location where item is reported |
|---|---|---|---|
| **Measurement and valuation of resources and costs** | 14 | Describe how costs were valued. | Methods, Page 11 |
| **Currency, price date, and conversion** | 15 | Report the dates of the estimated resource quantities and unit costs, plus the currency and year of conversion. | Methods, Page 5 |
| **Rationale and description of model** | 16 | If modelling is used, describe in detail and why used. Report if the model is publicly available and where it can be accessed. | Methods, Page 6 |
| **Analytics and assumptions** | 17 | Describe any methods for analysing or statistically transforming data, any extrapolation methods, and approaches for validating any model used. | Methods, Table 1 and Page 6-11 |
| **Characterising heterogeneity** | 18 | Describe any methods used for estimating how the results of the study vary for subgroups. | Methods, Page 12 |
| **Characterising distributional effects** | 19 | Describe how impacts are distributed across different individuals or adjustments made to reflect priority populations. | Methods, Page 12 |
| **Characterising uncertainty** | 20 | Describe methods to characterise any sources of uncertainty in the analysis. | Methods, Page 12 |
| **Approach to engagement with patients and others affected by the study** | 21 | Describe any approaches to engage patients or service recipients, the general public, communities, or stakeholders (such as clinicians or payers) in the design of the study. | Methods, Page 6 |
| Results | | | |
| **Study parameters** | 22 | Report all analytic inputs (such as values, ranges, references) including uncertainty or distributional assumptions. | Results, Table 1 |
| **Summary of main results** | 23 | Report the mean values for the main categories of costs and outcomes of interest and summarise them in the most appropriate overall measure. | Results, Table 2 |
| **Effect of uncertainty** | 24 | Describe how uncertainty about analytic judgments, inputs, or projections affect findings. Report the effect of choice of discount rate and time horizon, if applicable. | Results, Figure 2 |
| **Effect of engagement with patients and others affected by the study** | 25 | Report on any difference patient/service recipient, general public, community, or stakeholder involvement made to the approach or findings of the study | Not applicable as model was based on previous study. |
| **Discussion** | | | |
| **Study findings, limitations, generalisability, and current knowledge** | 26 | Report key findings, limitations, ethical or equity considerations not captured, and how these could affect patients, policy, or practice. | Discussion, Page 13-14 |



For peer review only - http://bmjopen.bmj.com/site/about/guidelines.xhtml

| Topic | No. | Item | Location where item is reported |
|---|---|---|---|
| Other relevant information | | | |
| **Source of funding** | 27 | Describe how the study was funded and any role of the funder in the identification, design, conduct, and reporting of the analysis | Funding statement and acknowledgments, Page 15 |
| **Conflicts of interest** | 28 | Report authors conflicts of interest according to journal or International Committee of Medical Journal Editors requirements. | Competing interests, Page 15 |

*From:* Husereau D, Drummond M, Augustovski F, et al. Consolidated Health Economic Evaluation Reporting Standards 2022 (CHEERS 2022) Explanation and Elaboration: A Report of the ISPOR CHEERS II Good Practices Task Force. Value Health 2022;25. doi:10.1016/j.jval.2021.10.008

For peer review only

BMJ Open

# Primary care system-level training and support programme for the secondary prevention of domestic violence and abuse: a cost-effectiveness feasibility model

| | |
|---|---|
| Journal: | *BMJ Open* |
| Manuscript ID | bmjopen-2022-071300.R1 |
| Article Type: | Original research |
| Date Submitted by the Author: | 25-May-2023 |
| Complete List of Authors: | Cochrane, Madeleine; University of Bristol,<br>Szilassy, Eszter; University of Bristol<br>Coope, Caroline; University of Bristol<br>Emsley, Elizabeth; University of Bristol<br>Johnson, Medina; IRISi<br>Feder, Gene; University of Bristol<br>Barbosa, Estela; City University of London |
| <b>Primary Subject Heading</b>: | Health economics |
| Secondary Subject Heading: | Public health |
| Keywords: | PRIMARY CARE, HEALTH ECONOMICS, PUBLIC HEALTH |
| | |

SCHOLARONE™
Manuscripts

*I, the Submitting Author has the right to grant and does grant on behalf of all authors of the Work (as defined in the below author licence), an exclusive licence and/or a non-exclusive licence for contributions from authors who are: i) UK Crown employees; ii) where BMJ has agreed a CC-BY licence shall apply, and/or iii) in accordance with the terms applicable for US Federal Government officers or employees acting as part of their official duties; on a worldwide, perpetual, irrevocable, royalty-free basis to BMJ Publishing Group Ltd ("BMJ") its licensees and where the relevant Journal is co-owned by BMJ to the co-owners of the Journal, to publish the Work in this journal and any other BMJ products and to exploit all rights, as set out in our licence.*

*The Submitting Author accepts and understands that any supply made under these terms is made by BMJ to the Submitting Author unless you are acting as an employee on behalf of your employer or a postgraduate student of an affiliated institution which is paying any applicable article publishing charge ("APC") for Open Access articles. Where the Submitting Author wishes to make the Work available on an Open Access basis (and intends to pay the relevant APC), the terms of reuse of such Open Access shall be governed by a Creative Commons licence – details of these licences and which Creative Commons licence will apply to this Work are set out in our licence referred to above.*

*Other than as permitted in any relevant BMJ Author's Self Archiving Policies, I confirm this Work has not been accepted for publication elsewhere, is not being considered for publication elsewhere and does not duplicate material already published. I confirm all authors consent to publication of this Work and authorise the granting of this licence.*

For peer review only

**Title Page**

# Primary care system-level training and support programme for the secondary prevention of domestic violence and abuse: a cost-effectiveness feasibility model

Madeleine Cochrane, Eszter Szilassy, Caroline Coope, Elizabeth Emsley, Medina Johnson, Gene Feder and Estela Capelas Barbosa

**Correspondence**

*Corresponding author:*

Madeleine Cochrane: madeleine.cochrane@bristol.ac.uk

Population Health Sciences, Bristol Medical School, University of Bristol, 1-5 Whiteladies Road, Bristol, BS8 1NU, UK. Tel: 0117 428 3116

*Co-authors:*

Eszter Szilassy: eszter.szilassy@bristol.ac.uk

Caroline Coope: caroline.coope@bristol.ac.uk

Elizabeth Emsley: elizabeth.emsley@bristol.ac.uk

Medina Johnson: medina.johnson@irisi.org

Gene Feder: gene.feder@bristol.ac.uk

Estela Capelas Barbosa: estela.barbosa@city.ac.uk

Population Health Sciences, Bristol Medical School, University of Bristol, 1-5 Whiteladies Road, Bristol, BS8 1NU, UK. Eszter Szilassy, Caroline Coope, Elizabeth Emsley, Gene Feder.

City University of London, Northampton Square, London, EC1V 0HB. UK. Estela Capelas Barbosa



For peer review only

For peer review only - http://bmjopen.bmj.com/site/about/guidelines.xhtml

IRISi, London, UK. Medina Johnson.

**Keywords**

Domestic violence, training programme, general practice, primary care, cost-effectiveness.

For peer review only



For peer review only - http://bmjopen.bmj.com/site/about/guidelines.xhtml

## Abstract

**Objectives:** To evaluate the prospective cost-effectiveness of the Identification and Referral to Improve Safety plus (IRIS+) intervention compared to usual care using feasibility data derived from seven UK GP practice sites.

**Method:** A cost-utility analysis was conducted to assess the potential cost-effectiveness of IRIS+, an enhanced model of the UK's usual care. IRIS+ assisted primary care staff in identifying, documenting and referring not only women, but also men and children who may have experienced domestic violence/ abuse as victims, perpetrators, or both. A perpetrator group programme was linked to the IRIS+ intervention via a referral pathway or signposting, and not part of the intervention per se. A Markov model was constructed from a societal perspective to estimate mean incremental costs and quality-adjusted life-years (QALYs) of IRIS+ compared to usual care over a 10-year time horizon.

**Results:** The IRIS+ intervention saved £92 per patient and produced QALY gains of 0.003. The incremental net monetary benefit was positive (£145) and the IRIS+ intervention was cost-effective in 55% of simulations at a cost-effectiveness threshold of £20,000 per QALY.

**Conclusion:** The IRIS+ intervention is likely to be cost-effective or even cost-saving from a societal perspective in the UK, though there are large uncertainties, reflected in the confidence intervals.


For peer review only

**Strengths and Limitations**

- To the authors knowledge this is the first study to assess the potential cost-effectiveness of a primary care intervention providing support to all women, men and their children experiencing domestic violence/ abuse
- The study draws on the structure of a previous domestic violence/ abuse model which has been published in peer-reviewed journals
- The study relies on newly collected data, reducing the need for using out-of-date previously published estimates
- The small number of newly collected data means our results may not be representative of the wider UK population



For peer review only - http://bmjopen.bmj.com/site/about/guidelines.xhtml

**Background**

Domestic violence/ abuse (DVA) is a public health challenge, affecting approximately 9 million adults and 2 million children in the UK (1-4). The societal cost of DVA was estimated by the UK Home Office to be £66bn in 2017, not including costs to children. Safe Lives, a UK-wide DVA charity, highlighted the need for an initial £2.2bn of public investment per annum to cover domestic abuse services for the whole family – adult, teen and child victims, and perpetrators (5, 6). Public Health England identified primary care as a key location for interventions to prevent DVA and improve health outcomes for adults and children. Early intervention in DVA, for example, in the primary care setting, reduces the overall public service burden of abuse and can reduce escalation of violence (7).

DVA interventions to date have prioritised women, who are disproportionately affected in prevalence and severity of DVA, compared with other groups (8, 9). Identifying female survivors in primary care and referring to specialist support is effective and cost-effective through the provision of DVA training linked with a direct pathway to local DVA support (10). The leading service model in the UK's National Health Service (NHS) primary care setting is IRIS (Identification and Referral to Improve Safety), a widely commissioned evidence-based DVA training and advocacy support programme for female survivors.

While there is growing success in identifying women affected by DVA, male survivors and children/ young people (CYP) are rarely identified in primary care and referred for specialist support. This neglects the mental and physical health impact across the life-course for CYP who experience or witness DVA (11, 12) and the significant mental health impact on men exposed to DVA (13-16). IRIS plus (IRIS+) was an enhanced model of the existing IRIS programme and was piloted in NHS primary care GP sites, three sites in England and four sites in Wales. IRIS+ assisted GP practice staff in identifying, documenting and referring not only

women, but also men and children who may have experienced DVA as victims, perpetrators, or both. The IRIS+ pilot study showed feasibility and acceptability of the intervention to clinicians and those affected by DVA (17, 18). The aim of this study was to evaluate the prospective cost-effectiveness of the IRIS+ intervention when compared to usual care (the IRIS intervention). This study addresses a gap in the literature around the possible cost-effectiveness of interventions targeting men and children as well as women experiencing DVA.

## Methods

*Overview of economic evaluation*

This study was a model-based cost–utility analysis, comparing the IRIS+ intervention to usual care (the IRIS intervention). An unpublished health economic analysis plan (HEAP) was developed prospectively to guide the economic evaluation. The outcome measure was quality-adjusted life years (QALYs), which is the recommended outcome for economic evaluations in the UK (19). As many of the costs of DVA are borne outside the health system, the analysis was undertaken from a UK societal perspective which in this study we define as the costs associated with implementing the intervention, downstream multi-sector costs associated with DVA, as well as productivity costs. Costs relating to DVA perpetration were included in the cost of onward referral, given that a perpetrator programme was linked to IRIS+ via an onward referral pathway or signposting. Costs were calculated in 2019/20 UK£, as most of the IRIS+ intervention took place in those years. Costs and benefits were calculated over a 10-year time horizon. This was considered appropriate because the occurrence of new cases and transition probabilities were assumed to remain constant over time and therefore the impact of a longer time horizon would be small. While this is likely to be the case for adults, we acknowledge that the time horizon for children may be longer (20). This means we opted for a conservative estimate of the cost-effectiveness of the intervention as far as children are concerned. Future



For peer review only - http://bmjopen.bmj.com/site/about/guidelines.xhtml

costs and outcomes were discounted at an annual rate of 3.5% as recommended in the UK guidelines for conducting economic evaluations (19) .

*Model structure*

We developed a Markov model based on the previous analysis of the cost-effectiveness of the usual care intervention (IRIS) (21, 22). The model has five health states (see Figure 1 for details) and the cycle length was six months, which reflects the average length of support received from advocacy services following referral. The cycle length of six months also reflects the maximum time of support available for identified patients. Other than death, which is an absorbing state, men, women and children can transition between states in half-yearly cycles. The states were 'No abuse', 'Abuse not identified', 'Abuse identified and seeing advocate', 'Abuse identified, not seeing advocate' and 'Dead' (Figure 1). A hypothetical cohort of 10,000 people was simulated moving between the states (Figure 1). We used the Census figures to estimate the proportion of adult men, women and children within this hypothetical cohort (14). The model was built and run using Excel VBA.

*Interventions*

*The IRIS intervention(usual care arm)*

The IRIS intervention is a multi-component intervention which has been described elsewhere (21, 23). In short, it is delivered in UK NHS primary care GP sites and consists of multidisciplinary training sessions, targeted at the clinical team and some general practice reception staff. The training sessions were designed to address barriers to improving the response of clinicians to women experiencing abuse through improved identification, support and referral to specialist agencies. Clinicians are trained to have a low threshold for asking about DVA. Training incorporates case studies and practice in asking about violence and



For peer review only - http://bmjopen.bmj.com/site/about/guidelines.xhtml

responding appropriately. They are delivered by an advocate educator from collaborating specialist support services. The advocate educator is central to the IRIS intervention, combining a training and support role to the practices with provision of advocacy to women referred. Ongoing support to clinicians and reception staff in the practices is provided by the advocate educator.

*The IRIS+ intervention (intervention arm)*

The IRIS+ intervention builds on the IRIS model, but in addition provides a service for men and children. Similar to IRIS, it consists of a multi-component intervention, including multidisciplinary training for clinicians and general practice staff. IRIS+ provides a simple pathway of referrals to specialist support services for women, men and their children who experience (survivors and perpetrators) DVA. In IRIS+, as well as the advocate educator, there is a dedicated children's worker. Jointly they support any referral made by clinicians, regardless of gender or age. While perpetrators could have been identified by the IRIS+ intervention, the perpetrator group programme was linked to the IRIS+ intervention via a referral pathway or signposting, meaning it was not part of the intervention per se. Perpetrators could also self-refer into the perpetrator program.

*Comparisons between IRIS+ (intervention arm) and IRIS (usual care arm)*

Given that this study was a pilot, we did not recruit practices into the usual care arm (IRIS). In fact, the recruitment for IRIS+ included seven practices, three non-IRIS trained practices that had not previously received IRIS or practice-based DVA interventions, and four IRIS-trained practices that had previously received IRIS training. The comparison between IRIS (usual care) and IRIS+ used estimated parameters based on the same areas, given both IRIS and IRIS+ programmes were available for this subset of practices.



For peer review only - http://bmjopen.bmj.com/site/about/guidelines.xhtml

*Parameters*

Whenever possible, we used data collected in the pilot to estimate transition probabilities, utilities and costs required for the Markov model. Where this was not possible probabilities were obtained from published sources. Table 1 shows the source of data for each relevant parameter. Tables S1 and S2 report the same parameters however, they are reported in separate tables for adults and children, respectively.



For peer review only - http://bmjopen.bmj.com/site/about/guidelines.xhtml

**Table 1. Model input parameters: probabilities**

| Parameter | Base case value | Lower limit | Upper limit | Distribution | Source |
|---|---|---|---|---|---|
| ***Probabilities*** | | | | | |
| Proportion of patients experiencing abuse | 0.055 | 0.038 | 0.106 | Beta | **adjusted estimate |
| Prevalence of DVA in adults (males and females) – aged 16 to 65 | 0.055 | 0.036 | 0.073 | Beta | (14) |
| Prevalence of children exposed to DVA | 0.080 | 0.040 | 0.140 | Beta | (24) |
| *Starting distribution for those experiencing abuse* | | | | | |
| Abused and identified, seeing advocate | 0.003 | 0 | 0.0066 | Uniform | * |
| Abused and identified, not seeing advocate | 0.033 | 0 | 0.0660 | Uniform | * |
| Abused but not identified | 0.964 | - | - | Uniform | Complement |
| *Transition probabilities* | | | | | |
| Not abused to Abused but not identified | 0.0037 | 0.0004 | 0.0106 | Dirichlet | * |
| Not abused to Dead | 0.0052 | 0.0027 | 0.0087 | Dirichlet | (14) |
| Stay in Not abused | 0.9911 | - | - | Dirichlet | Complement |
| Abused but not identified to Not abused (control) | 0.0500 | 0.0412 | 0.0596 | Dirichlet | * |



For peer review only - http://bmjopen.bmj.com/site/about/guidelines.xhtml

| | | | | | |
|---|---|---|---|---|---|
| Abused but not identified to Abused and identified, not seeing advocate (control) | 0.0091 | 0.0055 | 0.0135 | Dirichlet | IRIS data |
| Abused but not identified to Abused and identified, seeing advocate (control) | 0.0226 | 0.0168 | 0.0293 | Dirichlet | IRIS data |
| Abused but not identified to Dead (control) | 0.0055 | 0.0029 | 0.0091 | Dirichlet | (14) |
| Stay in Abused but not identified (control) | 0.9131 | - | - | Dirichlet | Complement |
| Abused but not identified to Not abused (intervention) | 0.0500 | 0.0412 | 0.0596 | Dirichlet | * |
| Abused but not identified to Abused and identified, not seeing advocate (intervention) | 0.0374 | 0.0298 | 0.0458 | Dirichlet | IRIS+ data |
| Abused but not identified to Abused and identified, seeing advocate (intervention) | 0.0312 | 0.0243 | 0.0390 | Dirichlet | IRIS+ data |
| Abused but not identified to Dead (intervention) | 0.0055 | 0.0029 | 0.0091 | Dirichlet | (14) |
| Stay in Abused but not identified (intervention) | 0.8762 | - | - | Dirichlet | Complement |
| Abused and identified, seeing advocate to Not abused | 0.1408 | 0.0707 | 0.2301 | Dirichlet | (25) |
| Abused and identified, seeing advocate to Dead | 0.0052 | 0.0000 | 0.0299 | Dirichlet | (14) |
| Stay in Abused and identified, seeing advocate | 0.854 | - | - | Dirichlet | Complement |



For peer review only - http://bmjopen.bmj.com/site/about/guidelines.xhtml

| | | | | | |
|---|---|---|---|---|---|
| Abused and identified, not seeing advocate to Not abused | 0.0781 | 0.0136 | 0.1912 | Dirichlet | (25) |
| Abused and identified, not seeing advocate to Dead | 0.0052 | 0.0000 | 0.0424 | Dirichlet | (14) |
| Stay in Abused and identified, not seeing advocate | 0.9167 | - | - | Dirichlet | Complement |

* Internal calculation based on model calibration

For peer review only



For peer review only - http://bmjopen.bmj.com/site/about/guidelines.xhtml

*Prevalence of domestic abuse*

The proportion of adults aged 16 years or older experiencing abuse was estimated from the Crime Survey for England and Wales (CSEW) (14). There was a subsequent published estimate, but due to anti-contagion measures relating to the COVID-19 pandemic, the survey had to be moved to telephone survey, preventing some of the collection of relevant data on domestic violence. In 2018/19, 5.5% of adults experienced some form of domestic violence according to the CSEW (14). Since IRIS+ also provides support services for children, we relied on the published estimate of 8% of children experiencing some form of DVA in the past 12 months (24). Children represent 20% of the UK population (26). To extrapolate beyond age 65 we used data from the USA showing that the prevalence of intimate partner violence was 2.2% among people aged 65 or older (27). We therefore estimate that 5.5% of the UK population would be in any of the three states in the Markov model associated with abuse in the first model cycle.

*Transition probabilities*

Table 1 reports all transition probabilities. There are eight transitions between states in the model, measured as follows:

1. No abuse to Abuse not identified

No data were available to reliably estimate this probability. We thus estimated it using the model calibration method described below.

2. Abuse not identified to Abuse identified and seeing advocate

For those receiving the IRIS+ intervention, we estimated this transition probability based on the number of patients seen by the advocate in IRIS+ pilot. Dividing this number by the total number of eligible patients in the seven GP practices (99337 patients) gives a six-month transition probability. For the usual care practices, this probability was estimated based on the



For peer review only - http://bmjopen.bmj.com/site/about/guidelines.xhtml

number of women aged 16+ registered to GP practices in the same area referred to IRIS advocacy (39382 patients).

3. Abuse not identified to Abuse identified, not seeing advocate

We used the ratio of the number of patients abused and identified vs referred in the IRIS+ intervention to estimate the number of patients abused and identified, not seeing advocate. These were effectively patients referred who decline support or who could not be contacted following the referral. The transition probability for usual care (IRIS intervention) was calculated as above, but only considered women identified vs referred.

4. Abuse not identified to No abuse

No data was available to reliably estimate this probability. We therefore estimated this using the model calibration method described below.

5. Abuse identified and seeing advocate to No abuse

This was taken from the MOSAIC (mothers' advocates in the community) trial (25), identified in a Cochrane review (28), evaluating the reduction of any type of domestic abuse with any type of advocacy.

6. Abuse identified, not seeing advocate to No abuse

This was taken from the control arm of the MOSAIC trial (25).

7. No abuse to Dead

We relied on the death rate per 1000 as estimated by the Office for National Statistics. For 2019, it was estimated at 10.4 per 1000. This implies the rate of dying per 6 months is 5.2 per 1000 people, excluding domestic homicides.

8. Abused to Dead

For patients experiencing abuse this probability was 5.54 per 1000 (figure including domestic homicides) per 6 months. This estimate uses the Office for National Statistics death rate for 2019, including domestic homicides. For the purposes of the cost-effectiveness model patients



For peer review only - http://bmjopen.bmj.com/site/about/guidelines.xhtml

could not transition between the health states ‘abuse identified, not seeing advocate’ and ‘abuse identified and seeing advocate’. This is because advocacy and support was offered to identified patients at point of referral and not re-offered. A patient could in principle self-refer into the support service later. But if a patient self-referred after being identified by GP practice teams within 6 months, this would be considered a repeat referral and excluded from the model.

*Model calibration*

We used the prevalence of abuse (5.5%) to calibrate the model, since there was uncertainty surrounding transition probabilities for ‘No abuse to Abuse not identified’ and vice versa. The calibration was run for 3000 cycles, assuming that after this, the number of patients in each state would remain constant. The transition probabilities for ‘No abuse to Abuse not identified’ and vice versa were changed until the proportion of patients in the ‘No abuse’ state exactly reflected the observed prevalence (100-5.5=94.5%). The initial steady state calculation showed that that the probabilities from ‘No abuse to Abuse not identified’ and ‘Abuse not identified to No abuse’ needed adjusting. We assumed some patient would no longer be exposed to abuse naturally and increased the probability of ‘Abuse not identified to No abuse’ from 0.005 to 0.033. To compensate for this increase, we increase the probability of ‘No abuse to Abuse not identified’ from 0.0027 to 0.0033. These adjustments meant that the model better reflected the population prevalence of abuse. The initial distribution of patients in the three ‘Abused’ were 94.5% in ‘No abuse’, 5.3% in ‘Abuse not identified’, 0.018% in ‘Abuse identified and seeing advocate’ and 0.18% in ‘Abuse identified, not seeing advocate’.

*Utilities*

Each state in the Markov model was associated with a utility score (Table 2), allowing us to measure QALYs associated with IRIS+ and usual care (IRIS) based on the proportion of patients in each health state in each of the 20 cycles in the model. Utility scores were separately



For peer review only - http://bmjopen.bmj.com/site/about/guidelines.xhtml

collected and calculated for men, women, and children. For the health state 'No abuse' the utility was assumed to be 0.85 for adults and 0.95 for children, following published population norms (29). A subset of adults and children identified from the IRIS+ intervention filled in a SF-12 and CHU-9D form, respectively. If support/ advocacy was accepted, questionnaire data were requested at: (1) baseline, defined as when support/ advocacy started; and (2) between 6-10 months follow up, defined as the period when support/ advocacy ended. A validated mapping algorithm was used to transform SF-12 scores to SF-6D utilities (30). The published SF-6D utilities were derived from a representative sample (n=611) of the UK adult population using the standard gamble valuation method. Similarly, a published value set was used to transform CHU-9D scores into utilities (31). The published CHU-9D value set was derived from members of the UK adult population (n=300) using both standard gamble and ranking valuation methods. Estimated scores at baseline were attributed to 'Abuse identified, not seeing advocate'. Follow up scores were attributed to 'Abuse identified and seeing advocate'. Due to the small number of forms collected (n=30 at baseline; n=16 at follow up), this data was compared with previous literature for women for sense checking (32). For 'Abuse not identified', we assumed the utility score was the same as 'Abuse identified, not seeing advocate', based on the assumption that identification alone (without advocacy support) does not improve quality of life.

*Costs*

We included: intervention costs; costs of onward referral; and costs associated with abuse (including costs to the UK NHS, costs of lost economic output, costs to the criminal/civil justice system, personal costs) (Table 2). Intervention costs were taken from the budget of the programme. The total budget for the delivery of IRIS+ was £60,253 and included salaries of the advocate educator and children worker, travel and consumables. This was divided by the



For peer review only - http://bmjopen.bmj.com/site/about/guidelines.xhtml

total patient population exposed to the intervention (79485 patients). The cost of onward referral considered the time an advocate educator or a children worker may spend working with external agencies (on average 57 hours), where their support alone would not suffice, multiplied by their average hourly salary (£29.60), and by 39%, which was the proportion of patients referred to the advocate or children's worker who accepted support and needed to be referred to another agency (57 x £29.60 x 0.39 = £658). The cost of onward referral included the cost of referring men to the perpetrator programme. IRIS+ identified five men perpetrators, of which three engaged with the advocate educator. Of these, two accepted an onward referral to a perpetrator programme after risk assessment.

Costs associated with domestic violence in the UK for people aged 16+ is described in Oliver et al (33). In this report, costs of lost economic output, health services, criminal justice system, civil justice system, social welfare, personal costs, specialised services and physical/emotional harm were included, and unit cost per victim per year is estimated at £34,015 (in 2019 prices). We excluded costs of physical/ emotional harm (£24,300), because in its report, Oliver et al calculate cost of physical/ emotional harm by monetising QALY detriments. Since QALY gains are estimated for the intervention, including monetised QALY detriments in cost was deemed inappropriate. This, however, implies that our results are conservative. For adults, the cost of abuse per 6 months was £4,858. For children, we relied on a report produced by Pro Bono Economics (20), which estimated the cost of domestic violence per child to be £1,950 per 6 months in 2018£. We inflated this estimate (£1,969 in 2019£). We considered children to account for 20% of the UK population and estimated an overall cost of abuse per victim of £4,276 (£4858 x 0.8 – adults + £1969 x 0.2 – children) per 6 months.



For peer review only - http://bmjopen.bmj.com/site/about/guidelines.xhtml

**Table 2. Model input parameters: utilities and costs**

| Parameter | Base case value | Lower limit | Upper limit | Distribution | Source |
|---|---|---|---|---|---|
| ***Utilities*** | | | | | |
| Not abused (adults) | 0.850 | 0.840 | 0.860 | Beta | (29) |
| Not abused (children) | 0.950 | 0.940 | 0.959 | Beta | (29) |
| Abused but not identified (women) | 0.656 | 0.522 | 0.749 | Beta | Assumption |
| Abused but not identified (men) | 0.626 | 0.500 | 0.744 | Beta | Assumption |
| Abused but not identified (children) | 0.801 | 0.623 | 0.932 | Beta | Assumption |
| Abused and identified, seeing advocate (women) | 0.659 | 0.518 | 0.782 | Beta | IRIS+ data |
| Abused and identified, seeing advocate (men) | 0.701 | 0.555 | 0.828 | Beta | IRIS+ data |
| Abused and identified, seeing advocate (children) | 0.804 | 0.625 | 0.935 | Beta | IRIS+ data |
| Abused and identified, not seeing advocate (women) | 0.656 | 0.522 | 0.749 | Beta | IRIS+ data |
| Abused and identified, not seeing advocate (men) | 0.626 | 0.500 | 0.744 | Beta | IRIS+ data |
| Abused and identified, not seeing advocate (children) | 0.801 | 0.623 | 0.932 | Beta | IRIS+ data |
| ***Costs (2019/20£)*** | | | | | |
| Costs of the intervention, per patient exposed to DV, per 6 months | £0.75 | £0.02 | £2.73 | Gamma | IRIS+ budget |



For peer review only - http://bmjopen.bmj.com/site/about/guidelines.xhtml

| | | | | | |
|---|---|---|---|---|---|
| Cost of onward referral, once | £658 | £11 | £1908 | Gamma | IRIS+ data and IRIS data |
| Cost of Abused but not identified (weighted average – exposed population) | £4276 | £108 | £15774 | Gamma | **weighted average |
| Cost of Abused but not identified (adults) | £4858 | £123 | £17919 | Gamma | (33) |
| Cost of Abused but not identified (children) | £1950 | £1000 | £2500 | Gamma | (20) |
| Weighted costs Abused and identified, seeing advocate | 1 | 0.75 | 1.25 | Gamma | Assumption |
| Weighted costs Abused and identified, not seeing advocate | 1 | 0.9 | 1.1 | Gamma | Assumption |

Costs are in 2019/20 UK£. **Excludes the cost of harms, which in this modelled are measured as benefits

For peer review only

*Cost-utility analysis*

A cost-utility analysis was conducted comparing costs and QALYs for IRIS+ versus IRIS (usual care). QALYs were calculated from utilities by using the area under the curve approach. The main outcome was the Net Monetary Benefit (NMB), that estimates both costs and QALYs in monetary terms, using an acceptability threshold of £20,000 per QALY. A positive incremental NMB result indicates that IRIS+ intervention would be preferred on cost-effectiveness grounds. While a negative incremental NMB result indicates that the IRIS intervention(usual care) would be preferred. Results were also shown in terms of the incremental costs per QALY gained of IRIS+ vs IRIS. This was measured as the mean difference in costs between IRIS+ and IRIS divided by the mean difference in QALYs. We followed the usual decision making rule for cost-effectiveness in the UK, in which an intervention is likely to be considered cost-effective when the incremental costs per QALY gained are less than £20,000 (19).

*Subgroups and distributional effects*

The IRIS+ and IRIS arms represented two key groups which could be targeted in primary care (women, men and their children vs women only). Consequently, we did not estimate cost-effectiveness for any alternative subgroups. DVA is experienced across all social groups including all different socioeconomic, ethnicity and geographical groups. The IRIS and IRIS+ interventions are designed for all social groups, therefore we did not consider distributional effects.

*Sensitivity analysis*



For peer review only - http://bmjopen.bmj.com/site/about/guidelines.xhtml

We undertook a probabilistic sensitivity analysis, based on 1000 simulations drawn from random samples from the probability distributions of all parameters. These 1000 simulations were plotted in a cost-effectiveness plane. The proportion of simulations with an incremental cost per QALY gained below the cost-effectiveness threshold was calculated for different threshold values, ranging from £0 to £50,000. The results were presented in a cost-effectiveness acceptability curve.

*Patient and public involvement*

Three patient and public involvement (PPI) groups (female survivors, male survivors and male perpetrators) were created and consulted throughout the lifetime of the research programme. PPI representatives were involved in the development of the IRIS+ intervention and the design of the research study.

## Results

*Base case*

The results of the cost-utility in the base case analysis are presented in Table 3. Over the 10-year time horizon, mean total costs per patient registered at general practices eligible to the IRIS+ intervention were £3,867. For the IRIS intervention (usual care), the mean cost per patient was £3,959. IRIS+ therefore could potentially save £92 per patient over a 10-year time horizon. Total QALYs per patient were also 0.003 higher in the IRIS+ arm (7.000) than in the IRIS arm (usual care) (6.997). As the IRIS+ intervention arm was associated with lower costs and higher effectiveness then the incremental cost per QALY gained was negative (dominating usual care, IRIS) and the incremental NMB was positive (£145).



For peer review only - http://bmjopen.bmj.com/site/about/guidelines.xhtml

**Table 3. Discounted base case and probabilistic results**

| | | | |
|---|---|---|---|
| Discounted base case results | | | |
| | **Costs** | **QALYs** | **Cost-effectiveness** |
| Intervention (IRIS+ programme) | £3,867 | 7.000 | |
| Control (IRIS programme) | £3,959 | 6.997 | |
| Difference (intervention vs. control) | -£92 | 0.003 | -ve (intervention dominates control) |
| Incremental NMB* | | | £145 |
| Probabilistic results | | | |
| | **Costs (95% CI)** | **QALYs (95% CI)** | **Cost-effectiveness (95% CI)** |
| Intervention (IRIS+ programme) | £107 to £16616 | 6.377 to 7.192 | |
| Control (IRIS programme) | £104 to £17343 | 6.377 to 7.197 | |
| Increment | £-1123 to £171 | -0.030 to 0.019 | |
| ICER | | | £-206828 to £277989 |

QALY = quality-adjusted life year. NMB = net monetary benefit. ICER= incremental cost effectiveness ratio. Costs are in 2019/20 UK£. Numbers may not sum due to rounding. *Measured at a willingness to pay for a QALY of £20 000.



For peer review only - http://bmjopen.bmj.com/site/about/guidelines.xhtml

*Sensitivity analysis*

Incremental costs and QALYs varied widely in the probabilistic sensitivity analyses. The 95% confidence interval for incremental costs was -£1,123 to £171, while for incremental QALYs it was -0.030 to 0.019, and the ICER was -£206,828 to £277,989 per QALY gained. Figure 2(a) shows a scatter plot of the incremental costs and incremental QALYs from the 1000 simulations. It shows how much uncertainty there is around these results. The IRIS+ intervention was cost-effective in 55% of simulations when the cost-effectiveness threshold was £20, 000 (Figure 2(b)).

## Discussion

We found that the IRIS+ intervention is likely to be cost-effective or even cost-saving from a societal perspective in the UK with a willingness-to-pay threshold of £20,000 per gain in QALY, when compared to usual care (IRIS). There is considerable uncertainty surrounding these results, but there was more than a 50% probability that IRIS+ is likely to be cost-effective at £20,000 per QALY, the cost-effectiveness threshold commonly used in the UK.

There are a number of strengths and limitations to this study. The main strength relates to this study drawing on newly collected data, reducing the need for using out-of-date previously published estimates. It, however, relies on a small number of observations (n=30 at baseline; n=16 at follow up), which could potentially be unreliable. The large uncertainty in our results reflects the small sample size. Nevertheless, as far as the authors are aware, this is the first study to assess the potential cost-effectiveness of a primary care intervention providing support to not just women, but also men and children experiencing DVA. Another important limitation of this study relates to its prospective nature. Given the pilot design, we were unable to directly recruit practices into IRIS+ and IRIS (usual care). Thus, by using practices in the same area,



For peer review only

spillover effects may be significant (although they were not explored in this paper). Furthermore, the small number of practices, and as a result the small number of patients identified, meant that subgroup analysis was not possible. A cluster randomised control trial (RCT) comparing IRIS+ to IRIS (usual care) could potentially address some of the uncertainties observed in the cost-effectiveness result of this study. More specifically, an economic evidence collected alongside a trial may shed light onto some of the differences in terms of costs and benefits for women, men and children.

Comparing this prospective study to similar studies in the literature is challenging. Most training and advocacy programmes evaluated to date, have focused on a subset of the population, such as women, children or men only. Including all groups is a key strength of the IRIS+ intervention, as reported in the qualitative findings of this research study (34). Future research should attempt to replicate the intervention in a greater number of general practices across the UK to enable more robust data collection and larger sample sizes.

**Contribution statement**

Cochrane attests that all listed authors meet authorship criteria and that no others meeting the criteria have been omitted from the opportunity to be listed as an author.MC, ES, CC, EE, MJ, GF and EB contributed to the planning of the study. ES and CC managed the coordination of the study. MC and EB conducted the analysis for the study and MC, EB, ES and EE, GF contributed to the interpretation of the data. MC and EB developed the manuscript. MC, ES, CC, EE, MJ, GF and EB read and commented on the manuscript drafts and approved the final manuscript. GF was the Chief Investigator of the study. The study was funded by National Institute for Health Research (Programme Grants for Applied Research), (RP-PG-0614-20012).



For peer review only - http://bmjopen.bmj.com/site/about/guidelines.xhtml

**Competing interests**

Medina Johnson is the CEO of IRISi and was a named partner in REPROVIDE. She did not influence the economic modelling or its results.

**Funding**

IRIS+ is part of the REPROVIDE programme (Reaching Everyone Programme of Research On Violence in diverse Domestic Environments), an independent research programme funded by the National Institute for Health Research (Programme Grants for Applied Research), (RP-PG-0614-20012). The views expressed in this publication are those of the authors and not necessarily those of the National Institute for Health Research or the Department of Health and Social Care. The funder had no role in the design and conduct of the study; collection, management, analysis, and interpretation of the data; preparation, review, or approval of the manuscript; and decision to submit the manuscript for publication.

**Data sharing and data availability statement**

All data requests should be submitted to the corresponding author for consideration. Access to anonymised data may be granted following review.

**Ethics approval statement**

The study was given favourable ethical approval by London - Hampstead Research Ethics Committee (REC reference: 19/LO/1132) and the Health Research Authority (HRA) and Health and Care Research Wales (HCRW).



For peer review only

For peer review only - http://bmjopen.bmj.com/site/about/guidelines.xhtml

## References


1. Alliance VP. The public health approach. World Health Organisation (https://www who int/violenceprevention/approach/public_health/en/) Accessed. 2020;9.

2. Chandan JS, Taylor J, Bradbury-Jones C, Nirantharakumar K, Kane E, Bandyopadhyay S. COVID-19: a public health approach to manage domestic violence is needed. The Lancet Public Health. 2020;5(6):e309.

3. WHO. Violence against women: A global health problem of epidemic proportions Geneva: World Health Organization; 2013 [Available from: http://www.who.int/mediacentre/news/releases/2013/violence_against_women_20130620/en/.

4. Rakovec-Felser Z. Domestic violence and abuse in intimate relationship from public health perspective. Health psychology research. 2014;2(3).

5. Oliver R, Alexander, B., Roe, S., Wlasny, M. The economic and social costs of domestic abuse. London; 2019.

6. SafeLives. A Safe Fund: costing domestic abuse provision for the whole family: SafeLives; 2020 [Available from: https://safelives.org.uk/sites/default/files/A%20Safe%20Fund%20costing%20domestic%20abuse%20provision%20for%20the%20whole%20family%20in%20England%20and%20Wales_0.pdf.

7. Cleaver K, Maras P, Oram C, McCallum K. A review of UK based multi-agency approaches to early intervention in domestic abuse: Lessons to be learnt from existing evaluation studies. Aggression and violent behavior. 2019;46:140-55.




For peer review only - http://bmjopen.bmj.com/site/about/guidelines.xhtml

8. Hamberger LK, Larsen SE. Men’s and women’s experience of intimate partner violence: A review of ten years of comparative studies in clinical samples; Part I. Journal of Family Violence. 2015;30(6):699-717.

9. Walby S, Towers J, Francis B. Is violent crime increasing or decreasing? A new methodology to measure repeat attacks making visible the significance of gender and domestic relations. British Journal of Criminology. 2016;56(6):1203-34.

10. Feder G, Davies RA, Baird K, Dunne D, Eldridge S, Griffiths C, et al. Identification and Referral to Improve Safety (IRIS) of women experiencing domestic violence with a primary care training and support programme: a cluster randomised controlled trial. The Lancet. 2011;378(9805):1788-95.

11. Holt S, Buckley H, Whelan S. The impact of exposure to domestic violence on children and young people: A review of the literature. Child abuse & neglect. 2008;32(8):797-810.

12. McTavish JR, MacGregor JC, Wathen CN, MacMillan HL. Children’s exposure to intimate partner violence: An overview. International review of psychiatry. 2016;28(5):504-18.

13. Coker AL, Davis KE, Arias I, Desai S, Sanderson M, Brandt HM, et al. Physical and mental health effects of intimate partner violence for men and women. American journal of preventive medicine. 2002;23(4):260-8.

14. ONS. Domestic abuse victim characteristics, England and Wales: year ending March 2020. Characteristics of victims of domestic abuse based on findings from the Crime Survey for England and Wales and police recorded crime. 2020 12th May 2022.



For peer review only - http://bmjopen.bmj.com/site/about/guidelines.xhtml

15. Hester M, Ferrari G, Jones SK, Williamson E, Bacchus LJ, Peters TJ, et al. Occurrence and impact of negative behaviour, including domestic violence and abuse, in men attending UK primary care health clinics: a cross-sectional survey. 2015;5(5).

16. Hester M, Williamson, E., Regan, L., Coulter, M., Chantler, K., Gangoli, G., Davenport, R., Green, L. . Exploring the service and support needs of male, lesbian, gay, bi-sexual and transgendered and black and other minority ethnic victims of domestic
and sexual violence. Report prepared for Home Office SRG/06/017. Bristol; 2012.

17. Szilassy E, Caroline, C., Roy, J. Elizabeth, E., Gene, F. Feasibility of extending the IRIS domestic violence model 4th European Conference on Domestic Violence; Ljubljana: ECDV; 2021.

18. Roy J, Williamson E, Pitt K, Stanley N, Man MS, Feder G, et al. 'It felt like there was always someone there for us': Supporting children affected by domestic violence and abuse who are identified by general practice. Health & Social Care in the Community. 2022;30(1):165-74.

19. National Institute for Health and Care Excellence. Developing NICE guidelines: the manual. 2022 15th December 2022.

20. Pro Bono Economics. The economic cost from childhood exposure to severe domestic violence. 2018 15th December 2022.

21. Devine A, Spencer A, Eldridge S, Norman R, Feder G. Cost-effectiveness of Identification and Referral to Improve Safety (IRIS), a domestic violence training and support programme for primary care: a modelling study based on a randomised controlled trial. BMJ open. 2012;2(3).



For peer review only - http://bmjopen.bmj.com/site/about/guidelines.xhtml

22. Barbosa EC, Verhoef TI, Morris S, Solmi F, Johnson M, Sohal A, et al. Cost-effectiveness of a domestic violence and abuse training and support programme in primary care in the real world: updated modelling based on an MRC phase IV observational pragmatic implementation study. BMJ open. 2018;8(8):e021256.

23. Feder G, Davies RA, Baird K, Dunne D, Eldridge S, Griffiths C, et al. Identification and Referral to Improve Safety (IRIS) of women experiencing domestic violence with a primary care training and support programme: a cluster randomised controlled trial. Lancet (London, England). 2011;378(9805):1788-95.

24. Gilbert R, Widom CS, Browne K, Fergusson D, Webb E, Janson S. Burden and consequences of child maltreatment in high-income countries. The lancet. 2009;373(9657):68-81.

25. Taft AJ, Small R, Hegarty KL, Watson LF, Gold L, Lumley JA. Mothers' AdvocateS In the Community (MOSAIC)--non-professional mentor support to reduce intimate partner violence and depression in mothers: a cluster randomised trial in primary care. BMC public health. 2011;11:178.

26. ONS. Analysis of population estimates tool 2020 [Available from: https://www.ons.gov.uk/peoplepopulationandcommunity/populationandmigration/populationestimates/datasets/analysisofpopulationestimatestool.

27. Bonomi AE, Anderson ML, Reid RJ, Carrell D, Fishman PA, Rivara FP, et al. Intimate partner violence in older women. Gerontologist. 2007;47(1):34-41.

28. Rivas C, Ramsay J, Sadowski L, Davidson LL, Dunne D, Eldridge S, et al. Advocacy interventions to reduce or eliminate violence and promote the physical and psychosocial



For peer review only - http://bmjopen.bmj.com/site/about/guidelines.xhtml

well-being of women who experience intimate partner abuse. status and date: New search for studies and content updated (no change to conclusions), published in. 2015(12).

29. Kind K, Hardman G, Macran S. UK Population norms for EQ-5D. University of York; 1999.

30. Brazier JE, Roberts J. The estimation of a preference-based measure of health from the SF-12. Medical care. 2004;42(9):851-9.

31. Stevens K. Valuation of the Child Health Utility 9D Index. Pharmacoeconomics. 2012;30(8):729-47.

32. Wittenberg E, Lichter EL, Ganz ML, McCloskey LA. Community preferences for health states associated with intimate partner violence. Medical care. 2006;44(8):738-44.

33. Oliver R, Alexander B, Roe S, Wlasny M. The economic and social costs of domestic abuse. Home Office (UK). 2019.

34. Szilassy E, Roy J, Williamson E, Pitt K, Man M-S, Feder G. Reaching everyone in general practice? Feasibility of an integrated domestic violence training and support intervention in primary care. BMC family practice. 2021;22(1):1-17.

For peer review only

**Figures**

Figure 1. Health states and movement between health states in Markov model.

The model starts with all patients in either the ‘No abuse’ state or one of the states associated with abuse, based on the prevalence of DV (see text). Patients in the ‘No abuse’ state could stay in this state, move to ‘Abuse not identified’ or die from any cause. Once a patient is in the ‘Abuse not unidentified’ state, they could stay in that state, move back to ‘No abuse’, move to



For peer review only - http://bmjopen.bmj.com/site/about/guidelines.xhtml

‘Abuse identified and seeing advocate’ or ‘Abuse identified, not seeing advocate’ or die. Patients in the ‘Abuse identified’ states could stay in these states, move back to ‘No abuse’ or die. Death is an absorbing state.

Figure 2. Probabilistic sensitivity analysis.

(a) Scatter plot of incremental costs and incremental QALYs from 1000 simulations.

(b) Cost-effectiveness acceptability curve showing the probability that the intervention (IRIS+) is cost-effective vs. control (IRIS) at different values of the maximum willingness to pay for a QALY. QALY = quality-adjusted life year. Costs are in 2019/20 UK£.

For peer review only



For peer review only - http://bmjopen.bmj.com/site/about/guidelines.xhtml

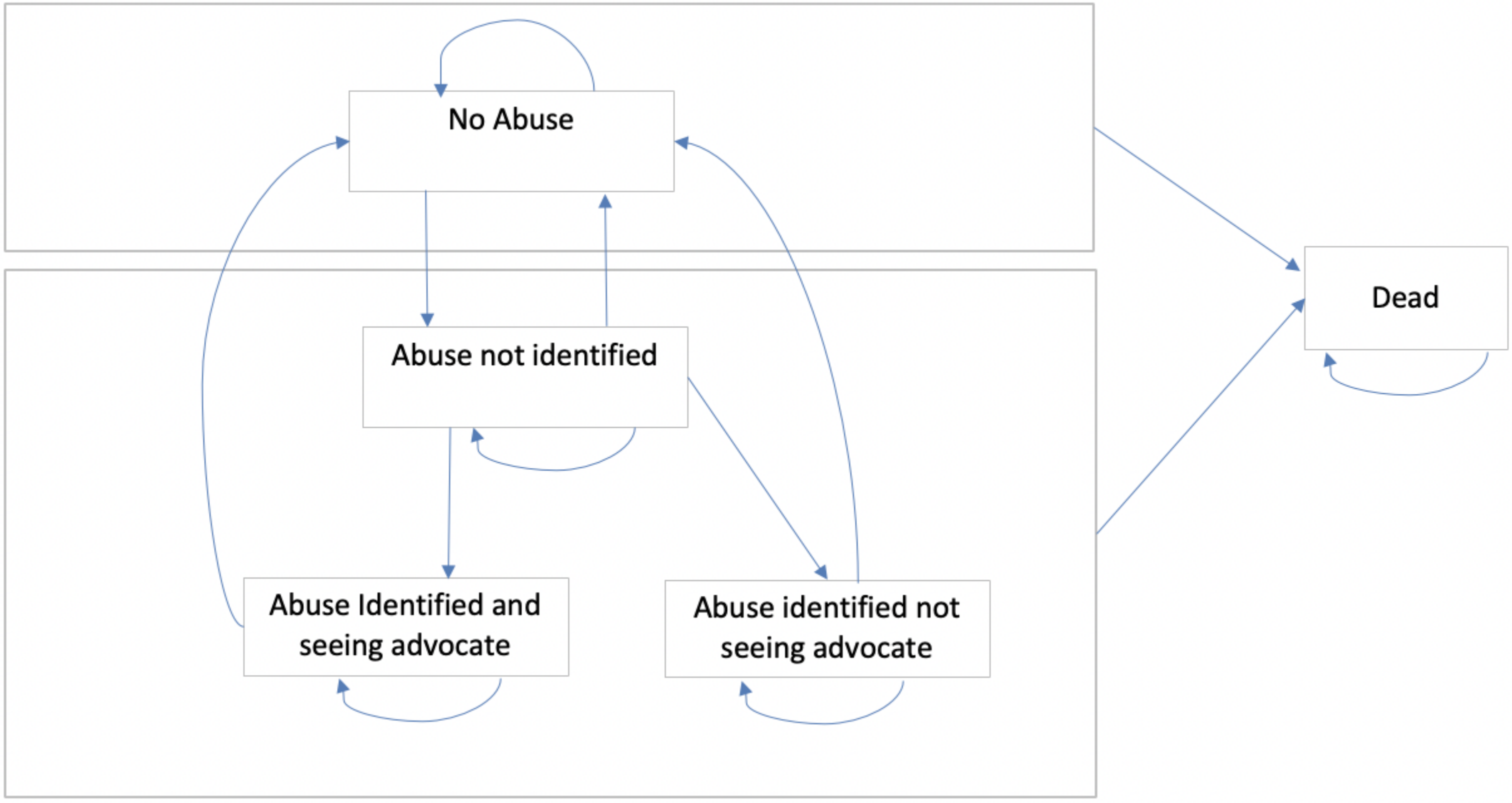
No Abuse
Abuse not identified
Abuse Identified and seeing advocate
Abuse identified not seeing advocate
Dead

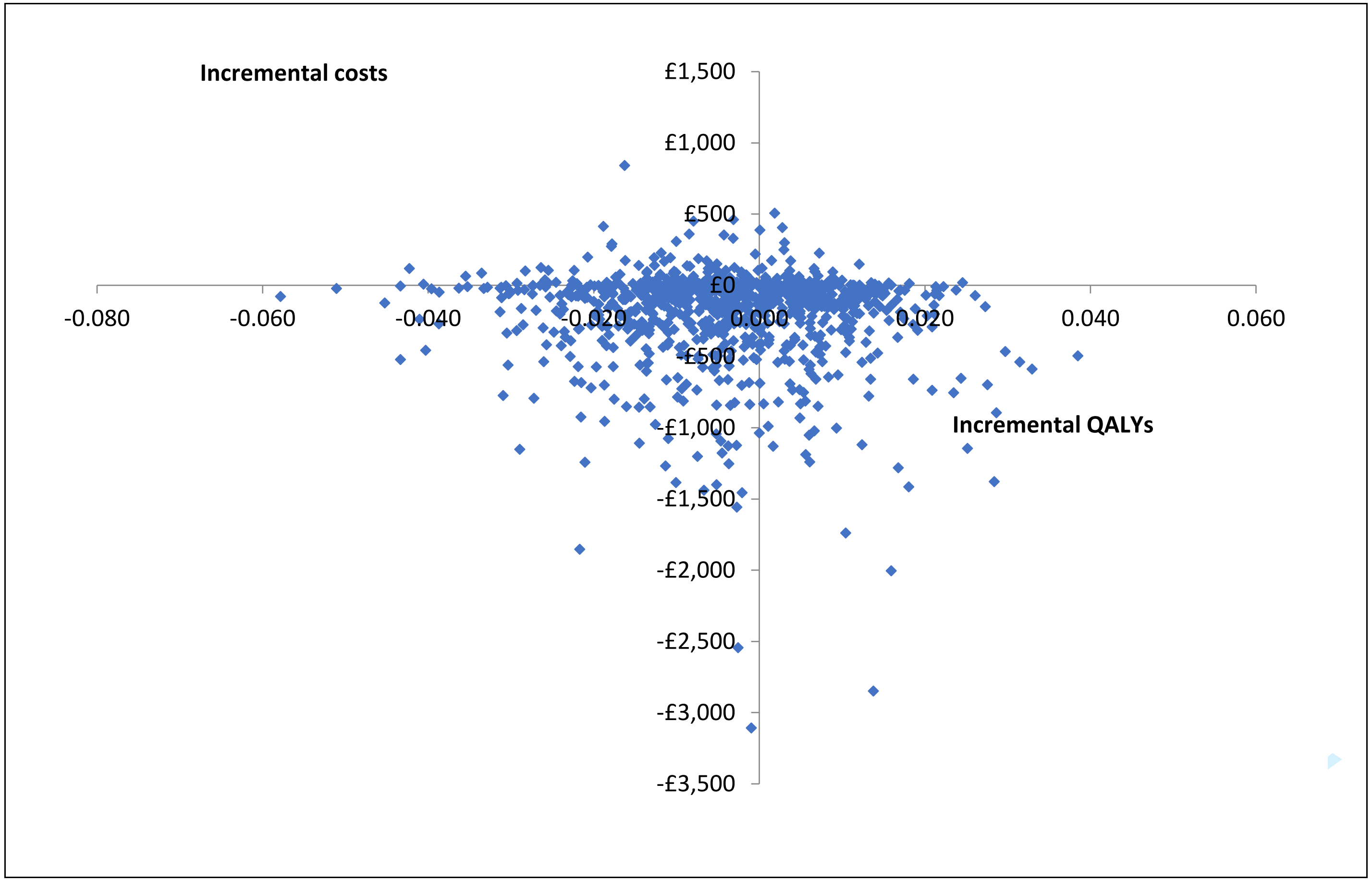
Incremental costs
£1,500
£1,000
£500
£0
-£500
-£1,000
-£1,500
-£2,000
-£2,500
-£3,000
-£3,500
-0.080
-0.060
-0.040
-0.020
0.000
0.020
0.040
0.060
Incremental QALYs

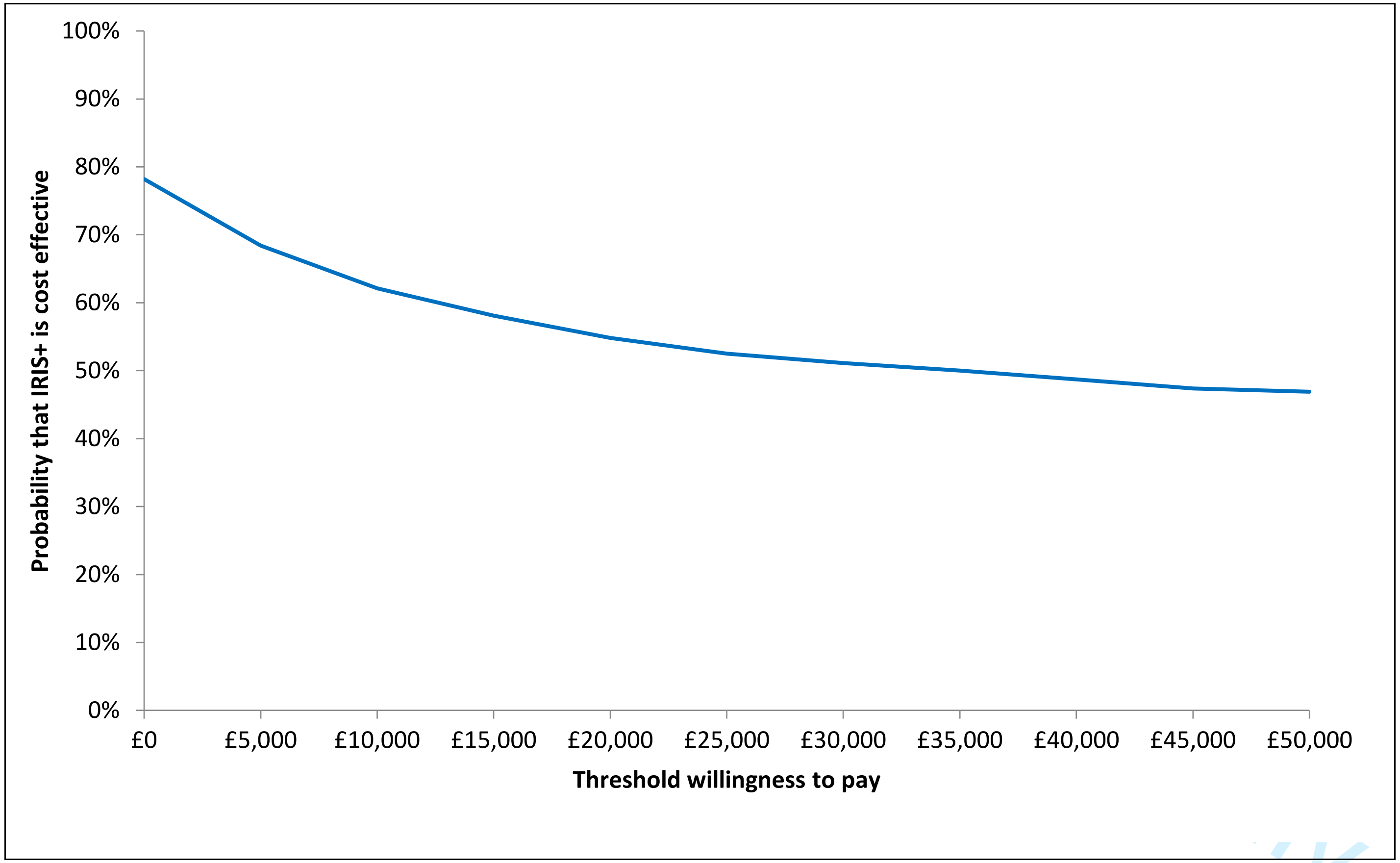
Probability that IRIS+ is cost effective
100%
90%
80%
70%
60%
50%
40%
30%
20%
10%
0%
£0
£5,000
£10,000
£15,000
£20,000
£25,000
£30,000
£35,000
£40,000
£45,000
£50,000
Threshold willingness to pay

**Primary care system-level training and support programme for the secondary prevention of domestic violence and abuse: a cost-effectiveness feasibility model**

Madeleine Cochrane, Eszter Szilassy, Caroline Coope, Elizabeth Emsley, Medina Johnson, Gene Feder and Estela Capelas Barbosa

For peer review only

**Table S1. Adult's model input parameters: probabilities; utilities; and, costs.**

| Parameter | Base case value | Lower limit | Upper limit | Distribution | Source |
|---|---|---|---|---|---|
| ***Probabilities*** | | | | | |
| Prevalence of DVA in adults (males and females) – aged 16 to 65 | 0.055 | 0.036 | 0.073 | Beta | ONS, 2020b |
| *Starting distribution for patients who are abused* | | | | | |
| Abused and identified, seeing advocate | 0.003 | 0 | 0.0066 | Uniform | * |
| Abused and identified, not seeing advocate | 0.033 | 0 | 0.0660 | Uniform | * |
| Abused but not identified | 0.964 | - | - | Uniform | Complement |
| *Transition probabilities* | | | | | |
| Not abused to Abused but not identified | 0.0037 | 0.0004 | 0.0106 | Dirichlet | * |
| Not abused to Dead | 0.0052 | 0.0027 | 0.0087 | Dirichlet | ONS, 2020b |
| Stay in Not abused | 0.9911 | - | - | Dirichlet | Complement |
| Abused but not identified to Not abused (control) | 0.0500 | 0.0412 | 0.0596 | Dirichlet | * |
| Abused but not identified to Abused and identified, not seeing advocate (control) | 0.0091 | 0.0055 | 0.0135 | Dirichlet | IRIS data |

For peer review only

| | | | | | |
|---|---|---|---|---|---|
| Abused but not identified to Abused and identified, seeing advocate (control) | 0.0226 | 0.0168 | 0.0293 | Dirichlet | IRIS data |
| Abused but not identified to Dead (control) | 0.0055 | 0.0029 | 0.0091 | Dirichlet | ONS, 2020b |
| Stay in Abused but not identified (control) | 0.9131 | - | - | Dirichlet | Complement |
| Abused but not identified to Not abused (intervention) | 0.0500 | 0.0412 | 0.0596 | Dirichlet | * |
| Abused but not identified to Abused and identified, not seeing advocate (intervention) | 0.0374 | 0.0298 | 0.0458 | Dirichlet | IRIS+ data |
| Abused but not identified to Abused and identified, seeing advocate (intervention) | 0.0312 | 0.0243 | 0.0390 | Dirichlet | IRIS+ data |
| Abused but not identified to Dead (intervention) | 0.0055 | 0.0029 | 0.0091 | Dirichlet | ONS, 2020B |
| Stay in Abused but not identified (intervention) | 0.8762 | - | - | Dirichlet | Complement |
| Abused and identified, seeing advocate to Not abused | 0.1408 | 0.0707 | 0.2301 | Dirichlet | (Taft et al., 2011) |
| Abused and identified, seeing advocate to Dead | 0.0052 | 0.0000 | 0.0299 | Dirichlet | ONS, 2020b |
| Stay in Abused and identified, seeing advocate | 0.854 | - | - | Dirichlet | Complement |
| Abused and identified, not seeing advocate to Not abused | 0.0781 | 0.0136 | 0.1912 | Dirichlet | (Taft et al., 2011) |
| Abused and identified, not seeing advocate to Dead | 0.0052 | 0.0000 | 0.0424 | Dirichlet | ONS, 2020b |

For peer review only

| | | | | | |
|---|---|---|---|---|---|
| Stay in Abused and identified, not seeing advocate | 0.9167 | - | - | Dirichlet | Complement |
| ***Utilities*** | | | | | |
| Not abused (adults) | 0.850 | 0.840 | 0.860 | Beta | (Kind et al., 1999) |
| Abused but not identified (women) | 0.656 | 0.522 | 0.749 | Beta | Assumption |
| Abused but not identified (men) | 0.626 | 0.500 | 0.744 | Beta | Assumption |
| Abused and identified, seeing advocate (women) | 0.659 | 0.518 | 0.782 | Beta | IRIS+ data |
| Abused and identified, seeing advocate (men) | 0.701 | 0.555 | 0.828 | Beta | IRIS+ data |
| Abused and identified, not seeing advocate (women) | 0.656 | 0.522 | 0.749 | Beta | IRIS+ data |
| Abused and identified, not seeing advocate (men) | 0.626 | 0.500 | 0.744 | Beta | IRIS+ data |
| ***Costs (2019/20£)*** | | | | | |
| Costs of the intervention, per patient exposed to DV, per 6 months | £0.75 | £0.02 | £2.73 | Gamma | IRIS+ budget |
| Cost of onward referral, once | £658 | £11 | £1908 | Gamma | IRIS+ data; IRIS data |
| Cost of Abused but not identified (adults) | £4858 | £123 | £17919 | Gamma | (Oliver et al., 2019) |
| Weighted costs Abused and identified, seeing advocate | 1 | 0.75 | 1.25 | Gamma | Assumption |
| Weighted costs Abused and identified, not seeing advocate | 1 | 0.9 | 1.1 | Gamma | Assumption |

Costs are in 2019/20 UK£.
* Internal calculation based on model calibration.

For peer review only

± Excludes the cost of harms, which in this modelled are measured as benefit

**Table S2. Children's model input parameters: probabilities; utilities; and, costs.**

| Parameter | Base case value | Lower limit | Upper limit | Distribution | Source |
|---|---|---|---|---|---|
| ***Probabilities*** | | | | | |
| Prevalence of children exposed to DVA | 0.080 | 0.040 | 0.140 | Beta | (Gilbert et al., 2009) |
| *Starting distribution for patients who are abused* | | | | | |
| Abused and identified, seeing advocate | 0.003 | 0 | 0.0066 | Uniform | * |
| Abused and identified, not seeing advocate | 0.033 | 0 | 0.0660 | Uniform | * |
| Abused but not identified | 0.964 | - | - | Uniform | Complement |
| *Transition probabilities* | | | | | |
| Not abused to Abused but not identified | 0.0037 | 0.0004 | 0.0106 | Dirichlet | * |
| Not abused to Dead | 0.0052 | 0.0027 | 0.0087 | Dirichlet | ONS, 2020b |
| Stay in Not abused | 0.9911 | - | - | Dirichlet | Complement |
| Abused but not identified to Not abused (control) | 0.0500 | 0.0412 | 0.0596 | Dirichlet | * |

For peer review only

| | | | | | |
|---|---|---|---|---|---|
| Abused but not identified to Abused and identified, not seeing advocate (control) | 0.0091 | 0.0055 | 0.0135 | Dirichlet | IRIS data |
| Abused but not identified to Abused and identified, seeing advocate (control) | 0.0226 | 0.0168 | 0.0293 | Dirichlet | IRIS data |
| Abused but not identified to Dead (control) | 0.0055 | 0.0029 | 0.0091 | Dirichlet | ONS, 2020b |
| Stay in Abused but not identified (control) | 0.9131 | - | - | Dirichlet | Complement |
| Abused but not identified to Not abused (intervention) | 0.0500 | 0.0412 | 0.0596 | Dirichlet | * |
| Abused but not identified to Abused and identified, not seeing advocate (intervention) | 0.0374 | 0.0298 | 0.0458 | Dirichlet | IRIS+ data |
| Abused but not identified to Abused and identified, seeing advocate (intervention) | 0.0312 | 0.0243 | 0.0390 | Dirichlet | IRIS+ data |
| Abused but not identified to Dead (intervention) | 0.0055 | 0.0029 | 0.0091 | Dirichlet | ONS, 2020b |
| Stay in Abused but not identified (intervention) | 0.8762 | - | - | Dirichlet | Complement |
| Abused and identified, seeing advocate to Not abused | 0.1408 | 0.0707 | 0.2301 | Dirichlet | (Taft et al., 2011) |
| Abused and identified, seeing advocate to Dead | 0.0052 | 0.0000 | 0.0299 | Dirichlet | ONS, 2020b |
| Stay in Abused and identified, seeing advocate | 0.854 | - | - | Dirichlet | Complement |

For peer review only

| | | | | | |
|---|---|---|---|---|---|
| Abused and identified, not seeing advocate to Not abused | 0.0781 | 0.0136 | 0.1912 | Dirichlet | (Taft et al., 2011) |
| Abused and identified, not seeing advocate to Dead | 0.0052 | 0.0000 | 0.0424 | Dirichlet | ONS, 2020b |
| Stay in Abused and identified, not seeing advocate | 0.9167 | - | - | Dirichlet | Complement |
| ***Utilities*** | | | | | |
| Not abused (children) | 0.950 | 0.940 | 0.959 | Beta | (Kind et al., 1999) |
| Abused but not identified (children) | 0.801 | 0.623 | 0.932 | Beta | Assumption |
| Abused and identified, seeing advocate (children) | 0.804 | 0.625 | 0.935 | Beta | IRIS+ data |
| Abused and identified, not seeing advocate (children) | 0.801 | 0.623 | 0.932 | Beta | IRIS+ data |
| ***Costs (2019/20£)*** | | | | | |
| Costs of the intervention, per patient exposed to DV, per 6 months | £0.75 | £0.02 | £2.73 | Gamma | IRIS+ budget |
| Cost of onward referral, once | £658 | £11 | £1908 | Gamma | IRIS+ data; IRIS data |
| Cost of Abused but not identified (children) | £1950 | £1000 | £2500 | Gamma | (Pro Bono Economics, 2018) |
| Weighted costs Abused and identified, seeing advocate | 1 | 0.75 | 1.25 | Gamma | Assumption |
| Weighted costs Abused and identified, not seeing advocate | 1 | 0.9 | 1.1 | Gamma | Assumption |

Costs are in 2019/20 UK£. * Internal calculation based on model calibration. ± Excludes the cost of harms, which in this modelled are measured as benefit

For peer review only

For peer review only

# CHEERS 2022 Checklist

| Topic | No. | Item | Location where item is reported |
|---|---|---|---|
| **Title** | | | |
| **Title** | 1 | Identify the study as an economic evaluation and specify the interventions being compared. | Title, Page 1 |
| **Abstract** | | | |
| **Abstract** | 2 | Provide a structured summary that highlights context, key methods, results, and alternative analyses. | Abstract, Page 1 |
| **Introduction** | | | |
| **Background and objectives** | 3 | Give the context for the study, the study question, and its practical relevance for decision making in policy or practice. | Background, Page 4 |
| **Methods** | | | |
| **Health economic analysis plan** | 4 | Indicate whether a health economic analysis plan was developed and where available. | Methods, Page 5 |
| **Study population** | 5 | Describe characteristics of the study population (such as age range, demographics, socioeconomic, or clinical characteristics). | Methods, Page 7 |
| **Setting and location** | 6 | Provide relevant contextual information that may influence findings. | Methods, Page 6 |
| **Comparators** | 7 | Describe the interventions or strategies being compared and why chosen. | Methods, Page 6-7 |
| **Perspective** | 8 | State the perspective(s) adopted by the study and why chosen. | Methods, Page 5 |
| **Time horizon** | 9 | State the time horizon for the study and why appropriate. | Methods, Page 5 |
| **Discount rate** | 10 | Report the discount rate(s) and reason chosen. | Methods, Page 6 |
| **Selection of outcomes** | 11 | Describe what outcomes were used as the measure(s) of benefit(s) and harm(s). | Methods, Page 5 |
| **Measurement of outcomes** | 12 | Describe how outcomes used to capture benefit(s) and harm(s) were measured. | Methods, Page 10 |
| **Valuation of outcomes** | 13 | Describe the population and methods used to measure and value outcomes. | Methods, Page 10 |



For peer review only - http://bmjopen.bmj.com/site/about/guidelines.xhtml

| Topic | No. | Item | Location where item is reported |
|---|---|---|---|
| **Measurement and valuation of resources and costs** | 14 | Describe how costs were valued. | Methods, Page 11 |
| **Currency, price date, and conversion** | 15 | Report the dates of the estimated resource quantities and unit costs, plus the currency and year of conversion. | Methods, Page 5 |
| **Rationale and description of model** | 16 | If modelling is used, describe in detail and why used. Report if the model is publicly available and where it can be accessed. | Methods, Page 6 |
| **Analytics and assumptions** | 17 | Describe any methods for analysing or statistically transforming data, any extrapolation methods, and approaches for validating any model used. | Methods, Table 1 and Page 6-11 |
| **Characterising heterogeneity** | 18 | Describe any methods used for estimating how the results of the study vary for subgroups. | Methods, Page 12 |
| **Characterising distributional effects** | 19 | Describe how impacts are distributed across different individuals or adjustments made to reflect priority populations. | Methods, Page 12 |
| **Characterising uncertainty** | 20 | Describe methods to characterise any sources of uncertainty in the analysis. | Methods, Page 12 |
| **Approach to engagement with patients and others affected by the study** | 21 | Describe any approaches to engage patients or service recipients, the general public, communities, or stakeholders (such as clinicians or payers) in the design of the study. | Methods, Page 6 |
| Results | | | |
| **Study parameters** | 22 | Report all analytic inputs (such as values, ranges, references) including uncertainty or distributional assumptions. | Results, Table 1 |
| **Summary of main results** | 23 | Report the mean values for the main categories of costs and outcomes of interest and summarise them in the most appropriate overall measure. | Results, Table 2 |
| **Effect of uncertainty** | 24 | Describe how uncertainty about analytic judgments, inputs, or projections affect findings. Report the effect of choice of discount rate and time horizon, if applicable. | Results, Figure 2 |
| **Effect of engagement with patients and others affected by the study** | 25 | Report on any difference patient/service recipient, general public, community, or stakeholder involvement made to the approach or findings of the study | Not applicable as model was based on previous study. |
| **Discussion** | | | |
| **Study findings, limitations, generalisability, and current knowledge** | 26 | Report key findings, limitations, ethical or equity considerations not captured, and how these could affect patients, policy, or practice. | Discussion, Page 13-14 |

For peer review only

| Topic | No. | Item | Location where item is reported |
|---|---|---|---|
| Other relevant information | | | |
| **Source of funding** | 27 | Describe how the study was funded and any role of the funder in the identification, design, conduct, and reporting of the analysis | Funding statement and acknowledgments, Page 15 |
| **Conflicts of interest** | 28 | Report authors conflicts of interest according to journal or International Committee of Medical Journal Editors requirements. | Competing interests, Page 15 |

*From:* Husereau D, Drummond M, Augustovski F, et al. Consolidated Health Economic Evaluation Reporting Standards 2022 (CHEERS 2022) Explanation and Elaboration: A Report of the ISPOR CHEERS II Good Practices Task Force. Value Health 2022;25. doi:10.1016/j.jval.2021.10.008

For peer review only

BMJ Open

# Primary care system-level training and support programme for the secondary prevention of domestic violence and abuse: a cost-effectiveness feasibility model

| | |
|---|---|
| Journal: | *BMJ Open* |
| Manuscript ID | bmjopen-2022-071300.R2 |
| Article Type: | Original research |
| Date Submitted by the Author: | 24-Oct-2023 |
| Complete List of Authors: | Cochrane, Madeleine; University of Bristol,<br>Szilassy, Eszter; University of Bristol<br>Coope, Caroline; University of Bristol<br>Emsley, Elizabeth; University of Bristol<br>Johnson, Medina; IRISi<br>Feder, Gene; University of Bristol<br>Barbosa, Estela; University of Bristol |
| <b>Primary Subject Heading</b>: | Health economics |
| Secondary Subject Heading: | Public health |
| Keywords: | PRIMARY CARE, HEALTH ECONOMICS, PUBLIC HEALTH |
| | |

SCHOLARONE™
Manuscripts

*I, the Submitting Author has the right to grant and does grant on behalf of all authors of the Work (as defined in the below author licence), an exclusive licence and/or a non-exclusive licence for contributions from authors who are: i) UK Crown employees; ii) where BMJ has agreed a CC-BY licence shall apply, and/or iii) in accordance with the terms applicable for US Federal Government officers or employees acting as part of their official duties; on a worldwide, perpetual, irrevocable, royalty-free basis to BMJ Publishing Group Ltd ("BMJ") its licensees and where the relevant Journal is co-owned by BMJ to the co-owners of the Journal, to publish the Work in this journal and any other BMJ products and to exploit all rights, as set out in our licence.*

*The Submitting Author accepts and understands that any supply made under these terms is made by BMJ to the Submitting Author unless you are acting as an employee on behalf of your employer or a postgraduate student of an affiliated institution which is paying any applicable article publishing charge ("APC") for Open Access articles. Where the Submitting Author wishes to make the Work available on an Open Access basis (and intends to pay the relevant APC), the terms of reuse of such Open Access shall be governed by a Creative Commons licence – details of these licences and which Creative Commons licence will apply to this Work are set out in our licence referred to above.*

*Other than as permitted in any relevant BMJ Author's Self Archiving Policies, I confirm this Work has not been accepted for publication elsewhere, is not being considered for publication elsewhere and does not duplicate material already published. I confirm all authors consent to publication of this Work and authorise the granting of this licence.*

For peer review only

**Title Page**

# Primary care system-level training and support programme for the secondary prevention of domestic violence and abuse: a cost-effectiveness feasibility model

Madeleine Cochrane, Eszter Szilassy, Caroline Coope, Elizabeth Emsley, Medina Johnson, Gene Feder and Estela Capelas Barbosa

## Correspondence

*Corresponding author:*

Madeleine Cochrane: madeleine.cochrane@bristol.ac.uk

Population Health Sciences, Bristol Medical School, University of Bristol, 1-5 Whiteladies Road, Bristol, BS8 1NU, UK. Tel: 0117 428 3116

*Co-authors:*

Eszter Szilassy: eszter.szilassy@bristol.ac.uk

Caroline Coope: caroline.coope@bristol.ac.uk

Elizabeth Emsley: elizabeth.emsley@bristol.ac.uk

Medina Johnson: medina.johnson@irisi.org

Gene Feder: gene.feder@bristol.ac.uk

Estela Capelas Barbosa: e.capelasbarbosa@bristol.ac.uk

Population Health Sciences, Bristol Medical School, University of Bristol, 1-5 Whiteladies Road, Bristol, BS8 1NU, UK. Eszter Szilassy, Caroline Coope, Elizabeth Emsley, Gene Feder, Estela Capelas Barbosa

IRISi, London, UK. Medina Johnson.



For peer review only

For peer review only - http://bmjopen.bmj.com/site/about/guidelines.xhtml

**Keywords**

Domestic violence, training programme, general practice, primary care, cost-effectiveness.

For peer review only



For peer review only - http://bmjopen.bmj.com/site/about/guidelines.xhtml

**Abstract**

**Objectives:** To evaluate the prospective cost-effectiveness of the Identification and Referral to Improve Safety plus (IRIS+) intervention compared to usual care using feasibility data derived from seven UK GP practice sites.

**Method:** A cost-utility analysis was conducted to assess the potential cost-effectiveness of IRIS+, an enhanced model of the UK's usual care. IRIS+ assisted primary care staff in identifying, documenting and referring not only women, but also men and children who may have experienced domestic violence/ abuse as victims, perpetrators, or both. A perpetrator group programme was not part of the intervention per se, but was linked to the IRIS+ intervention via a referral pathway and signposting. A Markov model was constructed from a societal perspective to estimate mean incremental costs and quality-adjusted life-years (QALYs) of IRIS+ compared to usual care over a 10-year time horizon.

**Results:** The IRIS+ intervention saved £92 per patient and produced QALY gains of 0.003. The incremental net monetary benefit was positive (£145) and the IRIS+ intervention was cost-effective in 55% of simulations at a cost-effectiveness threshold of £20,000 per QALY.

**Conclusion:** The IRIS+ intervention could be cost-effective or even cost-saving from a societal perspective in the UK, though there are large uncertainties, reflected in the confidence intervals and simulation results.



For peer review only - http://bmjopen.bmj.com/site/about/guidelines.xhtml

**Strengths and Limitations**

- To the authors knowledge this is the first study to assess the potential cost-effectiveness of a primary care intervention providing support to all women, men and their children experiencing domestic violence/ abuse
- The study draws on the structure of a previous domestic violence/ abuse model which has been published in peer-reviewed journals
- The study relies on newly collected data, reducing the need for using out-of-date previously published estimates
- The small number of newly collected data means our results may not be representative of the wider UK population

For peer review only



For peer review only - http://bmjopen.bmj.com/site/about/guidelines.xhtml

## Background

Domestic violence/ abuse (DVA) is a public health challenge, affecting approximately 9 million adults and 2 million children in the UK (1-4). The societal cost of DVA was estimated by the UK Home Office to be £66bn in 2017, not including costs to children. Safe Lives, a UK-wide DVA charity, highlighted the need for an initial £2.2bn of public investment per annum to cover domestic abuse services for the whole family– adult, teen and child victims, and perpetrators (5, 6). Public Health England identified primary care as a key location for interventions to prevent DVA and improve health outcomes for adults and children. Early intervention in DVA, for example, in the primary care setting, reduces the overall public service burden of abuse and can reduce escalation of violence (7).

DVA interventions to date have prioritised women, who are disproportionately affected in prevalence and severity of DVA, compared with other groups (8, 9). Identifying female survivors in primary care and referring to specialist support is effective and cost-effective through the provision of DVA training linked with a direct pathway to local DVA support (10). The leading service model in the UK's National Health Service (NHS) primary care setting is IRIS (Identification and Referral to Improve Safety), a widely commissioned evidence-based DVA training and advocacy support programme for female survivors.

While there is growing success in identifying women affected by DVA, male survivors and children/ young people (CYP) are rarely identified in primary care and referred for specialist support. This neglects the mental and physical health impact across the life-course for CYP who experience or witness DVA (11, 12) and the significant mental health impact on men exposed to DVA (13-16). IRIS plus (IRIS+) was an enhanced model of the existing IRIS programme and was piloted in NHS primary care GP sites, three sites in England and four sites in Wales. IRIS+ assisted GP practice staff in identifying, documenting and referring not only

women, but also men and children who may have experienced DVA as victims, perpetrators, or both. The IRIS+ pilot study showed feasibility and acceptability of the intervention to clinicians and those affected by DVA (17, 18). The aim of this study was to evaluate the prospective cost-effectiveness of the IRIS+ intervention when compared to usual care (the IRIS intervention). This study addresses a gap in the literature around the possible cost-effectiveness of interventions targeting men and children as well as women experiencing DVA.

## Methods

*Overview of economic evaluation*

This study was a model-based cost–utility analysis, comparing the IRIS+ intervention to usual care (the IRIS intervention). An unpublished health economic analysis plan (HEAP) was developed prospectively to guide the economic evaluation. The outcome measure was quality-adjusted life years (QALYs), which is the recommended outcome for economic evaluations in the UK (19). As many of the costs of DVA are borne outside the health system, the analysis was undertaken from a UK societal perspective which in this study we define as the costs associated with implementing the intervention, downstream multi-sector costs associated with DVA, as well as productivity costs. Costs relating to DVA perpetration were included in the cost of onward referral, given that a perpetrator programme was linked to IRIS+ via an onward referral pathway or signposting. Costs were calculated in 2019/20 UK£, as most of the IRIS+ intervention took place in those years. Costs and benefits were calculated over a 10-year time horizon. This was considered appropriate because the occurrence of new cases and transition probabilities were assumed to remain constant over time and therefore the impact of a longer time horizon would be small. While this is likely to be the case for adults, we acknowledge that the time horizon for children may be longer (20). This means we opted for a conservative estimate of the cost-effectiveness of the intervention as far as children are concerned. Future



For peer review only - http://bmjopen.bmj.com/site/about/guidelines.xhtml

costs and outcomes were discounted at an annual rate of 3.5% as recommended in the UK guidelines for conducting economic evaluations (19) .

*Model structure*

We developed a Markov model based on the previous analysis of the cost-effectiveness of the usual care intervention (IRIS) (21, 22). The model has five health states (see Figure 1 for details) and the cycle length was six months, which reflects the average length of support received from advocacy services following referral. The cycle length of six months also reflects the maximum time of support available for identified patients. Other than death, which is an absorbing state, men, women and children can transition between states in half-yearly cycles. The states were 'No abuse', 'Abuse not identified', 'Abuse identified and seeing advocate', 'Abuse identified, not seeing advocate' and 'Dead' (Figure 1). A hypothetical cohort of 10,000 people was simulated moving between the states (Figure 1). We used the Census figures to estimate the proportion of adult men, women and children within this hypothetical cohort (14). The model was built and run using Excel VBA.

*Interventions*

*The IRIS intervention (usual care arm)*

The IRIS intervention is a multi-component intervention which has described elsewhere (21, 23). In short, it is delivered in UK NHS primary care GP sites and consists of multidisciplinary training sessions, targeted at the clinical team and some general practice reception staff. The training sessions were designed to address barriers to improving the response of clinicians to women experiencing abuse through improved identification, support and referral to specialist agencies. Clinicians are trained to have a low threshold for asking about DVA. Training incorporates case studies and practice in asking about violence and



For peer review only - http://bmjopen.bmj.com/site/about/guidelines.xhtml

responding appropriately. They are delivered by an advocate educator from collaborating specialist support services. The advocate educator is central to the IRIS intervention, combining a training and support role to the practices with provision of advocacy to women referred. Ongoing support to clinicians and reception staff in the practices is provided by the advocate educator.

*The IRIS+ intervention (intervention arm)*

The IRIS+ intervention builds on the IRIS model, but in addition provides a service for men and children. Similar to IRIS, it consists of a multi-component intervention, including multidisciplinary training for clinicians and general practice staff. IRIS+ provides a simple pathway of referrals to specialist support services for women, men and their children who experience (survivors and perpetrators) DVA. In IRIS+, as well as the advocate educator, there is a dedicated children's worker. Jointly they support any referral made by clinicians, regardless of gender or age. While perpetrators could have been identified by the IRIS+ intervention, a perpetrator group programme was not part of the intervention per se, but was linked to the IRIS+ intervention via a referral pathway and signposting.. Perpetrators could also self-refer into the perpetrator programme.

*Comparisons between IRIS+ (intervention arm) and IRIS (usual care arm)*

Given that this study was a pilot, we did not recruit practices into the usual care arm (IRIS). In fact, the recruitment for IRIS+ included seven practices, three non-IRIS trained practices that had not previously received IRIS or practice-based DVA interventions, and four IRIS-trained practices that had previously received IRIS training. The comparison between IRIS (usual care) and IRIS+ used estimated parameters based on the same areas, given both IRIS and IRIS+ programmes were available for this subset of practices.



For peer review only - http://bmjopen.bmj.com/site/about/guidelines.xhtml

*Parameters*

Whenever possible, we used data collected in the pilot to estimate transition probabilities, utilities and costs required for the Markov model. Where this was not possible probabilities were obtained from published sources. Table 1 shows the source of data for each relevant parameter. Tables S1 and S2 report the same parameters however, they are reported in separate tables for adults and children, respectively.

For peer review only



For peer review only - http://bmjopen.bmj.com/site/about/guidelines.xhtml

**Table 1. Model input parameters: probabilities**

| Parameter | Base case value | Lower limit | Upper limit | Distribution | Source |
|---|---|---|---|---|---|
| ***Probabilities*** | | | | | |
| Proportion of people experiencing abuse- *all ages* | 0.055 | 0.038 | 0.106 | Beta | **Adjusted estimate |
| Prevalence of DVA in adults (males and females) – aged 16 to 90+ years | 0.055 | 0.036 | 0.073 | Beta | (14) |
| Prevalence of children exposed to DVA | 0.080 | 0.040 | 0.140 | Beta | (24) |
| *Starting distribution for those experiencing abuse- all ages* | | | | | |
| Abused and identified, seeing advocate | 0.003 | 0 | 0.0066 | Uniform | * |
| Abused and identified, not seeing advocate | 0.033 | 0 | 0.0660 | Uniform | * |
| Abused but not identified | 0.964 | - | - | Uniform | Complement |
| *Transition probabilities- intervention and control* | | | | | |
| Not abused to Abused but not identified | 0.0037 | 0.0004 | 0.0106 | Dirichlet | * |
| Not abused to Dead | 0.0052 | 0.0027 | 0.0087 | Dirichlet | (14) |
| Stay in Not abused | 0.9911 | - | - | Dirichlet | Complement |
| Abused and identified, seeing advocate to Not abused | 0.1408 | 0.0707 | 0.2301 | Dirichlet | (25) |
| Abused and identified, seeing advocate to Dead | 0.0052 | 0.0000 | 0.0299 | Dirichlet | (14) |



For peer review only - http://bmjopen.bmj.com/site/about/guidelines.xhtml

| | | | | | |
|---|---|---|---|---|---|
| Stay in Abused and identified, seeing advocate | 0.854 | - | - | Dirichlet | Complement |
| Abused and identified, not seeing advocate to Not abused | 0.0781 | 0.0136 | 0.1912 | Dirichlet | (25) |
| Abused and identified, not seeing advocate to Dead | 0.0052 | 0.0000 | 0.0424 | Dirichlet | (14) |
| Stay in Abused and identified, not seeing advocate | 0.9167 | - | - | Dirichlet | Complement |
| *Transition probabilities- intervention* | | | | | |
| Abused but not identified to Not abused | 0.0500 | 0.0412 | 0.0596 | Dirichlet | * |
| Abused but not identified to Abused and identified, not seeing advocate | 0.0374 | 0.0298 | 0.0458 | Dirichlet | IRIS+ data |
| Abused but not identified to Abused and identified, seeing advocate | 0.0312 | 0.0243 | 0.0390 | Dirichlet | IRIS+ data |
| Abused but not identified to Dead | 0.0055 | 0.0029 | 0.0091 | Dirichlet | (14) |
| Stay in Abused but not identified | 0.8762 | - | - | Dirichlet | Complement |
| *Transition probabilities- control* | | | | | |
| Abused but not identified to Not abused | 0.0500 | 0.0412 | 0.0596 | Dirichlet | * |
| Abused but not identified to Abused and identified, not seeing advocate | 0.0091 | 0.0055 | 0.0135 | Dirichlet | IRIS data |
| Abused but not identified to Abused and identified, seeing advocate | 0.0226 | 0.0168 | 0.0293 | Dirichlet | IRIS data |
| Abused but not identified to Dead | 0.0055 | 0.0029 | 0.0091 | Dirichlet | (14) |
| Stay in Abused but not identified | 0.9131 | - | - | Dirichlet | Complement |

* Internal calculation based on model calibration **Weighted average for adults and children



For peer review only - http://bmjopen.bmj.com/site/about/guidelines.xhtml

*Prevalence of domestic abuse*

The proportion of adults aged 16 years or older experiencing abuse was estimated from the Crime Survey for England and Wales (CSEW) (14). There was a subsequent published estimate, but due to anti-contagion measures relating to the COVID-19 pandemic, the survey had to be moved to telephone survey, preventing some of the collection of relevant data on domestic violence. In 2018/19, 5.5% of adults experienced some form of domestic violence according to the CSEW (14). Since IRIS+ also provides support services for children, we relied on the published estimate of 8% of children experiencing some form of DVA in the past 12 months (24). Children represent 20% of the UK population (26). To extrapolate beyond age 65 we used data from the USA showing that the prevalence of intimate partner violence was 2.2% among people aged 65 or older (27). We therefore estimate that 5.5% of the UK population would be in any of the three states in the Markov model associated with abuse in the first model cycle.

*Transition probabilities*

Table 1 reports all transition probabilities. There are eight transitions between states in the model, measured as follows:

1. No abuse to Abuse not identified

No data were available to reliably estimate this probability. We thus estimated it using the model calibration method described below.

2. Abuse not identified to Abuse identified and seeing advocate

For those receiving the IRIS+ intervention, we estimated this transition probability based on the number of patients seen by the advocate in IRIS+ pilot. Dividing this number by the total number of eligible patients in the seven GP practices (99337 patients) gives a six-month transition probability. For the usual care practices, this probability was estimated based on the



For peer review only - http://bmjopen.bmj.com/site/about/guidelines.xhtml

number of women aged 16+ registered to GP practices in the same area referred to IRIS advocacy (39382 patients).

3. Abuse not identified to Abuse identified, not seeing advocate

We used the ratio of the number of patients abused and identified vs referred in the IRIS+ intervention to estimate the number of patients abused and identified, not seeing an advocate. These were effectively patients referred who decline support or who could not be contacted following the referral. The transition probability for usual care (IRIS intervention) was calculated as above, but only considered women identified vs referred.

4. Abuse not identified to No abuse

No data was available to reliably estimate this probability. We therefore estimated this using the model calibration method described below.

5. Abuse identified and seeing advocate to No abuse

This was taken from the MOSAIC (mothers' advocates in the community) trial (25), identified in a Cochrane review (28), evaluating the reduction of any type of domestic abuse with any type of advocacy.

6. Abuse identified, not seeing advocate to No abuse

This was taken from the control arm of the MOSAIC trial (25).

7. No abuse to Dead

We relied on the death rate per 1000 as estimated by the Office for National Statistics. For 2019, it was estimated at 10.4 per 1000. This implies the rate of dying per 6 months is 5.2 per 1000 people, excluding domestic homicides.

8. Abused to Dead

For patients experiencing abuse this probability was 5.54 per 1000 (figure including domestic homicides) per 6 months. This estimate uses the Office for National Statistics death rate for 2019, including domestic homicides. For the purposes of the cost-effectiveness model patients



For peer review only

could not transition between the health states 'abuse identified, not seeing advocate' and 'abuse identified and seeing advocate'. This is because advocacy and support was offered to identified patients at point of referral and not re-offered. A patient could in principle self-refer into the support service later. But if a patient self-referred after being identified by GP practice teams within 6 months, this would be considered a repeat referral and excluded from the model.

*Model calibration*

We used the prevalence of abuse (5.5%) to calibrate the model, since there was uncertainty surrounding transition probabilities for 'No abuse to Abuse not identified' and vice versa. The calibration was run for 3000 cycles, assuming that after this, the number of patients in each state would remain constant. The transition probabilities for 'No abuse to Abuse not identified' and vice versa were changed until the proportion of patients in the 'No abuse' state exactly reflected the observed prevalence (100-5.5=94.5%). The initial steady state calculation showed that that the probabilities from 'No abuse to Abuse not identified' and 'Abuse not identified to No abuse' needed adjusting. We then re-ran the calibration process using a prevalence of abuse figure of 17% , estimated in Richardson and colleagues' study(29). This led to an increase in the probability of 'Abuse not identified to No abuse' from 0.005 to 0.033, which is in line with the finding that prevalence of abuse identified at general practice is higher than in the general population (30). We assessed whether this increase significantly changed the results from the model in a univariate sensitivity analysis and concluded that it did not change the results significantly, although it contributed to its uncertainty. To compensate for this increase, we increase the probability of 'No abuse to Abuse not identified' from 0.0027 to 0.0033. These adjustments meant that the model better reflected the population prevalence of abuse. The initial distribution of patients in the relevant states were 94.5% in 'No abuse', 5.3% in 'Abuse



For peer review only - http://bmjopen.bmj.com/site/about/guidelines.xhtml

For peer review only

not identified’, 0.018% in ‘Abuse identified and seeing advocate’ and 0.18% in ‘Abuse identified, not seeing advocate’.

*Utilities*

Each state in the Markov model was associated with a utility score (Table 2), allowing us to measure QALYs associated with IRIS+ and usual care (IRIS) based on the proportion of patients in each health state in each of the 20 cycles in the model. Utility scores were separately collected and calculated for men, women, and children. For the health state ‘No abuse’ the utility was assumed to be 0.85 for adults and 0.95 for children, following published population norms (31). A subset of adults and children identified from the IRIS+ intervention filled in a SF-12 and CHU-9D form, respectively. If support/ advocacy was accepted, questionnaire data were requested at: (1) baseline, defined as when support/ advocacy started; and (2) between 6-10 months follow up, defined as the period when support/ advocacy ended. A validated mapping algorithm was used to transform SF-12 scores to SF-6D utilities (32). The published SF-6D utilities were derived from a representative sample (n=611) of the UK adult population using the standard gamble valuation method. Similarly, a published value set was used to transform CHU-9D scores into utilities (33). The published CHU-9D value set was derived from members of the UK adult population (n=300) using both standard gamble and ranking valuation methods. Estimated scores at baseline were attributed to ‘Abuse identified, not seeing advocate’. Follow up scores were attributed to ‘Abuse identified and seeing advocate’. Due to the small number of forms collected (n=30 at baseline; n=16 at follow up), this data was compared with previous literature for women for sense checking (34). For ‘Abuse not identified’, we assumed the utility score was the same as ‘Abuse identified, not seeing advocate’, based on the assumption that identification alone (without advocacy support) does not improve quality of life.



For peer review only - http://bmjopen.bmj.com/site/about/guidelines.xhtml

*Costs*

We included: intervention costs; costs of onward referral; and costs associated with abuse (including costs to the UK NHS, costs of lost economic output, costs to the criminal/ civil justice system, personal costs) (Table 2). Intervention costs were taken from the budget of the programme. The total budget for the delivery of IRIS+ was £60,253 and included salaries of the advocate educator and children worker, travel and consumables. This was divided by the total patient population exposed to the intervention (79485 patients). The cost of onward referral considered the time an advocate educator or a children worker may spend working with external agencies (on average 57 hours), where their support alone would not suffice, multiplied by their average hourly salary (£29.60), and by 39%, which was the proportion of patients referred to the advocate or children's worker who accepted support and needed to be referred to another agency (57 x £29.60 x 0.39 = £658). The cost of onward referral included the cost of referring men to the perpetrator programme. IRIS+ identified five men perpetrators, of which three engaged with the advocate educator. Of these, two accepted an onward referral to a perpetrator programme after risk assessment.

Costs associated with domestic violence in the UK for people aged 16+ is described in Oliver et al (35). In this report, costs of lost economic output, health services, criminal justice system, civil justice system, social welfare, personal costs, specialised services and physical/emotional harm were included, and unit cost per victim per year is estimated at £34,015 (in 2019 prices). We excluded costs of physical/ emotional harm (£24,300), because in its report, Oliver et al calculate cost of physical/ emotional harm by monetising QALY detriments. Since QALY gains are estimated for the intervention, including monetised QALY detriments in our costs was deemed inappropriate. This, however, implies that our results are conservative. For adults,



For peer review only - http://bmjopen.bmj.com/site/about/guidelines.xhtml

the cost of abuse per 6 months was £4,858. For children, we relied on a report produced by Pro Bono Economics (20), which estimated the cost of domestic violence per child to be £1,950 per 6 months in 2018£. We inflated this estimate (£1,969 in 2019£). We considered children to account for 20% of the UK population and estimated an overall cost of abuse per victim of £4,276 (£4858 x 0.8 – adults + £1969 x 0.2 – children) per 6 months.

For peer review only



For peer review only - http://bmjopen.bmj.com/site/about/guidelines.xhtml

**Table 2. Model input parameters: utilities and costs**

| Parameter | Base case value | Lower limit | Upper limit | Distribution | Source |
|---|---|---|---|---|---|
| ***Utilities*** | | | | | |
| Not abused (adults) | 0.850 | 0.840 | 0.860 | Beta | (31) |
| Not abused (children) | 0.950 | 0.940 | 0.959 | Beta | (31) |
| Abused but not identified (women) | 0.656 | 0.522 | 0.749 | Beta | Assumption |
| Abused but not identified (men) | 0.626 | 0.500 | 0.744 | Beta | Assumption |
| Abused but not identified (children) | 0.801 | 0.623 | 0.932 | Beta | Assumption |
| Abused and identified, seeing advocate (women) | 0.659 | 0.518 | 0.782 | Beta | IRIS+ data |
| Abused and identified, seeing advocate (men) | 0.701 | 0.555 | 0.828 | Beta | IRIS+ data |
| Abused and identified, seeing advocate (children) | 0.804 | 0.625 | 0.935 | Beta | IRIS+ data |
| Abused and identified, not seeing advocate (women) | 0.656 | 0.522 | 0.749 | Beta | IRIS+ data |
| Abused and identified, not seeing advocate (men) | 0.626 | 0.500 | 0.744 | Beta | IRIS+ data |
| Abused and identified, not seeing advocate (children) | 0.801 | 0.623 | 0.932 | Beta | IRIS+ data |
| ***Costs (2019/20£)*** | | | | | |
| Costs of the intervention, per patient exposed to DV, per 6 months | £0.75 | £0.02 | £2.73 | Gamma | IRIS+ budget |



For peer review only

| | | | | | |
|---|---|---|---|---|---|
| Cost of onward referral, once | £658 | £11 | £1908 | Gamma | IRIS+ data and IRIS data |
| Cost of Abused but not identified (weighted average – exposed population) | £4276 | £108 | £15774 | Gamma | **weighted average |
| Cost of Abused but not identified (adults) | £4858 | £123 | £17919 | Gamma | (35) |
| Cost of Abused but not identified (children) | £1950 | £1000 | £2500 | Gamma | (20) |
| Weighted costs Abused and identified, seeing advocate | 1 | 0.75 | 1.25 | Gamma | Assumption |
| Weighted costs Abused and identified, not seeing advocate | 1 | 0.9 | 1.1 | Gamma | Assumption |

Costs are in 2019/20 UK£. **Excludes the cost of harms, which in this modelled are measured as benefits

For peer review only



For peer review only - http://bmjopen.bmj.com/site/about/guidelines.xhtml

*Cost-utility analysis*

A cost-utility analysis was conducted comparing costs and QALYs for IRIS+ versus IRIS (usual care). QALYs were calculated from utilities by using the area under the curve approach. The main outcome was the Net Monetary Benefit (NMB), that estimates both costs and QALYs in monetary terms, using an acceptability threshold of £20,000 per QALY. A positive incremental NMB result indicates that IRIS+ intervention would be preferred on cost-effectiveness grounds. While a negative incremental NMB result indicates that the IRIS intervention (usual care) would be preferred. Results were also shown in terms of the incremental costs per QALY gained of IRIS+ vs IRIS. This was measured as the mean difference in costs between IRIS+ and IRIS divided by the mean difference in QALYs. We followed the usual decision making rule for cost-effectiveness in the UK, in which an intervention is likely to be considered cost-effective when the incremental costs per QALY gained are less than £20,000 (19).

*Subgroups and distributional effects*

The IRIS+ and IRIS arms represented two key groups which could be targeted in primary care (women, men and their children vs women only). Consequently, we did not estimate cost-effectiveness for any alternative subgroups. DVA is experienced across all social groups including all different socioeconomic, ethnicity and geographical groups. The IRIS and IRIS+ interventions are designed for all social groups, therefore we did not consider distributional effects.

*Sensitivity analysis*



For peer review only - http://bmjopen.bmj.com/site/about/guidelines.xhtml

We undertook a probabilistic sensitivity analysis, based on 1000 simulations drawn from random samples from the probability distributions of all parameters. These 1000 simulations were plotted in a cost-effectiveness plane. The proportion of simulations with an incremental cost per QALY gained below the cost-effectiveness threshold was calculated for different threshold values, ranging from £0 to £50,000. The results were presented in a cost-effectiveness acceptability curve.

*Patient and public involvement*

Three patient and public involvement (PPI) groups (female survivors, male survivors and male perpetrators) were created and consulted throughout the lifetime of the research programme. PPI representatives were involved in the development of the IRIS+ intervention and the design of the research study.

## Results

*Base case*

The results of the cost-utility in the base case analysis are presented in Table 3. Over the 10-year time horizon, mean total costs per patient registered at general practices eligible to the IRIS+ intervention were £3,867. For the IRIS intervention (usual care), the mean cost per patient was £3,959. IRIS+ therefore could potentially save £92 per patient over a 10-year time horizon. While a small sample may have contributed to the uncommon finding that the mean total costs in the intervention arm is smaller than the usual care, we identified that this difference is mainly a result of the number of patients that ultimately transition from 'Abuse identified and seeing advocate to No abuse' and 'Abuse identified, not seeing advocate to No abuse'. Given the IRIS+ intervention identifies (and supports) a larger proportion of patients than the control (see Table 1), in our hypothetical cohort of 10,000, at the end of 20 6-monthly



For peer review only - http://bmjopen.bmj.com/site/about/guidelines.xhtml

cycles, there are 8,569 people in the 'Not Abused' health state in the intervention (IRIS+) arm and only 8,538 in the usual care arm, thus preventing some cost of abuse in the IRIS+ intervention arm.

Total QALYs per patient were also 0.003 higher in the IRIS+ arm (7.000) than in the IRIS arm (usual care) (6.997). As the IRIS+ intervention arm was associated with lower costs and higher effectiveness then the incremental cost per QALY gained was negative (dominating usual care, IRIS) and the incremental NMB was positive (£145).

For peer review only



For peer review only - http://bmjopen.bmj.com/site/about/guidelines.xhtml

**Table 3. Discounted base case and probabilistic results**

| | | | |
|---|---|---|---|
| Discounted base case results | | | |
| | **Costs** | **QALYs** | **Cost-effectiveness** |
| Intervention (IRIS+ programme) | £3,867 | 7.000 | |
| Control (IRIS programme) | £3,959 | 6.997 | |
| Difference (intervention vs. control) | -£92 | 0.003 | intervention dominates control |
| Incremental NMB* | | | £145 |
| Probabilistic results | | | |
| | **Costs (95% CI)** | **QALYs (95% CI)** | **Cost-effectiveness (95% CI)** |
| Intervention (IRIS+ programme) | £107 to £16616 | 6.377 to 7.192 | |
| Control (IRIS programme) | £104 to £17343 | 6.377 to 7.197 | |
| Increment | £-1123 to £171 | -0.030 to 0.019 | |
| ICER | | | £-206828 to £277989 |

QALY = quality-adjusted life year. NMB = net monetary benefit. ICER= incremental cost effectiveness ratio. Costs are in 2019/20 UK£. Numbers may not sum due to rounding. *Measured at a willingness to pay for a QALY of £20 000.



For peer review only - http://bmjopen.bmj.com/site/about/guidelines.xhtml

*Sensitivity analysis*

Incremental costs and QALYs varied widely in the probabilistic sensitivity analyses. The 95% confidence interval for incremental costs was -£1,123 to £171, while for incremental QALYs it was -0.030 to 0.019, and the ICER was -£206,828 to £277,989 per QALY gained. Figure 2(a) shows a scatter plot of the incremental costs and incremental QALYs from the 1000 simulations. It shows how much uncertainty there is around these results. The IRIS+ intervention was cost-effective in 55% of simulations when the cost-effectiveness threshold was £20,000 (Figure 2(b)).

## Discussion

We found that the IRIS+ intervention could be cost-effective or even cost-saving from a societal perspective in the UK with a willingness-to-pay threshold of £20,000 per gain in QALY, when compared to usual care (IRIS). There is considerable uncertainty surrounding these results, and only slightly more than a 50% probability that IRIS+ is cost-effective at £20,000 per QALY, the cost-effectiveness threshold commonly used in the UK.

There are a number of strengths and limitations to this study. The main strength relates to this study drawing on newly collected data, reducing the need for using out-of-date previously published estimates. It, however, relies on a small number of observations (n=30 at baseline; n=16 at follow up), which could potentially be unreliable. The large uncertainty in our results reflects the small sample size. Nevertheless, as far as the authors are aware, this is the first study to assess the potential cost-effectiveness of a primary care intervention providing support to not just women, but also men and children experiencing DVA. Another important limitation of this study relates to its prospective nature. Given the pilot design, we were unable to directly recruit practices into IRIS+ and IRIS (usual care). Thus, by using practices in the same area,



For peer review only - http://bmjopen.bmj.com/site/about/guidelines.xhtml

spillover effects may be significant (although they were not explored in this paper). Furthermore, the small number of practices, and as a result the small number of patients identified, meant that subgroup analysis was not possible. A cluster randomised control trial (RCT) comparing IRIS+ to IRIS (usual care) could potentially address some of the uncertainties observed in the cost-effectiveness result of this study. More specifically, an economic evidence collected alongside a trial may shed light onto some of the differences in terms of costs and benefits for women, men and children.

Comparing this prospective study to similar studies in the literature is challenging. Most training and advocacy programmes evaluated to date, have focused on a subset of the population, such as women, children or men only. Including all groups is a key strength of the IRIS+ intervention, as reported in the qualitative findings of this research study (36). Future research should attempt to replicate the intervention in a greater number of general practices across the UK to enable more robust data collection and larger sample sizes.

**Contribution statement**

Cochrane attests that all listed authors meet authorship criteria and that no others meeting the criteria have been omitted from the opportunity to be listed as an author.MC, ES, CC, EE, MJ, GF and EB contributed to the planning of the study. ES and CC managed the coordination of the study. MC and EB conducted the analysis for the study and MC, EB, ES and EE, GF contributed to the interpretation of the data. MC and EB developed the manuscript. MC, ES, CC, EE, MJ, GF and EB read and commented on the manuscript drafts and approved the final manuscript. GF was the Chief Investigator of the study. The study was funded by National Institute for Health Research (Programme Grants for Applied Research), (RP-PG-0614-20012).



For peer review only - http://bmjopen.bmj.com/site/about/guidelines.xhtml

**Competing interests**

Medina Johnson is the CEO of IRISi and was a named partner in REPROVIDE. She did not influence the economic modelling or its results.

**Funding**

IRIS+ is part of the REPROVIDE programme (Reaching Everyone Programme of Research On Violence in diverse Domestic Environments), an independent research programme funded by the National Institute for Health Research (Programme Grants for Applied Research), (RP-PG-0614-20012). The views expressed in this publication are those of the authors and not necessarily those of the National Institute for Health Research or the Department of Health and Social Care. The funder had no role in the design and conduct of the study; collection, management, analysis, and interpretation of the data; preparation, review, or approval of the manuscript; and decision to submit the manuscript for publication.

**Data sharing and data availability statement**

All data requests should be submitted to the corresponding author for consideration. Access to anonymised data may be granted following review.

**Ethics approval statement**

The study was given favourable ethical approval by London - Hampstead Research Ethics Committee (REC reference: 19/LO/1132) and the Health Research Authority (HRA) and Health and Care Research Wales (HCRW).



For peer review only - http://bmjopen.bmj.com/site/about/guidelines.xhtml

## References

1. Alliance VP. The public health approach. World Health Organisation (https://www who int/violenceprevention/approach/public_health/en/) Accessed. 2020;9.
2. Chandan JS, Taylor J, Bradbury-Jones C, Nirantharakumar K, Kane E, Bandyopadhyay S. COVID-19: a public health approach to manage domestic violence is needed. The Lancet Public Health. 2020;5(6):e309.
3. WHO. Violence against women: A global health problem of epidemic proportions Geneva: World Health Organization; 2013 [Available from: http://www.who.int/mediacentre/news/releases/2013/violence_against_women_20130620/en/.
4. Rakovec-Felser Z. Domestic violence and abuse in intimate relationship from public health perspective. Health psychology research. 2014;2(3).
5. Oliver R, Alexander, B., Roe, S., Wlasny, M. The economic and social costs of domestic abuse. London; 2019.
6. SafeLives. A Safe Fund: costing domestic abuse provision for the whole family: SafeLives; 2020 [Available from: https://safelives.org.uk/sites/default/files/A%20Safe%20Fund%20costing%20domestic%20abuse%20provision%20for%20the%20whole%20family%20in%20England%20and%20Wales_0.pdf.
7. Cleaver K, Maras P, Oram C, McCallum K. A review of UK based multi-agency approaches to early intervention in domestic abuse: Lessons to be learnt from existing evaluation studies. Aggression and violent behavior. 2019;46:140-55.
8. Hamberger LK, Larsen SE. Men's and women's experience of intimate partner violence: A review of ten years of comparative studies in clinical samples; Part I. Journal of Family Violence. 2015;30(6):699-717.
9. Walby S, Towers J, Francis B. Is violent crime increasing or decreasing? A new methodology to measure repeat attacks making visible the significance of gender and domestic relations. British Journal of Criminology. 2016;56(6):1203-34.
10. Feder G, Davies RA, Baird K, Dunne D, Eldridge S, Griffiths C, et al. Identification and Referral to Improve Safety (IRIS) of women experiencing domestic violence with a primary care training and support programme: a cluster randomised controlled trial. The Lancet. 2011;378(9805):1788-95.
11. Holt S, Buckley H, Whelan S. The impact of exposure to domestic violence on children and young people: A review of the literature. Child abuse & neglect. 2008;32(8):797-810.
12. McTavish JR, MacGregor JC, Wathen CN, MacMillan HL. Children's exposure to intimate partner violence: An overview. International review of psychiatry. 2016;28(5):504-18.
13. Coker AL, Davis KE, Arias I, Desai S, Sanderson M, Brandt HM, et al. Physical and mental health effects of intimate partner violence for men and women. American journal of preventive medicine. 2002;23(4):260-8.
14. ONS. Domestic abuse victim characteristics, England and Wales: year ending March 2020. Characteristics of victims of domestic abuse based on findings from the Crime Survey for England and Wales and police recorded crime. 2020 12th May 2022.



For peer review only - http://bmjopen.bmj.com/site/about/guidelines.xhtml

15. Hester M, Ferrari G, Jones SK, Williamson E, Bacchus LJ, Peters TJ, et al. Occurrence and impact of negative behaviour, including domestic violence and abuse, in men attending UK primary care health clinics: a cross-sectional survey. 2015;5(5).
16. Hester M, Williamson, E., Regan, L., Coulter, M., Chantler, K., Gangoli, G., Davenport, R., Green, L. . Exploring the service and support needs of male, lesbian, gay, bi-sexual and transgendered and black and other minority ethnic victims of domestic
and sexual violence. Report prepared for Home Office SRG/06/017. Bristol; 2012.
17. Szilassy E, Caroline, C., Roy, J. Elizabeth, E., Gene, F. Feasibility of extending the IRIS domestic violence model 4th European Conference on Domestic Violence; Ljubljana: ECDV; 2021.
18. Roy J, Williamson E, Pitt K, Stanley N, Man MS, Feder G, et al. 'It felt like there was always someone there for us': Supporting children affected by domestic violence and abuse who are identified by general practice. Health & Social Care in the Community. 2022;30(1):165-74.
19. National Institute for Health and Care Excellence. Developing NICE guidelines: the manual. 2022 15th December 2022.
20. Pro Bono Economics. The economic cost from childhood exposure to severe domestic violence. 2018 15th December 2022.
21. Devine A, Spencer A, Eldridge S, Norman R, Feder G. Cost-effectiveness of Identification and Referral to Improve Safety (IRIS), a domestic violence training and support programme for primary care: a modelling study based on a randomised controlled trial. BMJ open. 2012;2(3).
22. Barbosa EC, Verhoef TI, Morris S, Solmi F, Johnson M, Sohal A, et al. Cost-effectiveness of a domestic violence and abuse training and support programme in primary care in the real world: updated modelling based on an MRC phase IV observational pragmatic implementation study. BMJ open. 2018;8(8):e021256.
23. Feder G, Davies RA, Baird K, Dunne D, Eldridge S, Griffiths C, et al. Identification and Referral to Improve Safety (IRIS) of women experiencing domestic violence with a primary care training and support programme: a cluster randomised controlled trial. Lancet (London, England). 2011;378(9805):1788-95.
24. Gilbert R, Widom CS, Browne K, Fergusson D, Webb E, Janson S. Burden and consequences of child maltreatment in high-income countries. The lancet. 2009;373(9657):68-81.
25. Taft AJ, Small R, Hegarty KL, Watson LF, Gold L, Lumley JA. Mothers' AdvocateS In the Community (MOSAIC)--non-professional mentor support to reduce intimate partner violence and depression in mothers: a cluster randomised trial in primary care. BMC public health. 2011;11:178.
26. ONS. Analysis of population estimates tool 2020 [Available from: https://www.ons.gov.uk/peoplepopulationandcommunity/populationandmigration/populationestimates/datasets/analysisofpopulationestimatestool.
27. Bonomi AE, Anderson ML, Reid RJ, Carrell D, Fishman PA, Rivara FP, et al. Intimate partner violence in older women. Gerontologist. 2007;47(1):34-41.
28. Rivas C, Ramsay J, Sadowski L, Davidson LL, Dunne D, Eldridge S, et al. Advocacy interventions to reduce or eliminate violence and promote the physical and psychosocial well-being of women who experience intimate partner abuse. status and date: New search for studies and content updated (no change to conclusions), published in. 2015(12).



For peer review only - http://bmjopen.bmj.com/site/about/guidelines.xhtml

29. Richardson J, Coid J, Petruckevitch A, Chung WS, Moorey S, Feder G. Identifying domestic violence: cross sectional study in primary care. Bmj. 2002;324(7332):274.
30. Feder G, Ramsay J, Dunne D, Rose M, Arsene C, Norman R, et al. How far does screening women for domestic (partner) violence in different health-care settings meet criteria for a screening programme? Systematic reviews of nine UK National Screening Committee criteria: Chapter 4 What is the prevalence of partner violence against women and its impact on health? (Question I). Health Technol Assess. 2009;13(16):iii-iv, xi-xiii, 1-113, 37-347.
31. Kind K, Hardman G, Macran S. UK Population norms for EQ-5D. University of York; 1999.
32. Brazier JE, Roberts J. The estimation of a preference-based measure of health from the SF-12. Medical care. 2004;42(9):851-9.
33. Stevens K. Valuation of the Child Health Utility 9D Index. Pharmacoeconomics. 2012;30(8):729-47.
34. Wittenberg E, Lichter EL, Ganz ML, McCloskey LA. Community preferences for health states associated with intimate partner violence. Medical care. 2006;44(8):738-44.
35. Oliver R, Alexander B, Roe S, Wlasny M. The economic and social costs of domestic abuse. Home Office (UK). 2019.
36. Szilassy E, Roy J, Williamson E, Pitt K, Man M-S, Feder G. Reaching everyone in general practice? Feasibility of an integrated domestic violence training and support intervention in primary care. BMC family practice. 2021;22(1):1-17.

**Figures**

Figure 1. Health states and movement between health states in Markov model.

The model starts with all patients in either the ‘No abuse’ state or one of the states associated with abuse, based on the prevalence of DV (see text). Patients in the ‘No abuse’ state could stay in this state, move to ‘Abuse not identified’ or die from any cause. Once a patient is in the ‘Abuse not unidentified’ state, they could stay in that state, move back to ‘No abuse’, move to ‘Abuse identified and seeing advocate’ or ‘Abuse identified, not seeing advocate’ or die. Patients in the ‘Abuse identified’ states could stay in these states, move back to ‘No abuse’ or die. Death is an absorbing state.



For peer review only - http://bmjopen.bmj.com/site/about/guidelines.xhtml

Figure 2. Probabilistic sensitivity analysis.

(a) Scatter plot of incremental costs and incremental QALYs from 1000 simulations.

(b) Cost-effectiveness acceptability curve showing the probability that the intervention (IRIS+) is cost-effective vs. control (IRIS) at different values of the maximum willingness to pay for a QALY. QALY = quality-adjusted life year. Costs are in 2019/20 UK£.

For peer review only



For peer review only - http://bmjopen.bmj.com/site/about/guidelines.xhtml

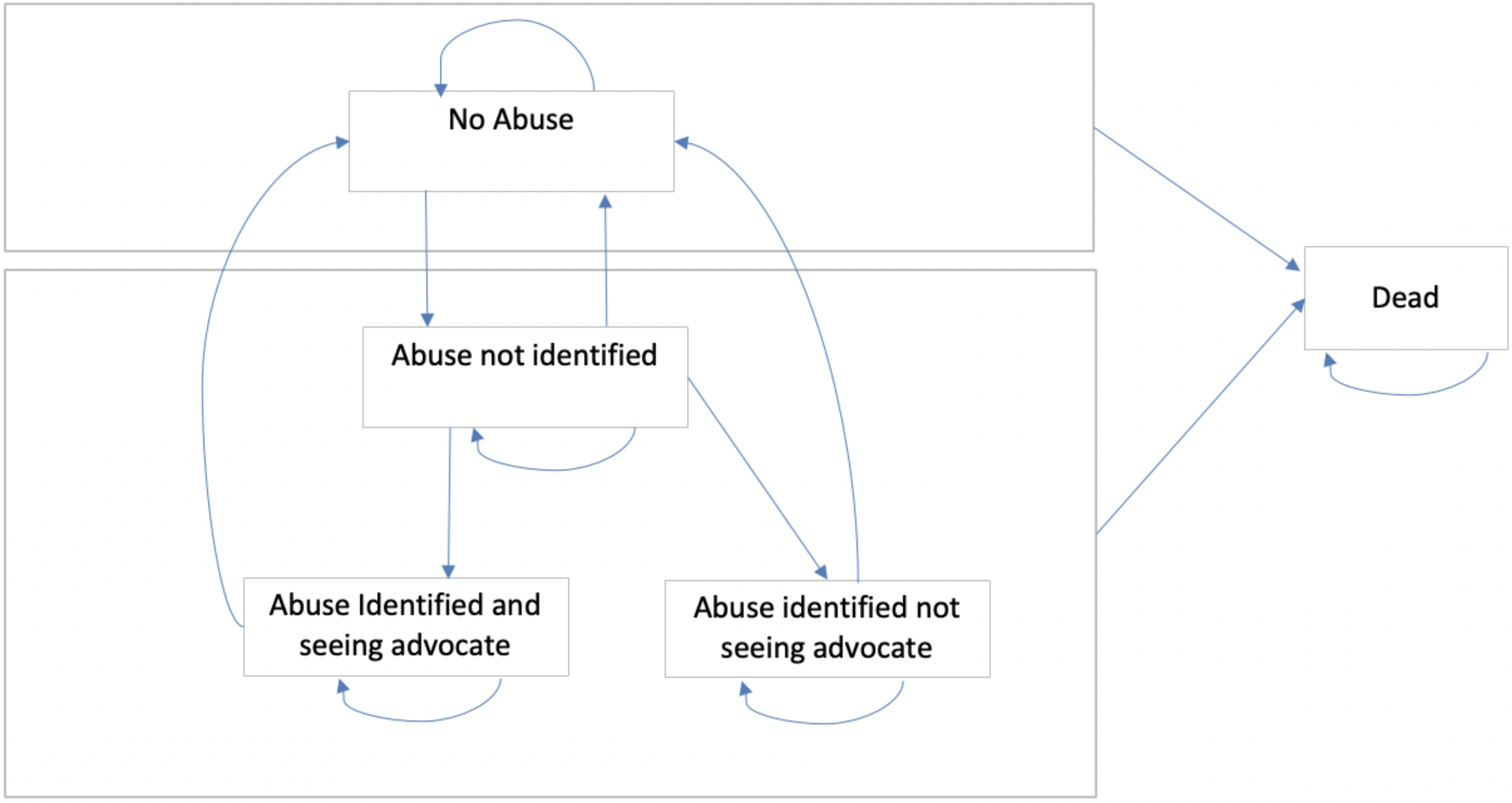
No Abuse
Abuse not identified
Abuse Identified and seeing advocate
Abuse identified not seeing advocate
Dead

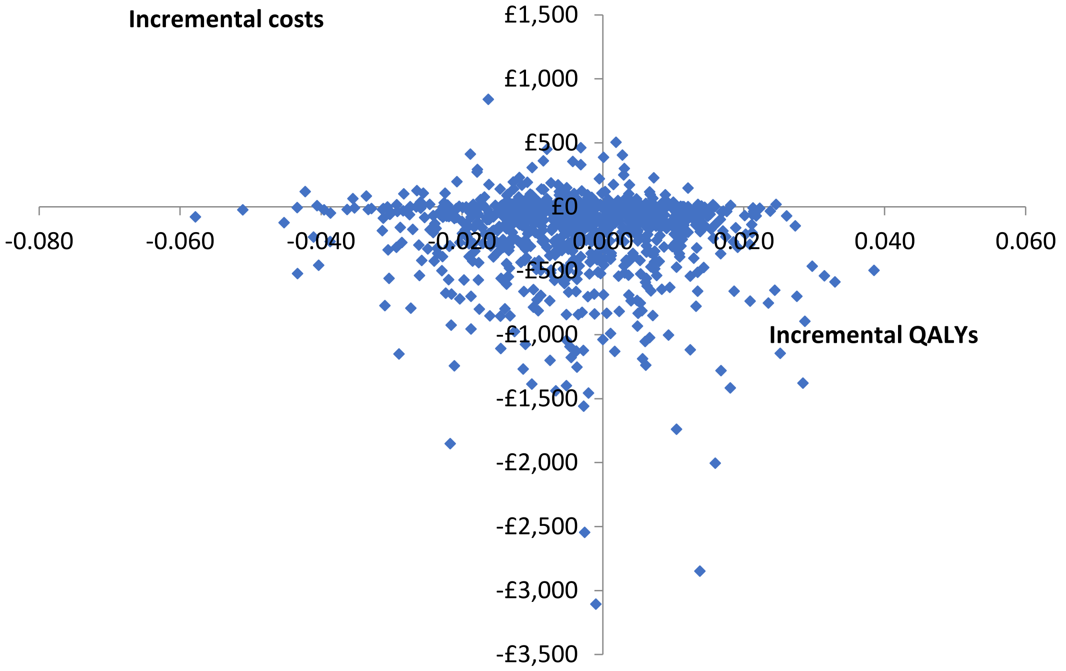

Incremental costs
£1,500
£1,000
£500
£0
-£500
-£1,000
-£1,500
-£2,000
-£2,500
-£3,000
-£3,500
-0.080
-0.060
-0.040
-0.020
0.000
0.020
0.040
0.060
Incremental QALYs


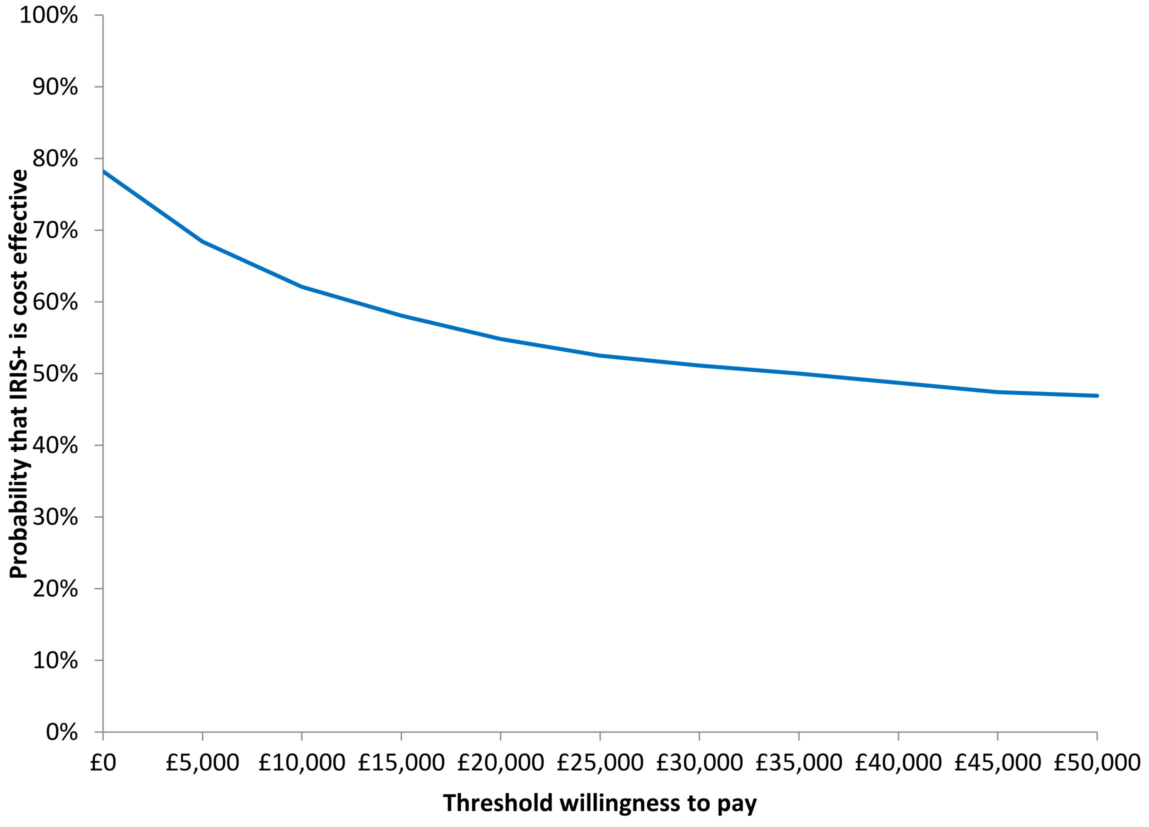

100%
90%
80%
70%
60%
50%
40%
30%
20%
10%
0%
Probability that IRIS+ is cost effective
£0
£5,000
£10,000
£15,000
£20,000
£25,000
£30,000
£35,000
£40,000
£45,000
£50,000
Threshold willingness to pay

**Primary care system-level training and support programme for the secondary prevention of domestic violence and abuse: a cost-effectiveness feasibility model**

Madeleine Cochrane, Eszter Szilassy, Caroline Coope, Elizabeth Emsley, Medina Johnson, Gene Feder and Estela Capelas Barbosa

For peer review only

**Table S1. Adult's model input parameters: probabilities; utilities; and, costs.**

| Parameter | Base case value | Lower limit | Upper limit | Distribution | Source |
|---|---|---|---|---|---|
| ***Probabilities*** | | | | | |
| Prevalence of DVA in adults (males and females) – aged 16 to 65 | 0.055 | 0.036 | 0.073 | Beta | ONS, 2020b |
| *Starting distribution for patients who are abused* | | | | | |
| Abused and identified, seeing advocate | 0.003 | 0 | 0.0066 | Uniform | * |
| Abused and identified, not seeing advocate | 0.033 | 0 | 0.0660 | Uniform | * |
| Abused but not identified | 0.964 | - | - | Uniform | Complement |
| *Transition probabilities* | | | | | |
| Not abused to Abused but not identified | 0.0037 | 0.0004 | 0.0106 | Dirichlet | * |
| Not abused to Dead | 0.0052 | 0.0027 | 0.0087 | Dirichlet | ONS, 2020b |
| Stay in Not abused | 0.9911 | - | - | Dirichlet | Complement |
| Abused but not identified to Not abused (control) | 0.0500 | 0.0412 | 0.0596 | Dirichlet | * |
| Abused but not identified to Abused and identified, not seeing advocate (control) | 0.0091 | 0.0055 | 0.0135 | Dirichlet | IRIS data |

For peer review only

| | | | | | |
|---|---|---|---|---|---|
| Abused but not identified to Abused and identified, seeing advocate (control) | 0.0226 | 0.0168 | 0.0293 | Dirichlet | IRIS data |
| Abused but not identified to Dead (control) | 0.0055 | 0.0029 | 0.0091 | Dirichlet | ONS, 2020b |
| Stay in Abused but not identified (control) | 0.9131 | - | - | Dirichlet | Complement |
| Abused but not identified to Not abused (intervention) | 0.0500 | 0.0412 | 0.0596 | Dirichlet | * |
| Abused but not identified to Abused and identified, not seeing advocate (intervention) | 0.0374 | 0.0298 | 0.0458 | Dirichlet | IRIS+ data |
| Abused but not identified to Abused and identified, seeing advocate (intervention) | 0.0312 | 0.0243 | 0.0390 | Dirichlet | IRIS+ data |
| Abused but not identified to Dead (intervention) | 0.0055 | 0.0029 | 0.0091 | Dirichlet | ONS, 2020B |
| Stay in Abused but not identified (intervention) | 0.8762 | - | - | Dirichlet | Complement |
| Abused and identified, seeing advocate to Not abused | 0.1408 | 0.0707 | 0.2301 | Dirichlet | (Taft et al., 2011) |
| Abused and identified, seeing advocate to Dead | 0.0052 | 0.0000 | 0.0299 | Dirichlet | ONS, 2020b |
| Stay in Abused and identified, seeing advocate | 0.854 | - | - | Dirichlet | Complement |
| Abused and identified, not seeing advocate to Not abused | 0.0781 | 0.0136 | 0.1912 | Dirichlet | (Taft et al., 2011) |
| Abused and identified, not seeing advocate to Dead | 0.0052 | 0.0000 | 0.0424 | Dirichlet | ONS, 2020b |

| | | | | | |
|---|---|---|---|---|---|
| Stay in Abused and identified, not seeing advocate | 0.9167 | - | - | Dirichlet | Complement |
| ***Utilities*** | | | | | |
| Not abused (adults) | 0.850 | 0.840 | 0.860 | Beta | (Kind et al., 1999) |
| Abused but not identified (women) | 0.656 | 0.522 | 0.749 | Beta | Assumption |
| Abused but not identified (men) | 0.626 | 0.500 | 0.744 | Beta | Assumption |
| Abused and identified, seeing advocate (women) | 0.659 | 0.518 | 0.782 | Beta | IRIS+ data |
| Abused and identified, seeing advocate (men) | 0.701 | 0.555 | 0.828 | Beta | IRIS+ data |
| Abused and identified, not seeing advocate (women) | 0.656 | 0.522 | 0.749 | Beta | IRIS+ data |
| Abused and identified, not seeing advocate (men) | 0.626 | 0.500 | 0.744 | Beta | IRIS+ data |
| ***Costs (2019/20£)*** | | | | | |
| Costs of the intervention, per patient exposed to DV, per 6 months | £0.75 | £0.02 | £2.73 | Gamma | IRIS+ budget |
| Cost of onward referral, once | £658 | £11 | £1908 | Gamma | IRIS+ data; IRIS data |
| Cost of Abused but not identified (adults) | £4858 | £123 | £17919 | Gamma | (Oliver et al., 2019) |
| Weighted costs Abused and identified, seeing advocate | 1 | 0.75 | 1.25 | Gamma | Assumption |
| Weighted costs Abused and identified, not seeing advocate | 1 | 0.9 | 1.1 | Gamma | Assumption |

Costs are in 2019/20 UK£.
* Internal calculation based on model calibration.

For peer review only

± Excludes the cost of harms, which in this modelled are measured as benefit

**Table S2. Children's model input parameters: probabilities; utilities; and, costs.**

| Parameter | Base case value | Lower limit | Upper limit | Distribution | Source |
|---|---|---|---|---|---|
| ***Probabilities*** | | | | | |
| Prevalence of children exposed to DVA | 0.080 | 0.040 | 0.140 | Beta | (Gilbert et al., 2009) |
| *Starting distribution for patients who are abused* | | | | | |
| Abused and identified, seeing advocate | 0.003 | 0 | 0.0066 | Uniform | * |
| Abused and identified, not seeing advocate | 0.033 | 0 | 0.0660 | Uniform | * |
| Abused but not identified | 0.964 | - | - | Uniform | Complement |
| *Transition probabilities* | | | | | |
| Not abused to Abused but not identified | 0.0037 | 0.0004 | 0.0106 | Dirichlet | * |
| Not abused to Dead | 0.0052 | 0.0027 | 0.0087 | Dirichlet | ONS, 2020b |
| Stay in Not abused | 0.9911 | - | - | Dirichlet | Complement |
| Abused but not identified to Not abused (control) | 0.0500 | 0.0412 | 0.0596 | Dirichlet | * |

For peer review only

| | | | | | |
|---|---|---|---|---|---|
| Abused but not identified to Abused and identified, not seeing advocate (control) | 0.0091 | 0.0055 | 0.0135 | Dirichlet | IRIS data |
| Abused but not identified to Abused and identified, seeing advocate (control) | 0.0226 | 0.0168 | 0.0293 | Dirichlet | IRIS data |
| Abused but not identified to Dead (control) | 0.0055 | 0.0029 | 0.0091 | Dirichlet | ONS, 2020b |
| Stay in Abused but not identified (control) | 0.9131 | - | - | Dirichlet | Complement |
| Abused but not identified to Not abused (intervention) | 0.0500 | 0.0412 | 0.0596 | Dirichlet | * |
| Abused but not identified to Abused and identified, not seeing advocate (intervention) | 0.0374 | 0.0298 | 0.0458 | Dirichlet | IRIS+ data |
| Abused but not identified to Abused and identified, seeing advocate (intervention) | 0.0312 | 0.0243 | 0.0390 | Dirichlet | IRIS+ data |
| Abused but not identified to Dead (intervention) | 0.0055 | 0.0029 | 0.0091 | Dirichlet | ONS, 2020b |
| Stay in Abused but not identified (intervention) | 0.8762 | - | - | Dirichlet | Complement |
| Abused and identified, seeing advocate to Not abused | 0.1408 | 0.0707 | 0.2301 | Dirichlet | (Taft et al., 2011) |
| Abused and identified, seeing advocate to Dead | 0.0052 | 0.0000 | 0.0299 | Dirichlet | ONS, 2020b |
| Stay in Abused and identified, seeing advocate | 0.854 | - | - | Dirichlet | Complement |

For peer review only

| | | | | | |
|---|---|---|---|---|---|
| Abused and identified, not seeing advocate to Not abused | 0.0781 | 0.0136 | 0.1912 | Dirichlet | (Taft et al., 2011) |
| Abused and identified, not seeing advocate to Dead | 0.0052 | 0.0000 | 0.0424 | Dirichlet | ONS, 2020b |
| Stay in Abused and identified, not seeing advocate | 0.9167 | - | - | Dirichlet | Complement |
| ***Utilities*** | | | | | |
| Not abused (children) | 0.950 | 0.940 | 0.959 | Beta | (Kind et al., 1999) |
| Abused but not identified (children) | 0.801 | 0.623 | 0.932 | Beta | Assumption |
| Abused and identified, seeing advocate (children) | 0.804 | 0.625 | 0.935 | Beta | IRIS+ data |
| Abused and identified, not seeing advocate (children) | 0.801 | 0.623 | 0.932 | Beta | IRIS+ data |
| ***Costs (2019/20£)*** | | | | | |
| Costs of the intervention, per patient exposed to DV, per 6 months | £0.75 | £0.02 | £2.73 | Gamma | IRIS+ budget |
| Cost of onward referral, once | £658 | £11 | £1908 | Gamma | IRIS+ data; IRIS data |
| Cost of Abused but not identified (children) | £1950 | £1000 | £2500 | Gamma | (Pro Bono Economics, 2018) |
| Weighted costs Abused and identified, seeing advocate | 1 | 0.75 | 1.25 | Gamma | Assumption |
| Weighted costs Abused and identified, not seeing advocate | 1 | 0.9 | 1.1 | Gamma | Assumption |

Costs are in 2019/20 UK£. * Internal calculation based on model calibration. ± Excludes the cost of harms, which in this modelled are measured as benefit

For peer review only

For peer review only

# CHEERS 2022 Checklist

| Topic | No. | Item | Location where item is reported |
|---|---|---|---|
| **Title** | | | |
| **Title** | 1 | Identify the study as an economic evaluation and specify the interventions being compared. | Title, Page 1 |
| **Abstract** | | | |
| **Abstract** | 2 | Provide a structured summary that highlights context, key methods, results, and alternative analyses. | Abstract, Page 1 |
| **Introduction** | | | |
| **Background and objectives** | 3 | Give the context for the study, the study question, and its practical relevance for decision making in policy or practice. | Background, Page 4 |
| **Methods** | | | |
| **Health economic analysis plan** | 4 | Indicate whether a health economic analysis plan was developed and where available. | Methods, Page 5 |
| **Study population** | 5 | Describe characteristics of the study population (such as age range, demographics, socioeconomic, or clinical characteristics). | Methods, Page 7 |
| **Setting and location** | 6 | Provide relevant contextual information that may influence findings. | Methods, Page 6 |
| **Comparators** | 7 | Describe the interventions or strategies being compared and why chosen. | Methods, Page 6-7 |
| **Perspective** | 8 | State the perspective(s) adopted by the study and why chosen. | Methods, Page 5 |
| **Time horizon** | 9 | State the time horizon for the study and why appropriate. | Methods, Page 5 |
| **Discount rate** | 10 | Report the discount rate(s) and reason chosen. | Methods, Page 6 |
| **Selection of outcomes** | 11 | Describe what outcomes were used as the measure(s) of benefit(s) and harm(s). | Methods, Page 5 |
| **Measurement of outcomes** | 12 | Describe how outcomes used to capture benefit(s) and harm(s) were measured. | Methods, Page 10 |
| **Valuation of outcomes** | 13 | Describe the population and methods used to measure and value outcomes. | Methods, Page 10 |



For peer review only - http://bmjopen.bmj.com/site/about/guidelines.xhtml

| Topic | No. | Item | Location where item is reported |
|---|---|---|---|
| **Measurement and valuation of resources and costs** | 14 | Describe how costs were valued. | Methods, Page 11 |
| **Currency, price date, and conversion** | 15 | Report the dates of the estimated resource quantities and unit costs, plus the currency and year of conversion. | Methods, Page 5 |
| **Rationale and description of model** | 16 | If modelling is used, describe in detail and why used. Report if the model is publicly available and where it can be accessed. | Methods, Page 6 |
| **Analytics and assumptions** | 17 | Describe any methods for analysing or statistically transforming data, any extrapolation methods, and approaches for validating any model used. | Methods, Table 1 and Page 6-11 |
| **Characterising heterogeneity** | 18 | Describe any methods used for estimating how the results of the study vary for subgroups. | Methods, Page 12 |
| **Characterising distributional effects** | 19 | Describe how impacts are distributed across different individuals or adjustments made to reflect priority populations. | Methods, Page 12 |
| **Characterising uncertainty** | 20 | Describe methods to characterise any sources of uncertainty in the analysis. | Methods, Page 12 |
| **Approach to engagement with patients and others affected by the study** | 21 | Describe any approaches to engage patients or service recipients, the general public, communities, or stakeholders (such as clinicians or payers) in the design of the study. | Methods, Page 6 |
| Results | | | |
| **Study parameters** | 22 | Report all analytic inputs (such as values, ranges, references) including uncertainty or distributional assumptions. | Results, Table 1 |
| **Summary of main results** | 23 | Report the mean values for the main categories of costs and outcomes of interest and summarise them in the most appropriate overall measure. | Results, Table 2 |
| **Effect of uncertainty** | 24 | Describe how uncertainty about analytic judgments, inputs, or projections affect findings. Report the effect of choice of discount rate and time horizon, if applicable. | Results, Figure 2 |
| **Effect of engagement with patients and others affected by the study** | 25 | Report on any difference patient/service recipient, general public, community, or stakeholder involvement made to the approach or findings of the study | Not applicable as model was based on previous study. |
| **Discussion** | | | |
| **Study findings, limitations, generalisability, and current knowledge** | 26 | Report key findings, limitations, ethical or equity considerations not captured, and how these could affect patients, policy, or practice. | Discussion, Page 13-14 |



For peer review only - http://bmjopen.bmj.com/site/about/guidelines.xhtml

| Topic | No. | Item | Location where item is reported |
|---|---|---|---|
| Other relevant information | | | |
| **Source of funding** | 27 | Describe how the study was funded and any role of the funder in the identification, design, conduct, and reporting of the analysis | Funding statement and acknowledgments, Page 15 |
| **Conflicts of interest** | 28 | Report authors conflicts of interest according to journal or International Committee of Medical Journal Editors requirements. | Competing interests, Page 15 |

*From:* Husereau D, Drummond M, Augustovski F, et al. Consolidated Health Economic Evaluation Reporting Standards 2022 (CHEERS 2022) Explanation and Elaboration: A Report of the ISPOR CHEERS II Good Practices Task Force. Value Health 2022;25. doi:10.1016/j.jval.2021.10.008

For peer review only
